# Retrospectively modeling the effects of increased global vaccine sharing on the COVID-19 pandemic

**DOI:** 10.1038/s41591-022-02064-y

**Published:** 2022-10-27

**Authors:** Sam Moore, Edward M. Hill, Louise Dyson, Michael J. Tildesley, Matt J. Keeling

**Affiliations:** The Zeeman Institute for Systems Biology & Infectious Disease Epidemiology Research, School of Life Sciences and Mathematics Institute, University of Warwick, Coventry, UK

**Keywords:** Infectious diseases, Epidemiology

## Abstract

The severe acute respiratory syndrome coronavirus 2 (SARS-CoV-2) pandemic has caused considerable morbidity and mortality worldwide. The protection provided by vaccines and booster doses offered a method of mitigating severe clinical outcomes and mortality. However, by the end of 2021, the global distribution of vaccines was highly heterogeneous, with some countries gaining over 90% coverage in adults, whereas others reached less than 2%. In this study, we used an age-structured model of SARS-CoV-2 dynamics, matched to national data from 152 countries in 2021, to investigate the global impact of different potential vaccine sharing protocols that attempted to address this inequity. We quantified the effects of implemented vaccine rollout strategies on the spread of SARS-CoV-2, the subsequent global burden of disease and the emergence of novel variants. We found that greater vaccine sharing would have lowered the total global burden of disease, and any associated increases in infections in previously vaccine-rich countries could have been mitigated by reduced relaxation of non-pharmaceutical interventions. Our results reinforce the health message, pertinent to future pandemics, that vaccine distribution proportional to wealth, rather than to need, may be detrimental to all.

## Abstract (web summary)

Retrospective simulation of several global COVID-19 distribution scenarios shows that equitable and altruistic vaccine sharing strategies could have substantially reduced the global burden of SARS-CoV-2 infections and associated mortality.

## Main

Since its emergence in Wuhan, China, at the end of 2019, SARS-CoV-2 has rapidly spread around the world, causing epidemics in nearly every country. As the causative agent of Coronavirus Disease 2019 (COVID-19) disease, the virus has caused considerable morbidity and mortality globally^1^. During 2020, the containment of the pandemic relied predominantly on non-pharmaceutical interventions (NPIs) to limit the spread of infection, thereby reducing severe disease and preventing health services from being overwhelmed^2,3^. Although this approach was broadly effective, it was also economically and socially damaging^4^. During late 2020 and early 2021, numerous vaccines were approved for public use, representing unparalleled development speeds, which has enabled many countries to implement mass vaccination campaigns as a means of mitigation.

By January 2022, approximately 49% of the global population had received a full two doses of a COVID-19 vaccine, although delivery varied greatly between (and within) countries^5,6^. Many high-income countries enjoyed very successful vaccination campaigns, with several exceeding 90% coverage of adults (aged 16 years and older). However, among many low-income and lower-middle-income countries, vaccine availability continues to be considerably more limited, with low-income countries counting for as little as 0.9% of the overall total vaccine deployed^5^.

Low-income and lower-middle-income countries have been mostly dependent on donations from wealthier countries and vaccine sharing schemes, such as the World Health Organization (WHO)-directed COVAX initiative^7^. As an increasing number of countries begin to achieve high levels of vaccine coverage, they face the decision of whether to continue with a nationalistic approach to vaccination—by extending rollout to the young, providing booster jabs to protect against waning immunity and stockpiling surplus resources for future use—or whether to begin donating more vaccines to places where there may be markedly higher payoffs per dose in reducing infection and mortality. Although high-income countries typically have larger elderly populations and, consequently, more individuals who are directly vulnerable to the effects of COVID-19, low-income countries are typically less well equipped to deal with high levels of morbidity. The effects of increased pressure on already limited healthcare resources in low-income countries has had critical impacts on a range of endemic diseases^8,9^, and, without surplus welfare resources available in such countries, NPIs are unsustainable^10^.

In high-income countries that have achieved high levels of vaccination, campaigns have proven highly effective in limiting disease impacts while allowing the relaxation of restrictions and a return to pre-pandemic-like behavior^11–13^. However, the estimated waning of vaccine efficacy^14–16^ has meant that many countries are now investing in booster campaigns^17^, with third and fourth doses becoming widely implemented. The huge numbers of global infections (estimated at more than 14 billion infections to date^18^) have generated considerable opportunities for viral mutation and the emergence of variants that have notable transmission and/or immune escape advantages over ancestral strains^19,20^. Such variants of concern have raised the reproductive number and, hence, prolonged the pandemic by causing new waves of infection. The continued threat of further mutation equates to a large level of uncertainty in future infection patterns. Consequently, although national vaccination campaigns have proven effective in limiting disease impact nationally, epidemic containment may be fully achieved only if high levels of new global infections are avoided, minimizing the threat of generating further variants of concern^21,22^.

Through the use of a detailed global model, matched to country-level COVID-19 disease and incorporating SARS-CoV-2 vaccination data to the end of 2021 in 152 different countries, we explored the effects that increased historic levels of global vaccine sharing would have had on the likely state of the pandemic, projected from early 2020 to the end of 2021. The model simulates five levels of vaccine sharing: from the observed scenario, through sharing, once vaccine-rich countries have offered two doses to all individuals, those over 40 years of age or those over 65 years of age, to full sharing based on protecting either equal proportions of each country or the oldest individuals first across the globe. The model also captures two forms of NPIs: either following the observed controls irrespective of infection levels or with adaptive behavior in which countries that share more vaccine than observed may relax controls more slowly to compensate. We show that increased vaccine sharing may substantially decrease mortality in lower-income countries with only limited increases in some high-income donor nations, provided that the most vulnerable are still vaccinated in a timely manner. We also show the broader potential of more equally distributed vaccination. In return for an increased duration of control measures in the currently vaccine-rich countries, vaccine sharing can substantially decrease the number of overall infections in 2021, reducing the potential for the evolution and spread of increasingly severe variants and, subsequently, substantially improving the outlook of the pandemic both nationally and globally. The main findings and policy implications of this work are provided in Table 1.

**Table 1:** Policy summary

Background	The SARS-CoV-2 pandemic has caused considerable morbidity and mortality worldwide. Although the protection offered by vaccines (and booster doses) offers a method of mitigating the worst effects, by the end of 2021 the distribution of vaccine was highly heterogeneous, with some countries achieving over 90% coverage in adults, whereas others reached less than 2%. In part, this is due to the availability of sufficient vaccine, although vaccine hesitancy also plays a role. We combined estimates of historic SARS-CoV-2 infections and vaccine uptake with an age-structured model for 152 countries to consider the implications of different vaccine sharing policies that go some way to addressing this imbalance.
Main findings and limitations	We calculated that increased vaccine sharing, without any changes to NPIs, would have substantially reduced COVID-19 infection mortality in lower-income countries, although some high-income countries would have had increased mortality unless additional measures were taken. Overall, we estimate that this vaccine sharing scenario would have prevented 1.3 million deaths worldwide (as a direct result of COVID-19) by the end of 2021, although this figure could be substantially increased if increased vaccine sharing from high-income countries had been compensated for with slower easing of NPIs. This global decrease in mortality is due to a combination of greater protection of the most vulnerable and the lower level of global infection, leading to fewer opportunities for new variants to arise. This study is limited to considering vaccine supply constraints, although additional pressures induced by uptake hesitancy and delivery limitations are becoming increasingly relevant.
Policy implications	Although the focus of this work is a retrospective study of the COVID-19 pandemic, there are naturally conclusions to be drawn about national and international policies going forward. Our simulations provide strong analytical evidence to support the message that distributing vaccines across the globe proportional to need, rather than to wealth, can have beneficial effects for all.

## Results

**Fig. 1:**
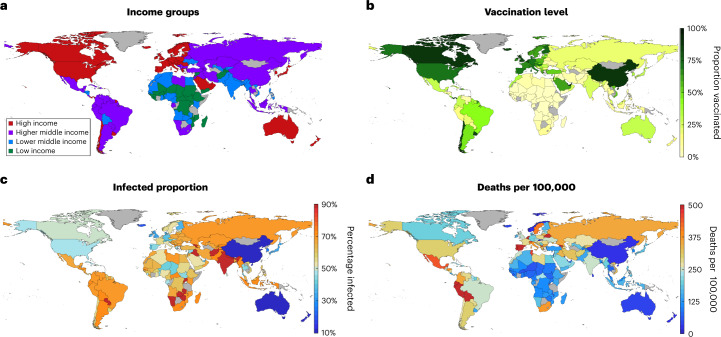
Reference maps for the current, low sharing scenario at the start of 2022. **a**, Income group for each country simulated as defined by the World Bank. **b**, Proportion of each simulated country having received full vaccination (two doses). **c**, Estimated proportion of each simulated country to have been infected by SARS-COV-2. **d**, Estimated total number of deaths per 100,000 due to COVID-19 in each simulated country. In each, gray shading indicates a country that has not been simulated.

**Fig. 2:**
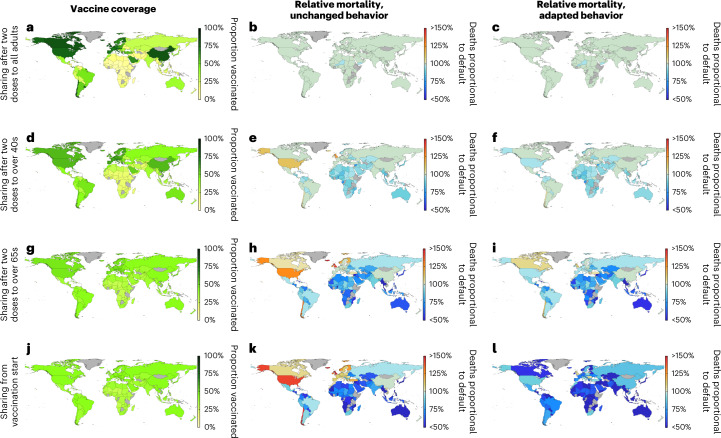
Relative changes in mortality per country under the central vaccine sharing scenarios. Country-level estimates of vaccination coverage at the start of 2022 (**a**,**d**,**g**,**j**); total number of deaths over 2021 relative to the current scenario with unchanged behavior but increased vaccine sharing (**b**,**e**,**h**,**k**); and total number of deaths over 2021 relative to the current scenario with adaptive behavior and increased vaccine sharing (**c**,**f**,**i**,**l**). All results represent medians of 100 simulations, with model fitting spanning the range of uncertainty in infection and mortality estimates for each country. These are presented as caricatures to compare scenario impact with detailed data and associated prediction intervals provided in Supplementary Table 3. Analogous figures for infection estimates are provided in Extended Data Fig. 1.

**Extended Data Fig. 1:**
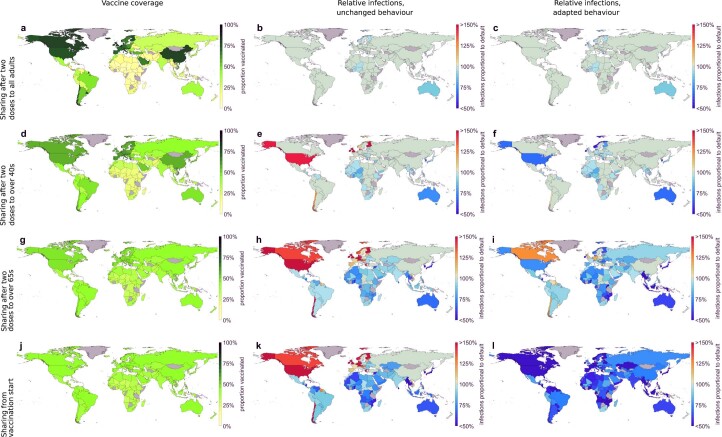
Relative changes in infection per country under central sharing scenarios. Country level estimates of vaccination coverage at the start of 2022 (**a,d,g,j**), total number of infections over 2021 relative to the current scenario with unchanged behaviour but increased vaccine sharing (**b,e,h,k**), and total number of infections over 2021 relative to the current scenario with adaptive behaviour and increased vaccine sharing (**c,f,i,l**). All results represent medians of 100 simulations with model fitting spanning the range of uncertainty in infection and mortality estimates for each country. These are presented as caricatures to compare scenario impact with detailed data and associated prediction intervals provided in Supplementary Table 3. Analogous figures for mortality estimates are given in Fig. 2.

By the end of 2021, nearly 50% of the global population had been fully vaccinated (two doses), although large disparities in coverage across the globe mean that this figure is closer to 75% across high-income countries but less than 2% in many low-income countries (Fig. 1a). Any increased degree of vaccine sharing will at least partially address this balance, potentially generating substantial gains in sparsely vaccinated nations, although inevitably leading to some increase of infection in the most highly vaccinated countries (Fig. 2 and Extended Data Fig. 1). In total, we estimate that a full vaccine sharing scenario would have prevented 295.8 million infections and 1.3 million deaths worldwide (as a direct result of COVID-19) by the end of 2021 without any associated changes in behavior (Fig. 2k, Extended Data Fig. 1k and Supplementary Table 1).

**Fig. 3:**
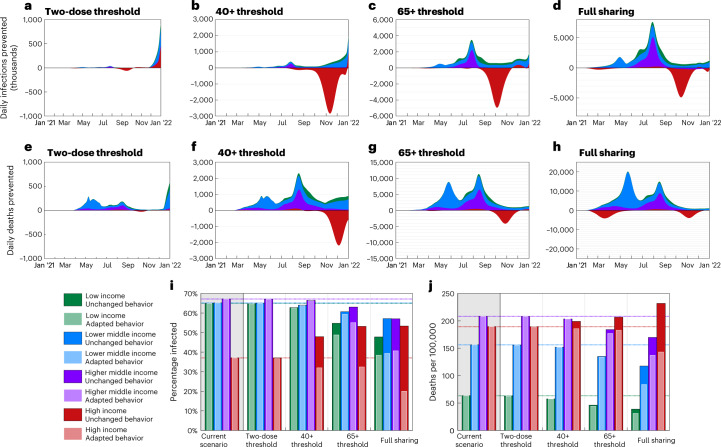
Relative changes over time and total infections and mortality in each economic region under the central vaccine sharing scenarios. **a–h**, Time series plots showing the reduction (positive values) or increase (negative values) in the global number of daily infections and daily deaths compared to the default scenario, each assuming unchanged behavior (equivalent figures for adapted behavior are provided in Extended Data Fig. 2). **i**,**j**, Estimated total proportion of infected and deaths from COVID-19 per 100,000, respectively, until the start of 2022 (so, over all of 2020 and 2021) in each of the economic regions. All results represent medians of 100 simulations, with model fitting spanning the range of uncertainty in infection and mortality estimates for each country. These are presented as caricatures to compare scenario impact with detailed data and associated prediction intervals provided in Supplementary Table 2.

We found that increased vaccine sharing would likely have reduced infections in low-income, lower-middle-income and even higher-middle-income countries across early to mid 2021, with an estimated 25.9%, 12.6% and 15% reductions in these regions, respectively, in the full sharing scenario (Extended Data Fig. 1k and Supplementary Table 2). However, these benefits might have been partially offset by substantial increases in infections experienced in high-income countries later in 2021 as other control measures were relaxed (Fig. 3a–d), with approximately 42.7% more infections in these countries for the full sharing scenario (Supplementary Table 2). Australia and New Zealand appear as notable exceptions to this, as having relatively late starting vaccination programs means few doses are given away in any scenario, and very low infection levels throughout 2021 mean that even small advantages from delayed variant emergence translate into large percentage gains (Extended Data Fig. 1b,e,h,k; results for individual countries are provided in Supplementary Table 3). With later increases in infection, the corresponding mortality is markedly less pronounced (Fig. 3e,f). This is due to most vulnerable people having been offered vaccination in high-income countries by this time in all scenarios, because we assume oldest-first vaccination. Hence, the bulk of this increased infection would be felt by the younger and less vulnerable or in vaccinated individuals. Conversely, the earlier infection prevention in lower-income countries by increased vaccine sharing is likely to have had included many of the elderly and vulnerable and, as such, translated into a substantial saving of lives. As a result, we estimate that there would have been 17.7 fewer deaths per 100,000 globally in the full sharing scenario (a reduction of 13.3%), with lower-income and lower-middle-income countries again seeing the greatest benefits, with 39% and 24.7% mortality reductions, respectively (Supplementary Tables 1 and 4).

The bulk of these benefits are not seen in later sharing scenarios (where vaccine is only shared once particular age groups are completed nationally; Figs. 2b,e and 3a,b,e,f); this is because relatively few countries reached the 40+ age threshold before mid to late 2021—by which time many low-income countries would have also already experienced large amounts of infection. Hence, the largest gains are seen only in earlier sharing scenarios (65+ age threshold/full sharing; Figs. 2h,k and 3c,d,g,h). All sharing scenarios result in slightly higher global vaccination coverage (49.6% of the population receiving at least two doses by the end of 2021 versus 44.4% in the current scenario; Supplementary Table 2) due to prioritizing initial vaccine doses worldwide over booster doses and stockpiling in the wealthiest countries. Consequently, the higher impact per dose of initial vaccine doses above booster doses results in some evidence of potential further benefits in the late stages of 2021 in all sharing scenarios, particularly as new emerging variants drive further transmission increases (Fig. 3a,e,i,j).

**Fig. 4:**
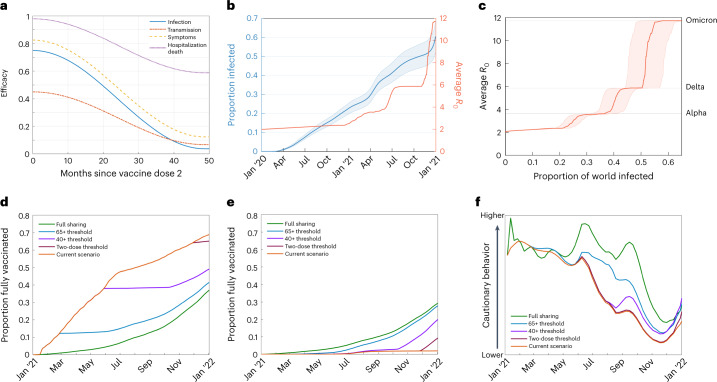
Figures showing model parameters. **a**, Efficacy profiles used for vaccination after the second dose of vaccination. **b**,**c**, Estimates for the infected proportion of the global population and average *R*_0_ of SARS-CoV-2 strains in circulation. Median infection estimates are indicated by the thicker central line, with a 95% confidence interval for infected proportions indicated by thinner outer lines. Panel **b** shows separately the correlation between infection numbers with time and *R*_0_ with time, and panel **c** shows the correlation between infected proportion and average *R*_0_. **d**,**e**, Example plots comparing levels of vaccination over time in each scenario considered for a highly vaccinated country (United Kingdom) and a country with limited vaccination (Kenya). **f**, Example plot for a highly vaccinated country (United Kingdom) of levels of cautionary behavior used for each level of vaccine sharing, for scenarios with adapted behavior.

**Extended Data Fig. 2:**
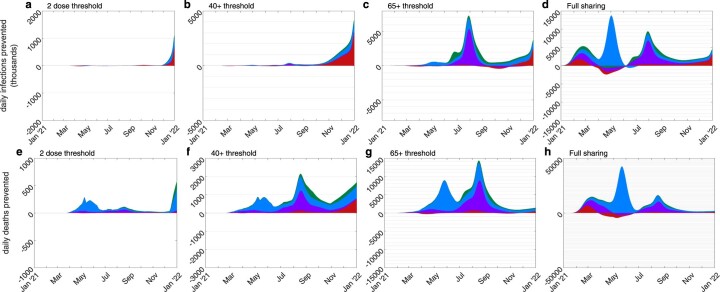
Relative changes in infection and mortality over time in each economic region for scenarios with adapted behaviour. Time series plots showing the reduction (positive values) or increase (negative values) in the global number of daily infections (**a**–**d**) and daily deaths (**e**–**h**) compared to the default scenario, each with adapted behaviour (equivalent figures for unchanged behaviour are given in Fig. 3). All results represent medians of 100 simulations with model fitting spanning the range of uncertainty in infection and mortality estimates for each country. These are presented as caricatures to compare scenario impact with detailed data and associated prediction intervals provided in Supplementary Table 2.

If the increased infection seen in high-income countries due to increased vaccine sharing resulted in extended behavioral caution (adapted behavior or longer use of NPIs), we estimate a much greater reduction in global infections than in scenarios where behavior remains unchanged (Fig. 3i,j). We estimate that the global population infected and mortality rates by the end of 2021 would have been substantially reduced for the full vaccine sharing strategy scenario (29.1% infected, 84 deaths per 100,000; Fig. 2l, Extended Data Fig. 1l and Supplementary Table 2) compared to the default scenario (48.4% infected, 133.1 deaths per 100,000). This is driven by the benefits of early vaccine sharing for lower-income countries persisting throughout 2021 (Extended Data Fig. 2), reducing infection and, hence, the potential for the emergence and spread of variants. This would create a positive feedback loop with less infection causing fewer variants and less increase in the basic reproduction number (*R*_0_), itself leading to less infection; while infection levels are kept below ~35%, there is not the opportunity for the Delta variant to arise, so we do not observe the associated large increase in *R*_0_. (Fig. 4b,c).

**Extended Data Fig. 3:**
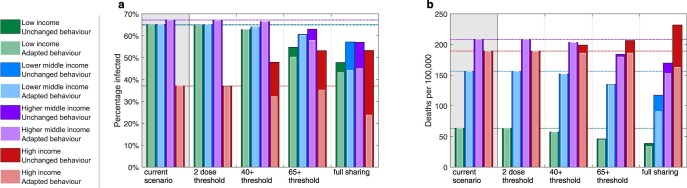
Total infections and mortality per economic region under scenarios with lower level behaviour adaptation. Plots comparing estimates based on unchanged behaviour and lower level adapted behaviour. Estimated total proportion infected (**a**) and deaths from COVID-19 per 100,000 (**b)** until the start of 2022 (so over all of 2020 and 2021) in each of the economic regions. All results represent medians of 100 simulations with model fitting spanning the range of uncertainty in infection and mortality estimates for each country. These are presented as caricatures to compare scenario impact with detailed data and associated prediction intervals provided in Supplementary Tables 5 and 6.

**Extended Data Fig. 4:**
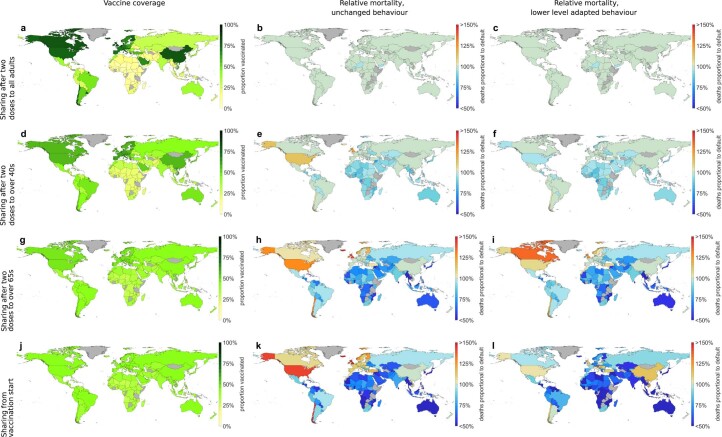
Relative changes in mortality per country under scenarios with lower level behaviour adaptation. Plots comparing estimates based on unchanged behaviour and lower level adapted behaviour. Country level estimates of vaccination coverage at the start of 2022 (**a,d,g,j**), total number of deaths over 2021 relative to the current scenario with unchanged behaviour but increased vaccine sharing (**b,e,h,k**), and total number of deaths over 2021 relative to the current scenario with lower level adaptive behaviour and increased vaccine sharing (**c,f,i,l**). All results represent medians of 100 simulations with model fitting spanning the range of uncertainty in infection and mortality estimates for each country. These are presented as caricatures to compare scenario impact with detailed data and associated prediction intervals provided at an income group level in Tables S5 and S6.

Changes in behavior are, however, very difficult to predict; although reduced vaccination and increased infection are likely to have had a behavioral impact, the extent is highly speculative. At a country level, the interactions among vaccination use, population dynamics and NPIs are highly complex; in the scenarios presented, although many highly vaccinated European countries benefit greatly from adapting behavior, benefits are considerably reduced in countries with moderate but later vaccination (such as Lithuania, Serbia and Romania) that are minimally impacted by vaccine sharing. Overall trends, however, remain constant across simulations, and a smaller behavioral response would result in infection levels between the adapted behavior and unchanged behavior scenarios presented (Extended Data Figs. 3 and 4 and Supplementary Table 5). If NPIs increased at 50% of the rate presented in the central adapted behavior scenario, we estimate a more modest reduction in infection (33% down from 40% with full sharing; Supplementary Table 6). Conversely, a stronger behavioral response would be expected to generate even fewer infections globally.

**Extended Data Fig. 5:**
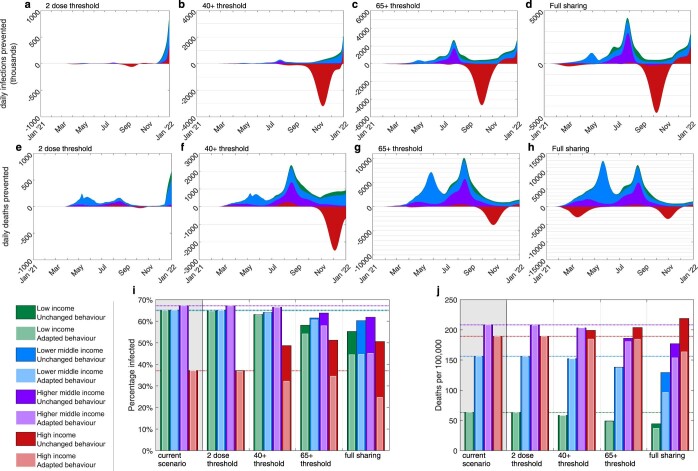
Relative changes over time and total infections and mortality in each economic region for scenarios with age biased vaccine distribution. Plots presenting estimates based on scenarios with age biased vaccine redistribution. Panels **a-h** show time series plots showing the reduction (positive values) or increase (negative values) in the global number of daily infections and daily deaths compared to the default scenario, each assuming un-adapted behaviour. Panels **i** and **j** show estimated total proportion infected and deaths from COVID-19 per 100,000 respectively until the start of 2022 (so over all of 2020 and 2021) in each of the economic regions. All results represent medians of 100 simulations with model fitting spanning the range of uncertainty in infection and mortality estimates for each country. These are presented as caricatures to compare scenario impact with detailed data and associated prediction intervals provided in Supplementary Tables 7 and 8.

**Extended Data Fig. 6:**
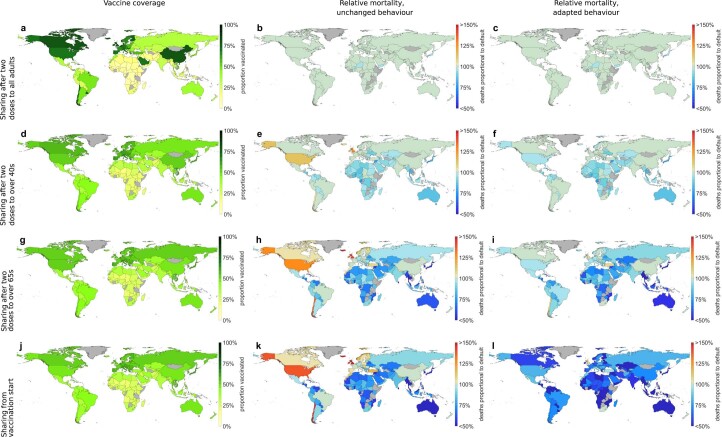
Relative changes in mortality per country under scenarios with age biased vaccine distribution. Plots presenting estimates based on scenarios with age biased vaccine redistribution. Country level estimates of vaccination coverage at the start of 2022 (**a,d,g,j**), total number of deaths over 2021 relative to the current scenario with unchanged behaviour but increased vaccine sharing (**b,e,h,k**), and total number of deaths over 2021 relative to the current scenario with adaptive behaviour and increased vaccine sharing (**c,f,i,l**). All results represent medians of 100 simulations with model fitting spanning the range of uncertainty in infection and mortality estimates for each country. These are presented as caricatures to compare scenario impact with detailed data and associated prediction intervals provided at an income group level in Supplementary Table 7.

Although we concentrated on vaccine sharing to increase equity in the proportion of the adult population of each country that has been vaccinated, due to the considerably increased vulnerability seen with age, vaccine sharing that accounts for age demographics may present an effective alternative redistribution strategy. High-income countries typically have older populations than low-income countries, and, as such, age-biased vaccine sharing strategies will typically see less substantial vaccine redistribution, with wealthy countries reserving an increased number of doses. In the full sharing, unchanged behavior scenario, we see that age-biased redistribution results in small increases in mortality in high-income countries (a 16% mortality increase down from 22% relative to the current scenario, based on actual vaccine distribution to date) compensated by reduced reductions in low-income countries (a 30% reduction down from 39% relative to the current scenario), when compared to non-age-biased sharing (Extended Data Figs. 5 and 6 and Supplementary Tables 7 and 8).

## Discussion

Although the focus of this work is retrospective, considering the merits and consequences of a more equitable sharing of vaccine between nations, conclusions can be drawn about national and international policies going forward. The message for any emerging outbreak is clear: distributing vaccines across the globe proportional to need, rather than to wealth, will have beneficial effects for all. This notion resonates with the ongoing monkey pox outbreak where vaccines are initially being deployed in high-income counties to contain relatively small outbreaks rather than targeting the larger reservoir of infection in Africa^23^. However, the implications for SARS-CoV-2 are less clear. The high volumes of vaccine already used in many countries means that there is the potential for large waves of future disease within their vulnerable populations if vaccine protection is not maintained—a situation that is made more acute by the observed rapid waning of vaccine protection^14,24^. However, preventing the rapid emergence of new variants and, hence, the long-term control of COVID-19 relies on reducing the global burden of infection, creating a tension between national (short-term) and international (long-term) perspectives that is greater now than at any time in the pandemic. Although our model suggests that global vaccine equity and reduced global burden of infection is of benefit to all, it may necessitate the short-term re-imposition of some non-pharmaceutical mitigation measures (with their associated economic consequences and social disruption) instead of additional booster vaccines in some countries.

We show that a more equitable approach to global vaccine distribution over the course of 2021 would have reduced the level of global mortality associated with COVID-19 disease. Our conclusions are based on simulations fitted to historical infection and mortality estimates and from varying the distribution of vaccination between countries while maintaining total vaccine supply. Similar studies have previously been run by Wagner et al.^21^, who used a simple two-country conceptual model, and Watson et al.^25^, who followed a similar approach to estimate the impact on global mortality had overall vaccine supply varied. Any historical deviation in vaccine distribution would likely precipitate a range of consequences, including changes in other policy areas, social behavior, overall vaccine uptake, patterns of viral spread and variant evolution. Although such compounding factors are difficult to predict, the simulation results presented here include potential changes to policy (or behavior) and account for the consequences for variant accretion, increasing the robustness of our findings.

We estimate that the greatest reductions in infection and mortality are associated with vaccine sharing earlier in the pandemic, with less extensive or delayed strategies presenting more modest benefits. In our model, given the high transmissibility of SARS-COV-2 infection, countries without high and early vaccine coverage are likely to rapidly incur high infection levels and, hence, substantial population immunity; as such, the effects of late vaccine sharing to these countries are much reduced. In addition, due to limited delivery capacities and increasing public distrust of vaccination^26^, starting vaccination earlier may have led to more successful vaccination campaigns in general. However, with increasing transmission and possible immune escape from new variants^22^ and the risk of waning efficacy, vaccine sharing remains important: our model suggests that even late vaccine sharing, once wealthy countries had delivered all second doses, would have seen sizeable benefits in late 2021 and into 2022.

We estimate that increased vaccine sharing would have provided large benefits in low-income and lower-middle-income countries; this benefit comes at a cost to some high-income countries where increased or prolonged use of NPI measures would have been required to suppress disease in the short term. This substantial reduction in disease burden could have reduced the unmanageable waves of disease experienced by many of the poorest countries that are least well equipped to manage the pandemic. In addition, as high sharing scenarios delay infections until later in the year, these infections would have occurred once knowledge and treatments had improved and so may have been better managed.

This study is subject to several limitations. First, we concentrated on supply constraints. Assuming that a fixed amount of vaccine was available throughout 2021, the key issue addressed is where this should have been deployed. Supply has historically been a major factor causing heterogeneity in worldwide coverage^27^—when vaccines first became available, the limited quantities produced were purchased primarily by wealthier nations. A confounding factor in this calculation is that, because the countries producing and financing the vaccines have typically already had access to large amounts of vaccine, there has been little incentive to substantially increase production^28^. One might hypothesize that increased sharing might have encouraged additional resources to be put into production, increasing the overall volume of vaccines available.

In this analysis, we also assumed that vaccine efficacy profiles used are uniform across nations; however, many lower-income countries rely on substantially less effective vaccines than the more desirable counterparts employed by high-income countries^29^. During the course of simulation, we tested sensitivity to vaccine efficacy, and, as expected, if redistributed vaccines were of lower or higher efficacy, the benefits of sharing would be reduced or increased, respectively. Imbalances in vaccine efficacy may then mean that the heterogeneity in effective vaccine coverage would be even greater than assumed, and increasing vaccine sharing to address this imbalance would be even more critical.

With numerous different vaccines now being produced and the success of the COVAX scheme increasing vaccine availability^7^, limitations surrounding delivery and uptake are becoming increasingly important^30^. In our model, it is unsurprising that, if the level of vaccine uptake resulting from increased supplies was lower than presented, the benefits of sharing would be comparatively reduced. Many lower-income countries lack the infrastructure needed to rapidly deliver vaccines on the scale required, especially where there are large, hard-to-reach population sectors. Similarly, although vaccine hesitancy has been a recognized problem in all nations, in countries where public health messaging and education is limited, hesitancy is becoming a severe limiting factor for increased vaccine coverage^26,31,32^. Future support may, therefore, need to include assistance with vaccine delivery and logistical support in addition to the provision of vaccine doses.

Finally, we have not explored the major threat of variants that escape vaccine and/or naturally induced immunity, owing to their lack of substantial impact during 2021 (ref. ^33^). The emergence of Omicron in November 2021 has posed just such a threat^34–36^, with the potential for large waves of infection and the need to re-vaccinate some vulnerable populations. This emergence strengthens the arguments for vaccine sharing as a means of reducing the global levels of infection and, hence, retarding the accumulation of new variants.

Vaccines generally offer greater protection against severe disease than infection^14,37^, and the effects against severe disease are likely to be more robust against both waning immunity and vaccine escape^14,24^. Hence, deploying vaccines to regions where there remains a high proportion of unprotected vulnerable individuals would have a much greater impact per dose than extending vaccination in countries that have already protected most of their vulnerable population. A complication to this vaccine equity picture is that the number of elderly and vulnerable individuals is larger in high-income countries, although reduced welfare resources and limited access to effective treatments, as often seen in low-income countries, make a true determination of vaccine need challenging. However, our model-based results reinforce the global public health message that vaccine nationalism (protecting one’s own country to the detriment of others) not only leads to greater levels of infection and mortality worldwide but also adversely impacts all countries in the long term^38–41^.

## Methods

This study was based on simulations using a mathematical model of SARS-CoV-2 transmission and COVID-19 outcomes, using data pre-existing in the public domain. As such, there were no relevant ethical regulations to consider. Simulations included 152 different countries, each with its own parameters reflecting demographics and social structure. The countries were simulated in parallel, using independent, age-structured, deterministic, compartmental infection models but coupled by the global evolution of new variants and the sharing of vaccines when national conditions are met. Given the strong correlation between per capita income and the level of COVID-19 vaccination^42^, we partitioned the 152 countries simulated into the four income group classifications given by the World Bank^43^ (Fig. 1a). Exclusions (gray regions in Fig. 1, listed with Supplementary Table 3), made only for countries where data are missing from sources used, are assumed average for each income group.

Individuals within each country were classified as susceptible (*S*), exposed (*E*), infectious and symptomatic (*I*), infectious and asymptomatic (*A*) or recovered (*R*). We used a set of ordinary differential equations to describe the flow of individuals between these compartments. Susceptible individuals were subjected to a force of infection proportional to *I* + *τ*^*a*^*A*, where *τ*^*a*^ is an age-dependent discounting factor used to represent reduced transmission from asymptomatic individuals compared to symptomatic individuals; superscripts here denote 5-year age bands. The exposed class was further subdivided into three separate states, *E*_1_, *E*_2_ and *E*_3_, meaning that, in a stochastic formulation, the distribution of the latent period would become an Erlang distribution, creating more realistic infection time scales.

Age is recognized to play an important role in the dynamics of the SARS-CoV-2 epidemic, strongly influencing both the outcome after infection and the characteristic social mixing behavior^44^ that facilitates transmission. These age-based heterogeneities were captured by stratifying the modeled populations into 5-year age groups using country-level data on age demographics^45^, each with their own parameters for susceptibility, the occurrence of symptoms and the risk of severe disease.

Throughout the pandemic, most countries have responded to rising levels of disease with mitigation measures, including social distancing, quarantining, mandatory mask wearing and contact tracing^46^, and increased caution among residents may act to substantially slow viral spread^47^. To account for these effects, epidemiologically relevant contacts within each country are varied in a time-varying manner by a country-specific control factor to allow for the effects of NPI measures and social caution, and epidemiologically relevant contacts within each country are reduced in a time-varying manner by a country-specific control factor *ϕ*. The resulting set of differential equations (ODEs) used to model the population over time, *t*, for each country are, hence, formulated as follows:

1 $$\begin{array}{l}\frac{{{\mathrm{d}}{{S}}^{{a}}}}{{{\mathrm{d}}{{t}}}} = - \lambda ^{{a}}\beta ^{{a}}{{S}}^{{a}}\quad {{{\mathrm{where}}}}\quad \lambda ^{{a}} = \mathop {\sum}\limits_{{b}} {\phi {{M}}^{{{ab}}}\left( {{{I}}^{{a}} + \tau ^{{a}}{{A}}^{{a}}} \right)} \\ \frac{{{\mathrm{d}}{{E}}_1^{{a}}}}{{{\mathrm{d}}{{t}}}} = \lambda ^{{a}}{{S}}^{{a}} - \alpha {{E}}_1^{{a}}\\ \frac{{{\mathrm{d}}{{E}}_2^{{a}}}}{{{\mathrm{d}}{{t}}}} = \alpha {{E}}_1^{{a}} - \alpha {{E}}_2^{{a}}\\ \frac{{{\mathrm{d}}{{E}}_3^{{a}}}}{{{\mathrm{d}}{{t}}}} = \alpha {{E}}_2^{{a}} - \alpha {{E}}_3^{{a}}\\ \frac{{{\mathrm{d}}{{I}}^{{a}}}}{{{\mathrm{d}}{{t}}}} = {{d}}^{{a}}\alpha {{E}}_3^{{a}} - \gamma ^{{a}}{{I}}^{{a}}\\ \frac{{{\mathrm{d}}{{A}}^{{a}}}}{{{\mathrm{d}}{{t}}}} = \left( {1 - {{d}}^{{a}}} \right)\alpha {{E}}_3^{{a}} - \gamma ^{{a}}{{A}}^{{a}}\\ \frac{{{\mathrm{d}}{{R}}^{{a}}}}{{{\mathrm{d}}{{t}}}} = \gamma ^{{a}}\left( {{{I}}^{{a}} + {{A}}^{{a}}} \right).\end{array}$$

The core infection parameters used were assumed to be the same across all countries. These include age-dependent variables for transmission, *β*; the probability of exhibiting symptoms, *d*; the progression rate between exposure and infection, *α*; the recovery rate, *γ*; and the reduction in asymptomatic transmission, *τ*. Estimates for these values were fitted from early age-stratified United Kingdom case data to match growth rate, reproductive number and age profiles of infection between the start of the pandemic and the emergence of the Alpha variant in early 2021. Country models vary in demographics, informed by WHO estimates^45^ and mixing patterns, based on contact matrices, *M*, described by Prem et al.^44^, vaccination levels, mitigating control factors, *ϕ*, as well as disease outcomes.

### Statistical analysis

Parameters for national control measures and death rates were determined as maximum likelihood estimates, using data estimates for the total number of all new infections and deaths (together with levels of uncertainty) as proposed by the Institute for Health Metrics and Evaluation (IHME)^18^. Due to inconsistencies and under-reporting of COVID-19 metrics in many countries, rather than relying on official reports, their estimates are made based on excess mortality statistics, comparing death rates in each country during the pandemic to historical data, tracking past trends and seasonality. Other similar excess mortality estimates have been made elsewhere with reasonable consistency; for instance, Karlinsky et al.^48^ estimated 160,000 deaths in South Africa by 27 June 2021 (60,000 reported) compared to the IHME estimate of 156,373 deaths (with a high and low estimate range of 90,221 to 258,352).

Uncertainty in these values is accounted for by taking 100 independent random samples informed by the high and low IHME estimates and propagating these samples through the fitting and simulation to generate means and 95% prediction intervals. Sample size was chosen to cover the range of parameter estimates while remaining sufficiently computationally inexpensive, and no statistical method was used to predetermine this sample size. No data were excluded from the analyses, and the experiments were not otherwise randomized. The investigators were not blinded to allocation during experiments and outcome assessment.

### Variants

Across the course of the pandemic, there has been a substantial rise in the level of transmissibility of the dominant SARS-CoV-2 variant. Initially, when COVID-19 was first detected in China, the basic reproductive number, *R*_0_, was estimated between 2 and 2.4 (ref. ^49^). Transmissibility has seen three major step changes due to the Alpha variant (*R*_0_ ≈ 4–5) at the end of 2020; the Delta variant (*R*_0_ ≈ 5–7)^50,51^ becoming dominant in many countries by early summer 2021; and the emergence of the Omicron variant (*R*_0_ ≈ 10−16) at the end of 2021.

By considering the global proportion of each variant (as averaged across countries for which such data are available from the GISAID database^52^, assuming wild-type has *R*_0_ ≈ 2.2, Alpha variant has *R*_0_ ≈ 4, Delta has *R*_0_ ≈ 6 and Omicron has *R*_0_ ≈ 12), we may visualize the trend of increasing *R*_0_ (Fig. 4b) and the associated level of infection up to that time. The relationship between total historic infections (blue) and the average basic reproductive number (red) is then used to realize the impact of varying infection levels on variant emergence in the simulations—relating a given level of historic infection to an average basic reproductive ratio due to the emergence of new variants (Fig. 4c).

### Vaccination and sharing scenarios

We make the assumption that all countries aim to eventually achieve vaccine coverage in all individuals from the age of 12 and older, with a 90% uptake for those older than 60 years and 80% for those younger (although vaccine hesitancy may present substantial difficulty in achieving this in some nations). We also assume that an oldest-first approach to vaccine distribution is used in all nations, delivering vaccines to the most vulnerable first^53^. Although deviation from this approach has been seen in some countries that have chosen to prioritize essential workers or key disease spreaders (including those, such as taxi drivers, in high-contact professions), the strategy of oldest-first is the most widely employed^46^.

Vaccination is assumed to provide individual protection against four measures: susceptibility, onward transmission, symptom probability and hospitalization/death. These are based on efficacy characteristics similar to one or two doses of ChAdOx1-S/nCoV-19 (AstraZeneca) vaccine^14^—this being one of the most widely distributed and well-studied COVID-19 vaccines to date^27^. This is an approximation of the heterogeneous global picture^27^, with some vaccines (such as Sinopharm) considered to provide lower protection^54^ and the widely deployed Johnson & Johnson vaccine requiring only a single dose^55^. Specifically, before waning, we take vaccine efficacy against:

Infection: 60% one dose, 75% two doses

Transmission: 45% one dose, 45% two doses

Symptoms: 60% one dose, 83% two doses

Severe disease: 80% one dose, 98% two doses

In addition, efficacy can also vary with age, dose interval and between variants. All four forms of vaccine protection are assumed to wane over time, from a maximum shortly after the second dose to minimum levels 4 years (48 months) later (Fig. 4a). Countries completing two-dose vaccination coverage (subject to assumptions made for eligibility and uptake), and with sufficient vaccine supply, are assumed to commence delivery of booster vaccinations to all individuals at 6-month dose intervals, again in oldest to youngest priority order. Booster doses when delivered are taken to reset waning back to the maximum efficacy level. Alongside a default, low sharing scenario reflecting actual historical vaccine delivery, several more collaborative strategies that consider alternative distribution scenarios over the course of 2021 are investigated:

Current scenario, low sharing: Past reports for daily vaccines administered are followed in each country.

Two-dose threshold: The simulation progresses in each country, with daily vaccination numbers equal to the current scenario until the two-dose vaccination program is completed (subject to uptake assumptions). Subsequent daily vaccine deliveries from that country are then divided between all countries proportional to the remaining number of individuals pending vaccination (again, within the assumptions made for eligibility and uptake).

40+ threshold: Similar to the two-dose threshold scenario except that vaccine sharing begins in each country after a two-dose vaccination is completed for all individuals 40 years of age and older.

65+ threshold: Similar to the two-dose threshold scenario except that vaccine sharing begins in each country after a two-dose vaccination is completed for all individuals 65 years of age and older.

Full sharing: Vaccine sharing begins at the start of 2021 with all vaccination pooled and divided between all countries proportional to the number of unvaccinated individuals remaining in each.

In the central scenarios presented, vaccination is redistributed proportionally to the number of eligible individuals in each country who remain unvaccinated, although we additionally present strategies (Extended Data Figs. 5 and 6) where vaccine redistribution is age-biased, dependent on the number of unvaccinated individuals in each 5-year age bracket weighted by vulnerability. To incorporate the effects of variants and changing vaccination levels, the model is run in 2-day time steps. In each time step, countries are simulated individually by solving the set of ODEs (Eq. (1)). Between steps, we update a transmission scaling parameter due to variants, *ω*, as well as country-specific vaccine parameters, *v*_inf_, *v*_tra_, *v*_sym_ and *v*_sev_, scaling infection, transmission, probability of infection being symptomatic and probability of hospitalization/death, respectively, for each age group. These are applied to the susceptibility, *v*_inf_*M*, transmission, *ωv*_tra_*β*, and symptomatic probability, *v*_symp_*d*, parameters. The final vaccine parameter, *v*_sev_, is used to scale severe disease outcomes, used in the calculation of numbers of hospitalizations, *H*, and deaths, *D*, between time steps as follows:

2 $$\begin{array}{l}H\left( {t + l_{\mathrm{h}}} \right) = v_{\mathrm{{sev}}}\left( t \right)\eta _{\mathrm{h}}R\left( t \right)\\\end{array}$$

3 $$\begin{array}{l}D\left( {t + l_{\mathrm{d}}} \right) = v_{\mathrm{{sev}}}\left( t \right)\eta _{\mathrm{d}}^aR\left( t \right)\end{array}$$

where *η*_h_/*η*_d_ denotes the country-specific probability of hospitalization/death and *l*_h_/*l*_d_ gives the time lag to hospitalization/death, respectively.

Each vaccine parameter is calculated as

4 $$v\left( t \right) = \mathop {\sum}\limits_{s = 0}^t {\upupsilon} \left( s \right)w\left( {t - s} \right),$$

using the amount of vaccination available, *w*(*t*), at time step *t* for each country (as determined by the strategy being followed), together with decaying efficacy parameter, υ, with profiles shown in Fig. 4a.

The variant transmission parameter is updated to a value relative to estimated global variant prevalence at the time point when total infections up to that time step in simulation match total past infection estimates—that is, following the trend of the red line in Fig. 4c.

### Adapted behavior

In scenarios where some countries have reduced vaccine supply due to increased sharing, it is likely that, without a change to behavior or controls, infections would increase compared to the current scenario. We, therefore, perform two projections, one where the behavior follows inferred levels irrespective of infections and one where behavior adapts. We implement this behavioral response by increasing/decreasing the control parameter, *ϕ*, for each country that is sharing vaccines, dependent on whether the number of active infections is increasing or decreasing (subject to a 5-day time lag to reflect delays in detection and reaction). An example showing behavior adaptation is given in Fig. 4f.

### Reporting summary

Further information on research design is available in the Nature Research Reporting Summary linked to this article.

## Online content

Any methods, additional references, Nature Research reporting summaries, source data, extended data, supplementary information, acknowledgements, peer review information; details of author contributions and competing interests; and statements of data and code availability are available at 10.1038/s41591-022-02064-y.

## Supplementary information

Supplementary Information Supplementary Tables 1–8

Reporting Summary

## Data Availability

The study was based on data from a variety of publicly available sources: population demographic data provided by the WHO^45^; income group classifications provided by the World Bank^43^; COVID-19 vaccine deployment provided by Our World in Data^5^; COVID mortality and infection estimates to date made by the Institute for Health Metrics and Evaluation^18^; and data on COVID-19 variants collated by GISAID^20^.

## Code availability

Model code was written in MATLAB R2020a and is available in the following repository: https://github.com/sammoore25/The-impacts-of-increased-global-vaccine-sharing-in-the-COVID-19-pandemic/.
