# Author Correction: Retrospectively modeling the effects of increased global vaccine sharing on the COVID-19 pandemic

**DOI:** 10.1038/s41591-023-02303-w

**Published:** 2023-03-27

**Authors:** Sam Moore, Edward M. Hill, Louise Dyson, Michael J. Tildesley, Matt J. Keeling

**Affiliations:** The Zeeman Institute for Systems Biology & Infectious Disease Epidemiology Research, School of Life Sciences and Mathematics Institute, University of Warwick, Coventry, UK

Correction to: *Nature Medicine* 10.1038/s41591-022-02064-y, published online 27 October 2022.

In the version of this article initially published, in the Figure 1a map, Vietnam was incorrectly shaded in red and has now been corrected to blue (as a lower middle income country) in the HTML and PDF versions of the article.

