# Supplementary material for: Retrospectively modeling the effects of increased global vaccine sharing on the COVID-19 pandemic
Source: Nat Med. 2022 Oct 27;28(11):2416–23. doi: 10.1038/s41591-022-02064-y (PMC9671807; doi:10.1038/s41591-022-02064-y)
Supplement: Supplementary file 1 — Supplementary Tables 1–8 [file 41591_2022_2064_MOESM1_ESM.pdf]

nature medicine

Article https://doi.org/10.1038/s41591-022-02064-y

Retrospectively modeling the effects of increased global vaccine sharing on the COVID-19 pandemic

In the format provided by the authors and unedited

nature medicine

Article https://doi.org/10.1038/s41591-022-02064-y

Retrospectively modeling the effects of increased global vaccine sharing on the COVID-19 pandemic

In the format provided by the authors and unedited

Supplementary Information: Retrospectively modelling the effects of increased global vaccine sharing on the COVID-19 pandemic

Sam Moore*[12], Edward M. Hill[12], Louise Dyson[12], Michael J. Tildesley[12], Matt J. Keeling[12].

1. The Zeeman Institute for Systems Biology & Infectious Disease Epidemiology Research, School of Life Sciences and Mathematics Institute, University of Warwick, Coventry, CV4 7AL, United Kingdom.

2. Joint UNIversities Pandemic and Epidemiological Research.

* Corresponding Author. Email: Samuel.E.Moore@warwick.ac.uk

1 Data for central scenarios

In tables S1, S2, S3 and S4, we present data for the central vaccine sharing scenarios, as presented in the main manuscript.

Region	Strategy	Infections averted (millions)		Mortalities averted (thousands)	
		unchanged behaviour	adapted behaviour	unchanged behaviour	adapted behaviour
World	2 dose threshold	8.3 (5.9 – 12.0)	12.0 (9.5 – 14.1)	27.2 (24.8 – 31.9)	28.8 (24.4 – 31.8)
	40+ threshold	-66.6 (-76.1 – -55.1)	123.4 (107.9 – 135.7)	113.9 (89.3 – 141.2)	269.5 (248.3 – 299.0)
	65+ threshold	67.2 (44.5 – 99.3)	492.3 (452.2 – 561.4)	864.2 (786.6 – 942.0)	1306.4 (1203.3 – 1419.8)
	Full sharing	295.8 (221.0 – 408.4)	1467.7 (1368.3 – 1531.5)	1337.7 (1145.3 – 1494.3)	3727.7 (3262.4 – 3959.8)
Low income	2 dose threshold	1.6 (1.2 – 2.3)	1.7 (1.2 – 2.2)	4.1 (3.4 – 4.7)	4.1 (3.3 – 4.6)
	40+ threshold	12.8 (10.5 – 15.7)	14.2 (11.5 – 17.5)	35.7 (31.1 – 39.9)	35.7 (31.3 – 39.9)
	65+ threshold	60.7 (55.1 – 69.5)	97.5 (89.5 – 105.3)	107.0 (94.5 – 124.9)	119.5 (107.7 – 141.0)
	Full sharing	103.8 (97.0 – 112.0)	164.1 (140.1 – 180.6)	153.2 (138.7 – 175.5)	199.4 (176.1 – 230.6)
Lower middle income	2 dose threshold	4.4 (3.5 – 5.8)	4.7 (3.9 – 5.9)	16.5 (15.1 – 19.0)	16.4 (13.9 – 17.9)
	40+ threshold	32.1 (27.9 – 36.5)	36.7 (31.4 – 41.2)	119.3 (108.6 – 132.7)	118.4 (109.1 – 135.3)
	65+ threshold	127.2 (120.1 – 140.2)	169.4 (151.8 – 207.8)	595.5 (554.7 – 651.0)	639.2 (593.1 – 690.9)
	Full sharing	231.0 (210.5 – 261.3)	728.9 (647.5 – 792.6)	1088.1 (1005.5 – 1190.5)	1994.9 (1734.1 – 2247.9)
Higher middle income	2 dose threshold	0.8 (0.6 – 1.9)	0.8 (0.3 – 2.0)	6.4 (5.5 – 7.8)	6.3 (5.4 – 7.3)
	40+ threshold	9.7 (7.4 – 13.9)	11.4 (9.1 – 17.8)	71.9 (62.1 – 86.1)	72.3 (62.1 – 85.8)
	65+ threshold	62.4 (47.9 – 79.8)	175.4 (155.6 – 202.1)	358.4 (312.9 – 420.1)	461.3 (390.8 – 554.8)
	Full sharing	148.4 (96.4 – 205.3)	388.7 (347.9 – 425.6)	574.4 (494.6 – 676.3)	1020.0 (845.0 – 1190.6)
High income	2 dose threshold	1.3 (-0.1 – 2.4)	4.6 (3.5 – 5.7)	0.3 (-0.4 – 1.2)	2.0 (1.3 – 2.6)
	40+ threshold	-121.0 (-132.3 – -112.9)	59.7 (53.0 – 66.9)	-113.8 (-124.1 – -95.5)	41.9 (33.8 – 47.6)
	65+ threshold	-182.6 (-196.9 – -173.4)	53.4 (37.8 – 69.1)	-198.1 (-220.0 – -179.0)	76.1 (60.0 – 92.3)
	Full sharing	-184.0 (-195.0 – -162.3)	188.2 (140.8 – 207.8)	-484.9 (-527.4 – -444.8)	495.9 (300.1 – 595.4)

Table S1: Estimates for reductions in infection and mortality levels taken at the end of 2021 for central vaccine sharing strategies relative to the current scenario. Bracketed values represent a 95% prediction interval.

Region	Strategy	Proportion vaccinated (2 doses)	Proportion infected		Mortalities per 100,000	
			unchanged behaviour	adapted behaviour	unchanged behaviour	adapted behaviour
World	Default	44.4%	48.4% (47.4% - 49.6%)	48.4% (47.4% - 49.6%)	133.1 (128.2 - 138.3)	133.1 (128.2 - 138.3)
	2 dose threshold	49.6%	48.3% (47.3% - 49.6%)	48.3% (47.3% - 49.5%)	132.7 (127.8 - 137.9)	132.7 (127.8 - 137.9)
	40+ threshold	49.6%	49.3% (48.2% - 50.6%)	46.8% (45.8% - 47.9%)	131.6 (126.8 - 136.5)	129.5 (124.7 - 134.4)
	65+ threshold	49.4%	47.5% (46.1% - 49.1%)	41.8% (40.3% - 43.3%)	121.6 (117.7 - 126.5)	115.8 (111.9 - 121.0)
	Full sharing	49.4%	44.4% (42.0% - 46.7%)	29.1% (27.6% - 31.0%)	115.4 (112.5 - 119.3)	84.0 (80.3 - 88.9)
Low income	Default	1.7%	64.6% (61.6% - 66.9%)	64.6% (61.6% - 66.9%)	64.0 (59.4 - 70.1)	64.0 (59.4 - 70.1)
	2 dose threshold	11.7%	64.3% (61.3% - 66.6%)	64.3% (61.3% - 66.6%)	63.4 (58.8 - 69.3)	63.4 (58.8 - 69.4)
	40+ threshold	26.9%	62.5% (59.4% - 64.8%)	62.3% (59.3% - 64.6%)	58.3 (53.9 - 63.9)	58.3 (54.0 - 64.2)
	65+ threshold	38.7%	54.7% (51.5% - 57.2%)	48.8% (46.1% - 51.2%)	46.5 (43.4 - 49.8)	44.4 (41.6 - 47.5)
	Full sharing	40.7%	47.8% (45.2% - 50.7%)	38.2% (33.9% - 43.0%)	39.1 (36.5 - 41.5)	31.5 (28.9 - 34.2)
Lower middle income	Default	18.4%	65.4% (63.2% - 68.1%)	65.4% (63.2% - 68.1%)	155.8 (144.2 - 169.7)	155.8 (144.2 - 169.7)
	2 dose threshold	28.5%	65.2% (63.1% - 67.9%)	65.2% (63.1% - 67.9%)	155.2 (143.6 - 169.1)	155.2 (143.6 - 169.1)
	40+ threshold	39.4%	64.2% (61.9% - 66.9%)	64.1% (61.8% - 66.7%)	151.5 (139.9 - 165.5)	151.5 (139.8 - 165.6)
	65+ threshold	46.2%	60.8% (58.2% - 63.6%)	59.3% (56.8% - 62.3%)	134.6 (124.1 - 148.0)	133.1 (122.8 - 145.8)
	Full sharing	47.4%	57.1% (54.0% - 60.4%)	39.8% (37.5% - 42.7%)	117.1 (107.9 - 128.9)	84.8 (75.5 - 95.3)
Higher middle income	Default	66.0%	33.8% (32.5% - 34.7%)	33.8% (32.5% - 34.7%)	105.9 (100.3 - 113.0)	105.9 (100.3 - 113.0)
	2 dose threshold	66.1%	33.8% (32.5% - 34.7%)	33.8% (32.5% - 34.7%)	105.7 (100.1 - 112.8)	105.7 (100.1 - 112.8)
	40+ threshold	56.6%	33.5% (32.1% - 34.5%)	33.4% (32.1% - 34.4%)	103.4 (98.1 - 110.0)	103.4 (98.1 - 109.8)
	65+ threshold	51.6%	31.7% (29.9% - 32.9%)	27.7% (26.3% - 29.0%)	93.4 (89.0 - 98.5)	89.7 (85.3 - 94.0)
	Full sharing	51.4%	28.6% (25.6% - 31.0%)	20.4% (18.3% - 22.3%)	86.0 (81.8 - 90.0)	70.7 (65.7 - 75.9)
High income	Default	75.3%	35.7% (34.5% - 36.7%)	35.7% (34.5% - 36.7%)	162.9 (155.7 - 168.9)	162.9 (155.7 - 168.9)
	2 dose threshold	78.8%	35.6% (34.4% - 36.6%)	35.3% (34.1% - 36.3%)	162.9 (155.7 - 168.9)	162.7 (155.6 - 168.7)
	40+ threshold	68.5%	45.7% (44.5% - 47.0%)	30.7% (30.0% - 31.4%)	171.3 (164.1 - 177.6)	159.8 (152.3 - 165.6)
	65+ threshold	57.3%	50.9% (49.8% - 52.0%)	31.2% (29.9% - 32.6%)	177.8 (170.2 - 184.8)	157.2 (150.3 - 163.1)
	Full sharing	53.6%	50.8% (48.5% - 52.4%)	20.3% (18.4% - 24.3%)	199.1 (190.4 - 208.0)	127.0 (119.8 - 141.7)

Table S2: Estimates for vaccination coverage, proportion infected and mortality rates (per 100,000) under each vaccine sharing strategy and income group taken at the start of 2022. Bracketed values represent a 95% prediction interval, proportion vaccinated is deterministic and so an interval is not provided.

Table S3: Individual country level estimates for vaccination coverage, proportion infected and mortality rates (per 100,000) under each vaccine sharing strategy taken at the start of 2022. Bracketed values represent a 95% prediction interval, proportion vaccinated is deterministic and so an interval is not provided.

Countries excluded due to data constraints:
Vatican City, Federated States of Micronesia, Marshall Islands, Northern Mariana Islands, United States Virgin Islands, Guam, American Samoa, South Georgia and the South Sandwich Islands, British Indian Ocean Territory, Saint Helena, Pitcairn Islands, Anguilla, Falkland Islands, Cayman Islands, Bermuda, British Virgin Islands, Turks and Caicos Islands, Montserrat, Jersey, Guernsey, Isle of Man, Taiwan, Eswatini, Somalia, Somaliland, Seychelles, San Marino, Saint Kitts and Nevis, Palau, Niue, Cook Islands, Aruba, Curacao, Nauru, Western Sahara, Monaco, Liechtenstein, Lebanon, Kosovo, Kiribati, Haiti, Grenada, Saint Pierre and Miquelon, Wallis and Futuna, Saint Martin, Saint-Bartholemy, French Polynesia, New Caledonia, French Southern and Antarctic Lands, Ã. . . land Islands, Dominica, Greenland, Faroe Islands, Turkish Republic of Northern Cyprus, Ivory Coast, Republic of the Congo, Macau, Australian Indian Ocean Territories, Heard Island and McDonald Islands, Norfolk Island, Ashmore and Cartier Islands, Antigua and Barbuda, Andorra, Siachen Glacier, Antarctica, Sint Maarten.

**Country**	**Income bracket**	**Strategy**	**Proportion vaccinated**	**Proportion infected**		**Mortalities per 100,000**	
				unchanged	adapted	unchanged	adapted
Afghanistan	Low	Default	3.7%	93.8% (91.3% - 96.7%)	93.8% (91.3% - 96.7%)	141 (106,207)	141 (106,207)
Afghanistan	Low	2 dose threshold	16.9%	93.8% (91.2% - 96.6%)	93.8% (91.2% - 96.6%)	141 (105,206)	141 (105,206)
Afghanistan	Low	40+ threshold	36.2%	92.8% (90.6% - 96.4%)	92.7% (90.1% - 96.4%)	133 (103,195)	133 (103,195)
Afghanistan	Low	65+ threshold	52.0%	85.9% (80.3% - 93.2%)	73.7% (63.4% - 80.4%)	119 (98,153)	114 (92,140)
Afghanistan	Low	Full sharing	54.5%	79.5% (72.8% - 90.3%)	72.4% (58.7% - 80.4%)	115 (95,144)	113 (92,136)
Albania	Upper middle	Default	29.3%	72.2% (64.0% - 82.0%)	72.2% (64.0% - 82.0%)	314 (233,423)	314 (233,423)
Albania	Upper middle	2 dose threshold	39.0%	72.1% (63.9% - 81.7%)	72.0% (63.9% - 81.7%)	314 (232,423)	314 (232,423)
Albania	Upper middle	40+ threshold	52.8%	71.4% (63.6% - 80.3%)	70.7% (62.8% - 79.0%)	313 (232,421)	313 (232,421)
Albania	Upper middle	65+ threshold	55.1%	70.2% (62.3% - 78.0%)	69.1% (61.5% - 74.6%)	308 (228,417)	308 (227,417)
Albania	Upper middle	Full sharing	52.8%	69.1% (61.2% - 76.1%)	60.1% (54.2% - 66.0%)	286 (214,384)	246 (186,326)
Algeria	Lower middle	Default	10.1%	27.3% (23.9% - 31.9%)	27.3% (23.9% - 31.9%)	90 (78,103)	90 (78,103)
Algeria	Lower middle	2 dose threshold	22.8%	27.2% (23.8% - 31.8%)	27.2% (23.8% - 31.8%)	89 (78,102)	89 (78,102)
Algeria	Lower middle	40+ threshold	40.8%	26.4% (23.0% - 30.9%)	26.4% (23.1% - 31.1%)	84 (73,96)	84 (74,96)
Algeria	Lower middle	65+ threshold	52.4%	20.9% (17.9% - 24.9%)	20.6% (16.4% - 25.5%)	65 (57,75)	64 (56,74)
Algeria	Lower middle	Full sharing	53.7%	16.7% (14.2% - 20.2%)	17.5% (11.7% - 28.1%)	56 (50,64)	57 (43,84)
Angola	Lower middle	Default	6.3%	55.5% (46.6% - 64.9%)	55.5% (46.6% - 64.9%)	83 (66,101)	83 (66,101)
Angola	Lower middle	2 dose threshold	19.2%	55.0% (46.1% - 64.3%)	55.0% (46.2% - 64.2%)	82 (65,100)	82 (65,100)
Angola	Lower middle	40+ threshold	38.1%	53.0% (44.7% - 61.8%)	52.8% (44.5% - 61.4%)	78 (62,95)	78 (62,95)
Angola	Lower middle	65+ threshold	52.1%	45.5% (37.4% - 54.9%)	45.4% (36.1% - 55.1%)	57 (47,68)	55 (45,65)
Angola	Lower middle	Full sharing	54.7%	35.8% (27.7% - 47.6%)	25.9% (17.4% - 44.9%)	43 (36,51)	33 (26,46)
Argentina	Upper middle	Default	61.2%	69.5% (66.1% - 72.4%)	69.5% (66.1% - 72.4%)	245 (214,289)	245 (214,289)
Argentina	Upper middle	2 dose threshold	67.6%	69.5% (66.1% - 72.4%)	69.5% (66.1% - 72.4%)	244 (214,288)	244 (214,288)
Argentina	Upper middle	40+ threshold	57.9%	69.1% (65.8% - 72.0%)	69.1% (65.8% - 72.0%)	242 (212,285)	242 (212,286)
Argentina	Upper middle	65+ threshold	55.2%	66.9% (63.2% - 70.4%)	64.7% (59.8% - 68.7%)	232 (204,272)	228 (202,266)

Country	Income bracket	Strategy	Proportion vaccinated	Proportion infected		Mortalities per 100,000	
				unchanged	adapted	unchanged	adapted
Argentina	Upper middle	Full sharing	53.3%	62.0% (56.8% - 67.2%)	48.2% (36.7% - 59.7%)	226 (197,263)	191 (158,244)
Armenia	Upper middle	Default	3.4%	79.8% (73.1% - 85.6%)	79.8% (73.1% - 85.6%)	398 (306,480)	398 (306,480)
Armenia	Upper middle	2 dose threshold	16.1%	79.7% (73.0% - 85.6%)	79.7% (73.0% - 85.6%)	397 (304,478)	397 (304,478)
Armenia	Upper middle	40+ threshold	35.8%	79.3% (72.5% - 85.0%)	79.3% (72.6% - 84.9%)	385 (287,470)	385 (287,469)
Armenia	Upper middle	65+ threshold	50.4%	75.9% (69.9% - 81.3%)	74.2% (68.3% - 80.3%)	322 (239,406)	318 (237,398)
Armenia	Upper middle	Full sharing	53.2%	73.6% (66.7% - 82.0%)	58.7% (53.9% - 65.4%)	293 (234,355)	269 (223,317)
Australia	High	Default	37.4%	13.4% (11.0% - 16.1%)	13.4% (11.0% - 16.1%)	13 (12,13)	13 (12,13)
Australia	High	2 dose threshold	46.6%	9.7% (7.9% - 11.4%)	9.6% (7.9% - 11.5%)	13 (12,13)	13 (12,13)
Australia	High	40+ threshold	58.1%	4.8% (3.9% - 5.7%)	2.4% (2.1% - 2.6%)	11 (11,12)	11 (11,12)
Australia	High	65+ threshold	54.8%	2.4% (1.9% - 2.9%)	0.6% (0.5% - 0.7%)	8 (8,9)	7 (7,7)
Australia	High	Full sharing	53.2%	1.1% (0.7% - 1.4%)	0.4% (0.4% - 0.7%)	7 (6,7)	4 (4,5)
Austria	High	Default	77.0%	30.1% (26.2% - 34.4%)	30.1% (26.2% - 34.4%)	188 (159,218)	188 (159,218)
Austria	High	2 dose threshold	80.7%	29.9% (25.7% - 34.2%)	29.9% (25.8% - 34.2%)	187 (159,218)	187 (159,218)
Austria	High	40+ threshold	70.7%	31.9% (27.8% - 36.3%)	27.9% (23.8% - 31.6%)	187 (159,218)	187 (158,217)
Austria	High	65+ threshold	58.3%	53.6% (48.9% - 58.1%)	28.3% (22.8% - 35.6%)	197 (167,231)	186 (158,216)
Austria	High	Full sharing	52.7%	52.7% (44.4% - 58.7%)	17.3% (14.6% - 22.4%)	217 (186,256)	138 (115,167)
Azerbaijan	Upper middle	Default	36.5%	77.9% (68.4% - 84.7%)	77.9% (68.4% - 84.7%)	269 (198,350)	269 (198,350)
Azerbaijan	Upper middle	2 dose threshold	45.5%	77.8% (68.3% - 84.6%)	77.8% (68.3% - 84.5%)	269 (198,350)	269 (198,350)
Azerbaijan	Upper middle	40+ threshold	53.3%	77.0% (67.4% - 83.9%)	76.4% (67.2% - 82.7%)	266 (196,345)	265 (196,345)
Azerbaijan	Upper middle	65+ threshold	52.7%	76.0% (66.2% - 83.2%)	62.2% (53.1% - 69.4%)	261 (195,335)	247 (188,316)
Azerbaijan	Upper middle	Full sharing	53.2%	73.7% (59.4% - 82.7%)	53.0% (45.6% - 62.5%)	256 (195,321)	216 (153,295)
Bahrain	High	Default	100.0%	52.7% (46.7% - 58.1%)	52.7% (46.7% - 58.1%)	92 (72,117)	92 (72,117)
Bahrain	High	2 dose threshold	93.2%	52.6% (46.7% - 58.1%)	52.6% (46.7% - 58.1%)	92 (72,117)	92 (72,117)
Bahrain	High	40+ threshold	56.0%	62.6% (58.6% - 66.3%)	57.9% (47.9% - 64.0%)	106 (82,139)	99 (78,128)
Bahrain	High	65+ threshold	61.7%	61.4% (57.0% - 65.3%)	55.7% (49.1% - 60.5%)	114 (88,151)	102 (79,132)
Bahrain	High	Full sharing	63.3%	54.4% (48.9% - 59.4%)	30.5% (23.6% - 40.7%)	101 (79,132)	72 (55,94)
Bangladesh	Lower middle	Default	5.9%	79.1% (70.7% - 91.3%)	79.1% (70.7% - 91.3%)	71 (48,112)	71 (48,112)
Bangladesh	Lower middle	2 dose threshold	18.5%	79.0% (70.7% - 91.3%)	79.0% (70.7% - 91.3%)	70 (48,112)	70 (48,112)
Bangladesh	Lower middle	40+ threshold	37.7%	78.6% (70.0% - 91.0%)	78.6% (69.9% - 91.0%)	69 (47,108)	69 (47,108)
Bangladesh	Lower middle	65+ threshold	52.8%	75.5% (64.2% - 87.6%)	71.0% (54.8% - 80.2%)	62 (43,94)	61 (39,88)
Bangladesh	Lower middle	Full sharing	53.6%	72.0% (54.9% - 84.2%)	48.8% (34.8% - 58.5%)	59 (42,83)	48 (38,66)
Barbados	High	Default	48.4%	25.9% (1.1% - 45.3%)	25.9% (1.1% - 45.3%)	45 (8,72)	45 (8,72)
Barbados	High	2 dose threshold	55.5%	25.8% (1.1% - 45.3%)	25.8% (1.1% - 45.1%)	45 (8,72)	45 (8,72)
Barbados	High	40+ threshold	63.7%	21.6% (1.1% - 37.7%)	21.3% (1.1% - 37.2%)	41 (8,66)	41 (8,66)
Barbados	High	65+ threshold	57.3%	49.1% (1.1% - 81.5%)	5.7% (1.1% - 9.3%)	69 (8,118)	32 (8,49)

Country	Income bracket	Strategy	Proportion vaccinated	Proportion infected		Mortalities per 100,000	
				unchanged	adapted	unchanged	adapted
Barbados	High	Full sharing	52.9%	48.2% (1.1% - 86.4%)	1.6% (1.1% - 2.1%)	71 (8,129)	9 (8,9)
Belarus	Upper middle	Default	15.6%	61.3% (52.2% - 68.6%)	61.3% (52.2% - 68.6%)	514 (380,671)	514 (380,671)
Belarus	Upper middle	2 dose threshold	27.0%	61.2% (52.1% - 68.5%)	61.2% (52.1% - 68.5%)	513 (379,670)	513 (379,670)
Belarus	Upper middle	40+ threshold	45.0%	60.7% (51.4% - 68.1%)	60.4% (51.1% - 67.6%)	494 (366,646)	494 (365,646)
Belarus	Upper middle	65+ threshold	51.5%	56.2% (46.3% - 64.2%)	55.9% (45.4% - 64.5%)	418 (315,533)	412 (308,535)
Belarus	Upper middle	Full sharing	52.8%	52.1% (37.9% - 63.4%)	31.3% (20.4% - 47.0%)	362 (277,462)	249 (195,328)
Belgium	High	Default	93.3%	42.0% (37.8% - 46.1%)	42.0% (37.8% - 46.1%)	405 (348,461)	405 (348,461)
Belgium	High	2 dose threshold	91.8%	41.9% (37.7% - 46.0%)	41.8% (37.5% - 45.8%)	405 (348,461)	405 (348,461)
Belgium	High	40+ threshold	70.4%	46.3% (41.0% - 50.9%)	40.6% (36.5% - 44.6%)	404 (348,461)	404 (348,460)
Belgium	High	65+ threshold	58.1%	62.9% (58.5% - 67.0%)	46.4% (35.2% - 53.7%)	409 (351,468)	406 (348,466)
Belgium	High	Full sharing	52.8%	64.1% (56.4% - 69.6%)	30.6% (23.6% - 43.7%)	465 (397,537)	376 (300,519)
Benin	Lower middle	Default	0.6%	42.9% (35.8% - 50.1%)	42.9% (35.8% - 50.1%)	70 (53,91)	70 (53,91)
Benin	Lower middle	2 dose threshold	14.1%	42.8% (35.7% - 49.9%)	42.8% (35.6% - 49.9%)	69 (53,90)	69 (53,90)
Benin	Lower middle	40+ threshold	34.5%	40.7% (33.8% - 47.4%)	40.6% (33.8% - 46.7%)	55 (41,71)	55 (41,71)
Benin	Lower middle	65+ threshold	51.7%	22.9% (17.7% - 29.9%)	20.7% (14.7% - 28.1%)	33 (28,39)	32 (28,37)
Benin	Lower middle	Full sharing	54.4%	20.6% (14.8% - 27.9%)	8.3% (5.7% - 11.7%)	28 (24,33)	12 (10,15)
Bolivia	Lower middle	Default	29.4%	88.2% (77.8% - 93.8%)	88.2% (77.8% - 93.8%)	771 (482,1141)	771 (482,1141)
Bolivia	Lower middle	2 dose threshold	39.5%	88.2% (77.8% - 93.8%)	88.2% (77.8% - 93.8%)	769 (481,1138)	769 (481,1138)
Bolivia	Lower middle	40+ threshold	49.2%	87.9% (77.6% - 93.6%)	87.9% (77.6% - 93.6%)	755 (475,1123)	755 (475,1123)
Bolivia	Lower middle	65+ threshold	52.5%	85.7% (75.2% - 92.0%)	81.0% (74.1% - 88.1%)	664 (451,987)	650 (430,979)
Bolivia	Lower middle	Full sharing	53.7%	82.1% (70.8% - 89.8%)	69.6% (63.9% - 78.9%)	605 (445,854)	546 (422,752)
Bosnia and Herzegovina	Upper middle	Default	13.2%	77.7% (72.4% - 82.0%)	77.7% (72.4% - 82.0%)	290 (228,374)	290 (228,374)
Bosnia and Herzegovina	Upper middle	2 dose threshold	24.7%	77.5% (72.2% - 81.9%)	77.4% (72.1% - 81.8%)	289 (228,374)	289 (228,374)
Bosnia and Herzegovina	Upper middle	40+ threshold	42.7%	77.2% (71.7% - 81.8%)	75.0% (69.8% - 79.7%)	285 (226,365)	284 (226,364)
Bosnia and Herzegovina	Upper middle	65+ threshold	51.6%	72.1% (65.3% - 78.6%)	66.1% (59.0% - 74.4%)	273 (218,344)	271 (218,339)
Bosnia and Herzegovina	Upper middle	Full sharing	52.7%	68.2% (59.9% - 76.3%)	55.5% (47.0% - 61.1%)	250 (201,312)	219 (168,284)
Brazil	Upper middle	Default	55.6%	64.3% (60.2% - 68.2%)	64.3% (60.2% - 68.2%)	212 (194,230)	212 (194,230)
Brazil	Upper middle	2 dose threshold	62.5%	64.3% (60.1% - 68.2%)	64.3% (60.1% - 68.2%)	212 (194,230)	212 (194,230)
Brazil	Upper middle	40+ threshold	57.2%	63.9% (59.5% - 67.8%)	63.9% (59.5% - 67.7%)	211 (193,228)	211 (193,228)
Brazil	Upper middle	65+ threshold	53.9%	62.7% (58.1% - 66.7%)	60.0% (55.9% - 63.7%)	208 (190,224)	203 (186,221)
Brazil	Upper middle	Full sharing	53.2%	57.7% (51.6% - 63.4%)	46.0% (34.3% - 55.5%)	205 (188,220)	168 (142,207)
Bulgaria	Upper middle	Default	19.9%	79.5% (70.3% - 87.4%)	79.5% (70.3% - 87.4%)	447 (337,595)	447 (337,595)
Bulgaria	Upper middle	2 dose threshold	30.6%	79.5% (70.3% - 87.4%)	79.4% (70.3% - 87.4%)	447 (336,594)	447 (336,594)
Bulgaria	Upper middle	40+ threshold	47.2%	79.2% (69.7% - 87.4%)	78.2% (68.9% - 86.4%)	439 (332,580)	438 (332,579)
Bulgaria	Upper middle	65+ threshold	55.4%	75.7% (64.5% - 84.3%)	59.4% (50.7% - 67.2%)	421 (323,549)	408 (314,533)

Country	Income bracket	Strategy	Proportion vaccinated	Proportion infected		Mortalities per 100,000	
				unchanged	adapted	unchanged	adapted
Bulgaria	Upper middle	Full sharing	52.7%	72.8% (58.7% - 83.4%)	50.1% (33.7% - 59.5%)	405 (311,526)	350 (282,420)
Burkina Faso	Low	Default	0.3%	76.7% (73.0% - 79.1%)	76.7% (73.0% - 79.1%)	35 (24,50)	35 (24,50)
Burkina Faso	Low	2 dose threshold	13.8%	74.9% (70.4% - 79.1%)	74.9% (70.3% - 79.1%)	34 (23,48)	34 (23,47)
Burkina Faso	Low	40+ threshold	34.4%	68.5% (60.1% - 78.9%)	67.3% (58.3% - 78.8%)	31 (23,42)	31 (23,42)
Burkina Faso	Low	65+ threshold	51.8%	62.9% (52.4% - 75.7%)	62.6% (52.3% - 75.8%)	29 (19,39)	29 (19,39)
Burkina Faso	Low	Full sharing	54.6%	60.9% (51.4% - 70.3%)	56.9% (41.4% - 65.3%)	25 (17,35)	23 (16,29)
Cabo Verde	Lower middle	Default	0.0%	67.2% (59.1% - 75.3%)	67.2% (59.1% - 75.3%)	106 (80,133)	106 (80,133)
Cabo Verde	Lower middle	2 dose threshold	13.8%	67.1% (59.1% - 75.3%)	67.1% (59.1% - 75.3%)	105 (79,132)	105 (79,132)
Cabo Verde	Lower middle	40+ threshold	34.1%	66.6% (58.5% - 75.0%)	66.6% (58.6% - 75.0%)	100 (76,123)	100 (76,124)
Cabo Verde	Lower middle	65+ threshold	51.1%	63.0% (53.9% - 72.9%)	62.3% (52.7% - 72.2%)	80 (63,98)	80 (62,96)
Cabo Verde	Lower middle	Full sharing	53.7%	58.0% (48.4% - 69.2%)	49.2% (37.7% - 55.9%)	64 (51,78)	55 (42,67)
Cameroon	Lower middle	Default	0.8%	62.0% (53.0% - 68.7%)	62.0% (53.0% - 68.7%)	58 (41,81)	58 (41,81)
Cameroon	Lower middle	2 dose threshold	14.8%	61.7% (52.6% - 68.4%)	61.7% (52.6% - 68.4%)	57 (41,80)	57 (41,80)
Cameroon	Lower middle	40+ threshold	35.0%	58.9% (50.4% - 65.9%)	58.6% (50.2% - 65.8%)	52 (38,72)	52 (38,72)
Cameroon	Lower middle	65+ threshold	51.9%	52.6% (43.9% - 60.5%)	52.5% (44.6% - 59.9%)	45 (33,62)	45 (33,62)
Cameroon	Lower middle	Full sharing	54.5%	50.0% (41.0% - 57.4%)	47.9% (31.2% - 55.8%)	38 (28,51)	35 (27,43)
Canada	High	Default	99.9%	18.4% (17.0% - 20.2%)	18.4% (17.0% - 20.2%)	164 (148,182)	164 (148,182)
Canada	High	2 dose threshold	93.1%	18.4% (17.0% - 20.2%)	18.4% (17.0% - 20.2%)	164 (148,182)	164 (148,182)
Canada	High	40+ threshold	69.2%	18.4% (17.0% - 20.2%)	18.3% (16.9% - 20.0%)	163 (147,181)	163 (147,181)
Canada	High	65+ threshold	57.1%	26.6% (21.3% - 34.4%)	23.4% (17.1% - 30.5%)	174 (157,192)	168 (143,185)
Canada	High	Full sharing	53.4%	26.4% (19.6% - 36.5%)	8.4% (6.0% - 12.6%)	183 (163,207)	97 (80,120)
Central African Republic	Low	Default	2.5%	74.3% (60.9% - 84.5%)	74.3% (60.9% - 84.5%)	179 (126,236)	179 (126,236)
Central African Republic	Low	2 dose threshold	15.9%	74.3% (60.8% - 84.5%)	74.3% (60.8% - 84.5%)	178 (125,234)	178 (125,234)
Central African Republic	Low	40+ threshold	35.9%	74.3% (60.7% - 84.4%)	74.3% (60.7% - 84.4%)	175 (123,231)	175 (123,231)
Central African Republic	Low	65+ threshold	51.8%	73.6% (59.5% - 83.8%)	73.4% (59.0% - 83.7%)	155 (109,204)	155 (108,204)
Central African Republic	Low	Full sharing	54.7%	71.1% (56.3% - 81.5%)	50.2% (45.1% - 54.2%)	119 (84,155)	88 (60,125)
Chad	Low	Default	0.1%	39.4% (31.6% - 46.0%)	39.4% (31.6% - 46.0%)	57 (46,70)	57 (46,70)
Chad	Low	2 dose threshold	14.5%	39.3% (31.5% - 46.0%)	39.3% (31.5% - 46.0%)	57 (46,69)	57 (46,69)
Chad	Low	40+ threshold	35.5%	39.1% (31.1% - 45.8%)	39.1% (31.1% - 45.8%)	56 (46,68)	56 (46,68)
Chad	Low	65+ threshold	52.2%	38.9% (30.7% - 45.6%)	38.9% (30.7% - 45.6%)	54 (44,65)	54 (44,65)
Chad	Low	Full sharing	55.0%	38.2% (30.1% - 44.9%)	27.8% (18.7% - 42.6%)	47 (38,56)	34 (29,44)
Chile	High	Default	100.0%	42.5% (39.8% - 44.9%)	42.5% (39.8% - 44.9%)	176 (168,182)	176 (168,182)
Chile	High	2 dose threshold	91.1%	42.4% (39.8% - 44.8%)	42.5% (39.8% - 44.8%)	176 (168,182)	176 (168,182)
Chile	High	40+ threshold	64.4%	52.6% (50.2% - 55.5%)	43.7% (41.1% - 46.7%)	184 (176,191)	176 (169,182)
Chile	High	65+ threshold	56.4%	75.9% (73.9% - 77.6%)	50.5% (46.1% - 55.4%)	233 (219,248)	189 (185,194)

Country	Income bracket	Strategy	Proportion vaccinated	Proportion infected		Mortalities per 100,000	
				unchanged	adapted	unchanged	adapted
Chile	High	Full sharing	53.2%	72.8% (67.5% - 77.4%)	30.3% (21.8% - 54.3%)	260 (239,280)	146 (121,231)
China	Upper middle	Default	100.0%	0.3% (0.2% - 0.4%)	0.3% (0.2% - 0.4%)	1 (1,1)	1 (1,1)
China	Upper middle	2 dose threshold	92.2%	0.3% (0.2% - 0.4%)	0.3% (0.2% - 0.4%)	1 (1,1)	1 (1,1)
China	Upper middle	40+ threshold	66.6%	0.3% (0.2% - 0.4%)	0.3% (0.2% - 0.4%)	1 (1,1)	1 (1,1)
China	Upper middle	65+ threshold	53.9%	0.3% (0.2% - 0.4%)	0.3% (0.2% - 0.4%)	1 (1,1)	1 (1,1)
China	Upper middle	Full sharing	53.2%	0.3% (0.2% - 0.4%)	0.2% (0.1% - 0.3%)	1 (1,1)	1 (1,1)
Colombia	Upper middle	Default	36.3%	62.8% (59.5% - 65.4%)	62.8% (59.5% - 65.4%)	291 (254,340)	291 (254,340)
Colombia	Upper middle	2 dose threshold	45.8%	62.8% (59.5% - 65.3%)	62.8% (59.5% - 65.5%)	290 (253,340)	291 (253,340)
Colombia	Upper middle	40+ threshold	52.9%	62.3% (59.0% - 65.0%)	62.4% (59.1% - 65.0%)	285 (247,332)	285 (247,333)
Colombia	Upper middle	65+ threshold	53.1%	57.9% (54.2% - 61.3%)	56.8% (52.8% - 60.3%)	246 (214,285)	242 (213,279)
Colombia	Upper middle	Full sharing	53.3%	50.0% (45.9% - 55.2%)	34.8% (27.4% - 49.3%)	205 (177,236)	155 (133,210)
Costa Rica	Upper middle	Default	50.8%	58.7% (52.9% - 63.8%)	58.7% (52.9% - 63.8%)	95 (89,100)	95 (89,100)
Costa Rica	Upper middle	2 dose threshold	58.2%	58.7% (52.8% - 63.8%)	58.7% (52.8% - 63.8%)	95 (89,100)	95 (89,100)
Costa Rica	Upper middle	40+ threshold	57.3%	58.2% (52.4% - 63.4%)	58.2% (52.5% - 63.3%)	93 (88,99)	93 (88,99)
Costa Rica	Upper middle	65+ threshold	53.9%	55.6% (49.6% - 61.6%)	57.0% (50.1% - 63.4%)	86 (82,90)	85 (81,89)
Costa Rica	Upper middle	Full sharing	53.2%	53.9% (46.3% - 61.2%)	32.6% (24.7% - 48.7%)	83 (79,87)	63 (56,86)
Croatia	High	Default	51.9%	71.2% (66.1% - 75.5%)	71.2% (66.1% - 75.5%)	244 (202,297)	244 (202,297)
Croatia	High	2 dose threshold	58.9%	70.1% (64.8% - 74.4%)	70.0% (64.7% - 74.4%)	244 (202,297)	244 (202,297)
Croatia	High	40+ threshold	67.0%	67.5% (62.0% - 72.1%)	62.3% (57.1% - 67.1%)	243 (201,295)	242 (201,294)
Croatia	High	65+ threshold	57.6%	74.2% (69.7% - 78.0%)	60.3% (52.9% - 65.8%)	239 (198,290)	236 (196,286)
Croatia	High	Full sharing	52.9%	77.5% (70.9% - 81.9%)	47.0% (38.0% - 57.4%)	244 (200,298)	215 (179,265)
Cuba	Upper middle	Default	36.3%	27.7% (23.7% - 31.8%)	27.7% (23.7% - 31.8%)	119 (100,149)	119 (100,149)
Cuba	Upper middle	2 dose threshold	45.7%	27.6% (23.5% - 31.7%)	27.6% (23.6% - 31.7%)	118 (99,147)	118 (99,147)
Cuba	Upper middle	40+ threshold	59.5%	26.5% (22.5% - 30.7%)	26.5% (22.6% - 30.7%)	103 (87,127)	103 (86,127)
Cuba	Upper middle	65+ threshold	50.2%	18.6% (14.5% - 22.4%)	14.9% (10.4% - 19.3%)	45 (39,54)	39 (31,48)
Cuba	Upper middle	Full sharing	52.9%	8.6% (5.8% - 12.4%)	2.7% (0.7% - 5.9%)	20 (17,24)	5 (3,10)
Cyprus	High	Default	56.8%	21.3% (18.0% - 24.3%)	21.3% (18.0% - 24.3%)	37 (35,39)	37 (35,39)
Cyprus	High	2 dose threshold	63.1%	21.3% (18.0% - 24.2%)	21.3% (18.0% - 24.3%)	37 (35,39)	37 (35,39)
Cyprus	High	40+ threshold	64.4%	20.8% (17.6% - 23.6%)	20.8% (17.5% - 23.8%)	36 (35,39)	36 (35,39)
Cyprus	High	65+ threshold	56.0%	18.7% (16.0% - 21.5%)	20.3% (12.6% - 27.1%)	34 (32,36)	34 (30,39)
Cyprus	High	Full sharing	52.9%	15.9% (13.2% - 19.3%)	8.2% (5.0% - 11.0%)	37 (35,39)	18 (14,21)
Czech Republic	High	Default	69.3%	66.0% (57.4% - 73.7%)	66.0% (57.4% - 73.7%)	210 (178,247)	210 (178,247)
Czech Republic	High	2 dose threshold	74.2%	66.0% (57.4% - 73.7%)	66.0% (57.4% - 73.7%)	210 (178,247)	210 (178,247)
Czech Republic	High	40+ threshold	69.3%	66.0% (57.4% - 73.7%)	65.9% (57.2% - 73.7%)	210 (178,247)	210 (178,247)
Czech Republic	High	65+ threshold	57.9%	66.1% (57.7% - 73.7%)	65.8% (57.1% - 73.6%)	209 (177,246)	209 (177,246)

Country	Income bracket	Strategy	Proportion vaccinated	Proportion infected		Mortalities per 100,000	
				unchanged	adapted	unchanged	adapted
Czech Republic	High	Full sharing	52.8%	66.5% (57.9% - 74.2%)	57.7% (40.3% - 64.4%)	222 (186,263)	188 (158,215)
DR Congo	Low	Default	0.1%	65.7% (54.1% - 74.6%)	65.7% (54.1% - 74.6%)	69 (52,92)	69 (52,92)
DR Congo	Low	2 dose threshold	14.3%	65.6% (54.0% - 74.5%)	65.6% (54.0% - 74.5%)	68 (51,91)	68 (51,91)
DR Congo	Low	40+ threshold	34.8%	64.9% (53.0% - 74.0%)	64.9% (53.0% - 73.9%)	62 (47,83)	62 (47,83)
DR Congo	Low	65+ threshold	52.0%	55.6% (42.8% - 66.8%)	48.9% (38.2% - 59.4%)	45 (35,58)	43 (34,56)
DR Congo	Low	Full sharing	54.6%	47.2% (36.1% - 57.4%)	41.1% (24.2% - 56.7%)	38 (29,49)	31 (23,40)
Denmark	High	Default	84.7%	25.3% (21.2% - 30.2%)	25.3% (21.2% - 30.2%)	99 (88,109)	99 (88,109)
Denmark	High	2 dose threshold	87.9%	24.5% (20.7% - 29.3%)	24.4% (20.6% - 29.2%)	99 (88,109)	99 (88,109)
Denmark	High	40+ threshold	69.4%	36.0% (27.2% - 44.7%)	20.0% (17.3% - 22.8%)	98 (88,109)	98 (88,108)
Denmark	High	65+ threshold	58.6%	65.8% (54.6% - 71.6%)	16.0% (13.4% - 18.6%)	103 (91,113)	95 (85,105)
Denmark	High	Full sharing	53.4%	69.6% (55.9% - 74.6%)	9.2% (7.1% - 11.3%)	112 (98,125)	66 (58,75)
Djibouti	Lower middle	Default	3.8%	73.9% (61.8% - 82.8%)	73.9% (61.8% - 82.8%)	210 (148,274)	210 (148,274)
Djibouti	Lower middle	2 dose threshold	17.3%	73.9% (61.8% - 82.8%)	73.9% (61.8% - 82.8%)	209 (147,273)	209 (147,273)
Djibouti	Lower middle	40+ threshold	36.5%	73.8% (61.8% - 82.7%)	73.8% (61.8% - 82.7%)	205 (145,269)	205 (145,269)
Djibouti	Lower middle	65+ threshold	51.0%	73.4% (61.4% - 82.3%)	73.4% (61.4% - 82.2%)	186 (128,246)	185 (128,246)
Djibouti	Lower middle	Full sharing	53.7%	71.5% (59.7% - 80.2%)	44.7% (34.5% - 52.0%)	145 (100,194)	91 (63,135)
Dominican Republic	Upper middle	Default	76.9%	40.0% (33.1% - 46.3%)	40.0% (33.1% - 46.3%)	162 (137,192)	162 (137,192)
Dominican Republic	Upper middle	2 dose threshold	80.9%	40.0% (33.0% - 46.3%)	40.0% (33.0% - 46.3%)	162 (136,192)	162 (136,192)
Dominican Republic	Upper middle	40+ threshold	57.7%	40.2% (33.7% - 46.4%)	40.0% (33.3% - 46.4%)	161 (136,191)	161 (136,191)
Dominican Republic	Upper middle	65+ threshold	54.1%	41.2% (35.5% - 47.4%)	37.2% (29.4% - 44.1%)	158 (133,190)	155 (131,185)
Dominican Republic	Upper middle	Full sharing	53.7%	36.9% (29.8% - 43.9%)	27.2% (18.6% - 42.4%)	136 (114,165)	104 (78,145)
Ecuador	Upper middle	Default	33.2%	78.6% (73.2% - 84.9%)	78.6% (73.2% - 84.9%)	477 (359,639)	477 (359,639)
Ecuador	Upper middle	2 dose threshold	43.2%	78.6% (73.1% - 84.9%)	78.6% (73.2% - 84.9%)	476 (359,638)	476 (358,638)
Ecuador	Upper middle	40+ threshold	50.3%	78.4% (72.7% - 84.7%)	78.4% (72.7% - 84.7%)	473 (356,632)	473 (356,632)
Ecuador	Upper middle	65+ threshold	52.7%	77.1% (70.7% - 83.5%)	76.4% (69.2% - 82.8%)	440 (331,592)	439 (330,589)
Ecuador	Upper middle	Full sharing	53.7%	74.7% (67.0% - 81.0%)	64.1% (60.2% - 66.4%)	396 (303,523)	339 (259,460)
Egypt	Lower middle	Default	6.3%	53.5% (40.8% - 66.1%)	53.5% (40.8% - 66.1%)	159 (120,212)	159 (120,212)
Egypt	Lower middle	2 dose threshold	19.5%	53.5% (40.7% - 66.1%)	53.5% (40.7% - 66.1%)	158 (119,211)	158 (119,211)
Egypt	Lower middle	40+ threshold	38.6%	53.0% (39.9% - 65.9%)	52.9% (39.8% - 65.9%)	154 (117,203)	154 (117,203)
Egypt	Lower middle	65+ threshold	51.5%	49.4% (35.7% - 63.4%)	49.7% (37.5% - 62.2%)	130 (99,168)	130 (98,172)
Egypt	Lower middle	Full sharing	53.9%	44.8% (31.9% - 57.4%)	37.4% (22.9% - 49.6%)	110 (82,144)	93 (74,116)
El Salvador	Lower middle	Default	44.0%	41.5% (36.8% - 46.8%)	41.5% (36.8% - 46.8%)	199 (167,233)	199 (167,233)
El Salvador	Lower middle	2 dose threshold	52.7%	41.5% (36.8% - 46.7%)	41.5% (36.8% - 46.7%)	199 (166,233)	199 (166,233)
El Salvador	Lower middle	40+ threshold	54.4%	40.0% (35.7% - 44.9%)	40.0% (35.6% - 44.8%)	196 (163,229)	196 (164,229)
El Salvador	Lower middle	65+ threshold	52.8%	39.3% (34.7% - 44.6%)	26.8% (23.6% - 30.0%)	192 (159,223)	177 (147,206)

Country	Income bracket	Strategy	Proportion vaccinated	Proportion infected		Mortalities per 100,000	
				unchanged	adapted	unchanged	adapted
El Salvador	Lower middle	Full sharing	53.7%	29.3% (25.3% - 35.7%)	16.7% (13.7% - 20.4%)	159 (133,186)	104 (87,127)
Equatorial Guinea	Upper middle	Default	20.6%	62.0% (53.8% - 70.2%)	62.0% (53.8% - 70.2%)	82 (67,99)	82 (67,99)
Equatorial Guinea	Upper middle	2 dose threshold	32.0%	61.9% (53.7% - 70.2%)	61.9% (53.7% - 70.2%)	82 (66,98)	82 (66,98)
Equatorial Guinea	Upper middle	40+ threshold	45.3%	61.5% (53.1% - 69.8%)	61.5% (53.0% - 69.8%)	80 (65,97)	80 (65,97)
Equatorial Guinea	Upper middle	65+ threshold	51.8%	58.5% (50.3% - 66.9%)	59.0% (49.6% - 67.8%)	70 (55,88)	70 (54,87)
Equatorial Guinea	Upper middle	Full sharing	54.2%	52.4% (46.1% - 57.7%)	46.4% (40.3% - 48.9%)	58 (46,72)	54 (42,67)
Estonia	High	Default	61.8%	42.3% (38.4% - 46.2%)	42.3% (38.4% - 46.2%)	141 (123,167)	141 (123,167)
Estonia	High	2 dose threshold	67.6%	41.9% (37.9% - 45.8%)	41.8% (37.8% - 45.8%)	141 (123,167)	141 (123,167)
Estonia	High	40+ threshold	69.2%	40.7% (36.7% - 44.9%)	38.7% (34.1% - 42.8%)	140 (122,166)	140 (121,165)
Estonia	High	65+ threshold	58.6%	54.7% (51.5% - 57.9%)	48.1% (33.1% - 55.0%)	147 (127,175)	145 (125,173)
Estonia	High	Full sharing	52.9%	58.9% (53.2% - 63.2%)	16.0% (11.6% - 21.6%)	169 (144,202)	69 (57,80)
Eswatini	Lower middle	Default	6.0%	82.5% (76.0% - 86.8%)	82.5% (76.0% - 86.8%)	242 (185,323)	242 (185,323)
Eswatini	Lower middle	2 dose threshold	19.4%	82.5% (75.9% - 86.7%)	82.5% (75.9% - 86.7%)	241 (184,322)	241 (184,322)
Eswatini	Lower middle	40+ threshold	39.1%	81.5% (74.7% - 86.3%)	81.5% (74.9% - 86.3%)	228 (176,300)	228 (176,300)
Eswatini	Lower middle	65+ threshold	51.9%	74.1% (63.4% - 82.7%)	57.9% (47.1% - 74.7%)	198 (152,256)	185 (146,239)
Eswatini	Lower middle	Full sharing	54.6%	62.9% (49.4% - 77.4%)	52.2% (36.9% - 62.1%)	169 (133,216)	154 (122,184)
Finland	High	Default	87.1%	16.1% (13.4% - 19.6%)	16.1% (13.4% - 19.6%)	135 (115,153)	135 (115,153)
Finland	High	2 dose threshold	89.8%	15.6% (13.0% - 18.8%)	15.5% (13.0% - 18.7%)	134 (115,153)	134 (115,153)
Finland	High	40+ threshold	71.3%	34.7% (25.8% - 44.6%)	12.8% (11.2% - 14.9%)	135 (115,154)	134 (114,153)
Finland	High	65+ threshold	59.8%	68.0% (61.7% - 71.6%)	10.1% (8.6% - 12.0%)	168 (141,196)	130 (112,149)
Finland	High	Full sharing	52.7%	74.9% (72.0% - 77.9%)	3.9% (3.4% - 4.5%)	199 (158,234)	65 (55,75)
France	High	Default	78.3%	41.7% (37.2% - 46.1%)	41.7% (37.2% - 46.1%)	224 (189,252)	224 (189,252)
France	High	2 dose threshold	81.9%	41.7% (37.1% - 46.1%)	41.7% (37.1% - 46.1%)	224 (189,252)	224 (189,252)
France	High	40+ threshold	70.8%	41.3% (36.7% - 45.7%)	41.2% (36.7% - 45.6%)	223 (188,252)	223 (188,252)
France	High	65+ threshold	58.8%	44.1% (38.7% - 49.6%)	40.8% (34.6% - 47.0%)	225 (190,254)	221 (188,248)
France	High	Full sharing	52.8%	43.0% (37.2% - 49.3%)	23.1% (18.0% - 40.1%)	248 (207,279)	162 (138,226)
Gabon	Upper middle	Default	3.5%	51.2% (44.0% - 59.2%)	51.2% (44.0% - 59.2%)	89 (69,111)	89 (69,111)
Gabon	Upper middle	2 dose threshold	17.0%	50.9% (43.7% - 58.8%)	50.8% (43.6% - 58.8%)	87 (67,109)	87 (67,109)
Gabon	Upper middle	40+ threshold	36.6%	45.6% (37.8% - 53.0%)	44.7% (37.0% - 51.6%)	67 (51,83)	66 (51,82)
Gabon	Upper middle	65+ threshold	51.3%	32.9% (27.8% - 38.6%)	32.0% (27.3% - 37.6%)	57 (44,71)	57 (44,70)
Gabon	Upper middle	Full sharing	54.1%	31.3% (26.6% - 36.7%)	20.8% (14.0% - 37.4%)	48 (37,60)	32 (24,42)
Gambia	Low	Default	2.7%	75.2% (64.8% - 82.8%)	75.2% (64.8% - 82.8%)	169 (125,235)	169 (125,235)
Gambia	Low	2 dose threshold	16.5%	75.2% (64.7% - 82.7%)	75.2% (64.7% - 82.8%)	168 (124,233)	168 (124,233)
Gambia	Low	40+ threshold	36.6%	74.0% (63.5% - 81.8%)	74.0% (63.2% - 81.8%)	150 (112,206)	150 (112,206)
Gambia	Low	65+ threshold	51.9%	61.2% (50.1% - 71.2%)	50.2% (40.6% - 58.3%)	114 (87,151)	108 (83,144)

Country	Income bracket	Strategy	Proportion vaccinated	Proportion infected		Mortalities per 100,000	
				unchanged	adapted	unchanged	adapted
Gambia	Low	Full sharing	54.5%	51.1% (41.1% - 60.1%)	40.0% (31.0% - 53.1%)	93 (71,123)	73 (54,116)
Georgia	Upper middle	Default	6.2%	88.9% (87.5% - 90.2%)	88.9% (87.5% - 90.2%)	505 (437,587)	505 (437,587)
Georgia	Upper middle	2 dose threshold	19.0%	88.9% (87.5% - 90.1%)	88.9% (87.5% - 90.1%)	505 (436,586)	505 (436,586)
Georgia	Upper middle	40+ threshold	38.2%	88.7% (87.2% - 90.1%)	88.5% (87.1% - 89.8%)	491 (425,568)	491 (425,568)
Georgia	Upper middle	65+ threshold	51.2%	87.6% (85.7% - 89.2%)	83.5% (76.6% - 87.7%)	451 (395,519)	444 (392,513)
Georgia	Upper middle	Full sharing	53.1%	87.3% (84.7% - 89.5%)	71.4% (67.0% - 76.5%)	421 (369,485)	384 (334,452)
Germany	High	Default	79.9%	17.4% (15.8% - 19.1%)	17.4% (15.8% - 19.1%)	124 (118,131)	124 (118,131)
Germany	High	2 dose threshold	83.5%	17.4% (15.7% - 19.0%)	17.4% (15.7% - 19.0%)	124 (118,131)	124 (118,131)
Germany	High	40+ threshold	72.7%	17.6% (15.8% - 19.1%)	17.0% (15.5% - 18.7%)	124 (118,130)	124 (118,130)
Germany	High	65+ threshold	58.5%	35.4% (26.9% - 45.5%)	20.5% (15.3% - 29.1%)	125 (118,133)	125 (117,135)
Germany	High	Full sharing	52.9%	37.4% (29.2% - 44.6%)	11.0% (8.0% - 23.7%)	138 (131,146)	91 (75,156)
Ghana	Lower middle	Default	5.3%	51.5% (43.3% - 59.2%)	51.5% (43.3% - 59.2%)	59 (46,72)	59 (46,72)
Ghana	Lower middle	2 dose threshold	18.7%	49.9% (42.6% - 56.6%)	49.9% (42.5% - 56.4%)	57 (45,71)	57 (45,71)
Ghana	Lower middle	40+ threshold	37.9%	41.1% (35.1% - 46.9%)	39.8% (34.4% - 45.2%)	51 (41,61)	51 (41,61)
Ghana	Lower middle	65+ threshold	52.4%	33.8% (28.0% - 40.0%)	33.4% (27.6% - 39.7%)	48 (40,58)	48 (39,58)
Ghana	Lower middle	Full sharing	54.3%	33.2% (27.4% - 39.4%)	16.4% (12.8% - 19.9%)	43 (35,52)	21 (17,25)
Greece	High	Default	66.7%	27.6% (25.2% - 30.4%)	27.6% (25.2% - 30.4%)	142 (138,146)	142 (138,146)
Greece	High	2 dose threshold	72.0%	26.8% (24.5% - 29.5%)	26.8% (24.5% - 29.4%)	142 (138,146)	142 (138,146)
Greece	High	40+ threshold	70.5%	25.8% (23.3% - 28.7%)	23.0% (20.0% - 25.9%)	140 (136,144)	140 (137,144)
Greece	High	65+ threshold	58.5%	40.0% (31.7% - 47.2%)	27.5% (17.5% - 38.1%)	132 (130,135)	136 (126,147)
Greece	High	Full sharing	52.7%	46.8% (27.8% - 54.6%)	9.5% (6.2% - 14.6%)	158 (151,166)	79 (60,106)
Guatemala	Upper middle	Default	8.1%	69.2% (64.2% - 73.1%)	69.2% (64.2% - 73.1%)	259 (203,331)	259 (203,331)
Guatemala	Upper middle	2 dose threshold	21.1%	69.1% (64.2% - 73.1%)	69.1% (64.2% - 73.1%)	257 (201,329)	257 (201,329)
Guatemala	Upper middle	40+ threshold	39.1%	68.5% (63.5% - 72.5%)	68.5% (63.5% - 72.5%)	242 (190,308)	242 (190,307)
Guatemala	Upper middle	65+ threshold	51.8%	61.5% (55.6% - 66.2%)	59.5% (52.2% - 65.1%)	176 (140,217)	173 (136,214)
Guatemala	Upper middle	Full sharing	54.1%	53.4% (44.5% - 60.9%)	39.5% (32.1% - 50.8%)	140 (111,170)	113 (93,133)
Guinea	Low	Default	6.4%	64.0% (56.2% - 69.9%)	64.0% (56.2% - 69.9%)	50 (36,69)	50 (36,69)
Guinea	Low	2 dose threshold	19.8%	63.9% (56.1% - 69.9%)	63.9% (56.1% - 69.9%)	50 (36,69)	50 (36,69)
Guinea	Low	40+ threshold	38.3%	62.5% (54.8% - 68.8%)	62.5% (54.9% - 68.7%)	46 (33,62)	46 (33,62)
Guinea	Low	65+ threshold	52.0%	50.3% (42.0% - 58.8%)	45.1% (35.0% - 54.8%)	36 (27,47)	35 (27,46)
Guinea	Low	Full sharing	54.6%	43.8% (34.7% - 53.2%)	41.7% (26.7% - 51.4%)	29 (23,38)	27 (21,36)
Guinea-Bissau	Low	Default	2.3%	69.3% (62.5% - 75.1%)	69.3% (62.5% - 75.1%)	81 (59,104)	81 (59,104)
Guinea-Bissau	Low	2 dose threshold	16.2%	69.3% (62.4% - 75.0%)	69.3% (62.4% - 75.0%)	81 (58,104)	81 (58,104)
Guinea-Bissau	Low	40+ threshold	36.1%	68.4% (61.3% - 74.5%)	68.4% (61.4% - 74.5%)	74 (55,94)	74 (55,94)
Guinea-Bissau	Low	65+ threshold	51.9%	58.7% (48.3% - 69.4%)	55.9% (45.2% - 69.2%)	56 (41,70)	55 (41,69)

Country	Income bracket	Strategy	Proportion vaccinated	Proportion infected		Mortalities per 100,000	
				unchanged	adapted	unchanged	adapted
Guinea-Bissau	Low	Full sharing	54.5%	51.6% (43.4% - 61.6%)	48.8% (39.5% - 52.0%)	49 (37,62)	46 (35,62)
Guyana	Upper middle	Default	49.8%	51.5% (41.8% - 61.2%)	51.5% (41.8% - 61.2%)	154 (124,192)	154 (124,192)
Guyana	Upper middle	2 dose threshold	57.2%	51.3% (41.6% - 61.0%)	51.3% (41.6% - 61.0%)	153 (124,191)	153 (124,191)
Guyana	Upper middle	40+ threshold	57.8%	52.2% (42.2% - 62.4%)	49.5% (40.9% - 59.2%)	151 (122,188)	150 (122,188)
Guyana	Upper middle	65+ threshold	53.1%	61.3% (48.4% - 71.6%)	57.7% (43.9% - 67.8%)	148 (121,180)	147 (118,188)
Guyana	Upper middle	Full sharing	53.7%	63.3% (44.5% - 73.5%)	25.3% (15.2% - 38.1%)	127 (106,150)	80 (55,112)
Honduras	Lower middle	Default	14.0%	82.8% (78.9% - 86.2%)	82.8% (78.9% - 86.2%)	314 (240,405)	314 (240,405)
Honduras	Lower middle	2 dose threshold	26.7%	82.8% (78.9% - 86.1%)	82.7% (78.9% - 86.1%)	313 (239,404)	313 (239,404)
Honduras	Lower middle	40+ threshold	40.5%	82.3% (78.4% - 85.7%)	82.2% (78.4% - 85.6%)	297 (228,384)	297 (228,384)
Honduras	Lower middle	65+ threshold	51.6%	78.2% (73.1% - 81.6%)	75.7% (70.2% - 81.1%)	237 (184,302)	234 (183,296)
Honduras	Lower middle	Full sharing	53.9%	74.7% (66.9% - 80.6%)	62.1% (57.4% - 67.7%)	197 (155,248)	168 (131,213)
Hungary	High	Default	80.7%	51.5% (45.9% - 57.3%)	51.5% (45.9% - 57.3%)	232 (202,268)	232 (202,268)
Hungary	High	2 dose threshold	83.7%	51.5% (45.9% - 57.2%)	51.5% (45.9% - 57.2%)	232 (202,268)	232 (202,268)
Hungary	High	40+ threshold	71.4%	52.6% (46.7% - 58.1%)	51.4% (45.9% - 57.0%)	232 (202,268)	232 (202,268)
Hungary	High	65+ threshold	58.4%	68.7% (60.6% - 74.5%)	54.7% (46.9% - 62.8%)	234 (201,269)	232 (200,267)
Hungary	High	Full sharing	52.7%	67.3% (52.9% - 75.4%)	43.1% (31.1% - 52.3%)	257 (221,298)	203 (175,243)
Iceland	High	Default	100.0%	19.7% (12.9% - 25.5%)	19.7% (12.9% - 25.5%)	21 (20,22)	21 (20,22)
Iceland	High	2 dose threshold	93.4%	47.4% (22.6% - 59.0%)	10.1% (7.2% - 13.1%)	21 (20,22)	21 (20,22)
Iceland	High	40+ threshold	66.8%	99.0% (96.5% - 99.9%)	7.7% (6.0% - 10.1%)	28 (22,36)	20 (20,21)
Iceland	High	65+ threshold	57.1%	90.8% (70.1% - 99.4%)	6.3% (4.8% - 7.9%)	43 (34,54)	19 (19,20)
Iceland	High	Full sharing	54.2%	92.3% (70.6% - 99.6%)	3.3% (2.6% - 4.1%)	45 (34,56)	12 (11,12)
India	Lower middle	Default	33.3%	68.6% (64.0% - 72.5%)	68.6% (64.0% - 72.5%)	198 (170,223)	198 (170,223)
India	Lower middle	2 dose threshold	43.3%	68.5% (64.0% - 72.5%)	68.5% (64.0% - 72.5%)	197 (170,223)	197 (170,223)
India	Lower middle	40+ threshold	50.0%	68.4% (63.8% - 72.3%)	68.4% (63.8% - 72.3%)	196 (168,221)	196 (168,221)
India	Lower middle	65+ threshold	52.9%	67.2% (62.8% - 70.9%)	66.7% (62.5% - 70.6%)	179 (154,203)	179 (153,203)
India	Lower middle	Full sharing	53.8%	64.6% (60.6% - 68.1%)	41.9% (38.5% - 46.4%)	157 (134,178)	105 (85,125)
Indonesia	Upper middle	Default	18.8%	70.8% (61.6% - 78.1%)	70.8% (61.6% - 78.1%)	134 (97,184)	134 (97,184)
Indonesia	Upper middle	2 dose threshold	30.6%	70.7% (61.4% - 78.1%)	70.7% (61.5% - 78.1%)	134 (97,183)	134 (97,183)
Indonesia	Upper middle	40+ threshold	44.8%	69.8% (60.1% - 77.2%)	69.8% (59.9% - 77.5%)	128 (94,173)	128 (94,173)
Indonesia	Upper middle	65+ threshold	52.6%	61.8% (49.7% - 71.1%)	34.6% (27.4% - 41.3%)	103 (77,134)	82 (63,106)
Indonesia	Upper middle	Full sharing	53.6%	47.4% (32.9% - 63.0%)	20.6% (14.0% - 38.4%)	83 (65,105)	46 (35,59)
Iran	Upper middle	Default	8.0%	72.4% (66.7% - 76.6%)	72.4% (66.7% - 76.6%)	265 (213,338)	265 (213,338)
Iran	Upper middle	2 dose threshold	20.9%	72.3% (66.7% - 76.5%)	72.3% (66.7% - 76.5%)	264 (212,337)	264 (212,337)
Iran	Upper middle	40+ threshold	39.7%	71.5% (65.8% - 75.8%)	71.4% (65.7% - 75.6%)	253 (204,320)	252 (204,320)
Iran	Upper middle	65+ threshold	51.1%	66.6% (58.9% - 72.0%)	63.4% (54.2% - 70.3%)	206 (169,250)	202 (166,243)

Country	Income bracket	Strategy	Proportion vaccinated	Proportion infected		Mortalities per 100,000	
				unchanged	adapted	unchanged	adapted
Iran	Upper middle	Full sharing	53.5%	62.0% (51.1% - 70.6%)	48.0% (35.7% - 54.1%)	173 (144,208)	146 (121,178)
Iraq	Upper middle	Default	3.2%	82.1% (74.1% - 88.7%)	82.1% (74.1% - 88.7%)	349 (278,449)	349 (278,449)
Iraq	Upper middle	2 dose threshold	17.0%	82.1% (74.1% - 88.7%)	82.1% (74.1% - 88.7%)	346 (276,446)	346 (276,446)
Iraq	Upper middle	40+ threshold	36.4%	81.7% (73.7% - 88.0%)	81.7% (73.7% - 87.9%)	329 (260,424)	329 (260,424)
Iraq	Upper middle	65+ threshold	51.7%	77.2% (69.1% - 83.1%)	74.6% (66.5% - 78.5%)	235 (180,319)	229 (166,312)
Iraq	Upper middle	Full sharing	54.4%	69.4% (61.3% - 77.3%)	51.1% (45.2% - 57.7%)	197 (163,250)	177 (148,213)
Ireland	High	Default	82.6%	37.1% (32.5% - 42.1%)	37.1% (32.5% - 42.1%)	103 (100,105)	103 (100,105)
Ireland	High	2 dose threshold	85.8%	36.0% (31.6% - 40.6%)	35.9% (31.6% - 40.5%)	103 (100,105)	103 (100,105)
Ireland	High	40+ threshold	66.8%	61.1% (55.0% - 69.2%)	20.9% (17.2% - 27.5%)	111 (105,115)	99 (97,102)
Ireland	High	65+ threshold	56.9%	70.5% (60.0% - 79.7%)	19.1% (16.9% - 21.1%)	137 (122,148)	98 (94,102)
Ireland	High	Full sharing	53.2%	71.8% (65.3% - 79.7%)	9.7% (8.0% - 11.4%)	152 (133,164)	57 (54,65)
Israel	High	Default	100.0%	33.4% (30.5% - 36.7%)	33.4% (30.5% - 36.7%)	55 (51,58)	55 (51,58)
Israel	High	2 dose threshold	88.8%	59.2% (52.7% - 64.2%)	35.9% (31.8% - 41.9%)	74 (63,84)	55 (49,59)
Israel	High	40+ threshold	70.9%	64.4% (56.0% - 70.3%)	57.2% (51.3% - 63.0%)	76 (64,86)	69 (62,79)
Israel	High	65+ threshold	68.2%	63.6% (53.5% - 70.5%)	57.6% (52.5% - 63.0%)	77 (63,87)	70 (62,79)
Israel	High	Full sharing	66.1%	56.2% (41.5% - 67.9%)	25.2% (18.8% - 32.1%)	74 (60,88)	50 (46,53)
Italy	High	Default	80.5%	34.0% (30.6% - 36.9%)	34.0% (30.6% - 36.9%)	305 (279,337)	305 (279,337)
Italy	High	2 dose threshold	84.1%	34.0% (30.5% - 36.9%)	34.0% (30.5% - 37.0%)	305 (279,337)	305 (279,337)
Italy	High	40+ threshold	73.2%	33.6% (30.2% - 36.5%)	33.5% (30.1% - 36.4%)	304 (278,336)	304 (278,336)
Italy	High	65+ threshold	59.2%	34.4% (30.8% - 37.4%)	33.5% (29.3% - 37.0%)	303 (278,333)	301 (274,334)
Italy	High	Full sharing	52.7%	34.4% (30.3% - 38.4%)	22.0% (16.5% - 41.3%)	334 (304,372)	242 (199,382)
Jamaica	Upper middle	Default	9.4%	34.3% (29.6% - 39.3%)	34.3% (29.6% - 39.3%)	136 (115,160)	136 (115,160)
Jamaica	Upper middle	2 dose threshold	22.1%	34.2% (29.4% - 39.2%)	34.2% (29.4% - 39.2%)	135 (115,159)	135 (115,159)
Jamaica	Upper middle	40+ threshold	40.7%	32.9% (28.2% - 38.0%)	32.8% (28.0% - 37.8%)	125 (106,147)	125 (106,147)
Jamaica	Upper middle	65+ threshold	52.0%	26.3% (22.0% - 31.2%)	24.6% (19.9% - 29.6%)	97 (82,112)	93 (78,110)
Jamaica	Upper middle	Full sharing	53.4%	18.7% (14.9% - 23.3%)	6.5% (5.0% - 8.5%)	74 (63,86)	30 (26,37)
Japan	High	Default	37.6%	10.3% (8.0% - 12.3%)	10.3% (8.0% - 12.3%)	75 (62,93)	75 (62,93)
Japan	High	2 dose threshold	47.1%	10.2% (7.9% - 12.3%)	10.2% (7.9% - 12.2%)	74 (62,92)	74 (62,92)
Japan	High	40+ threshold	63.1%	9.6% (7.3% - 11.5%)	9.6% (7.4% - 11.5%)	68 (57,84)	68 (57,85)
Japan	High	65+ threshold	54.5%	6.0% (4.4% - 7.3%)	5.6% (4.3% - 6.8%)	43 (36,53)	41 (35,52)
Japan	High	Full sharing	52.5%	4.5% (3.4% - 5.3%)	3.1% (1.8% - 7.4%)	35 (28,43)	21 (15,32)
Jordan	Upper middle	Default	43.8%	69.7% (64.2% - 74.1%)	69.7% (64.2% - 74.1%)	168 (126,225)	168 (126,225)
Jordan	Upper middle	2 dose threshold	52.6%	69.7% (64.2% - 74.1%)	69.7% (64.2% - 74.1%)	167 (126,225)	167 (126,225)
Jordan	Upper middle	40+ threshold	50.5%	69.7% (64.1% - 74.1%)	69.7% (64.1% - 74.1%)	166 (125,222)	166 (125,222)
Jordan	Upper middle	65+ threshold	52.7%	69.3% (63.8% - 73.7%)	69.3% (63.8% - 73.6%)	157 (117,211)	157 (117,211)

Country	Income bracket	Strategy	Proportion vaccinated	Proportion infected		Mortalities per 100,000	
				unchanged	adapted	unchanged	adapted
Jordan	Upper middle	Full sharing	54.2%	68.1% (62.7% - 72.3%)	47.6% (36.9% - 53.6%)	134 (99,181)	89 (67,106)
Kazakhstan	Upper middle	Default	32.3%	63.0% (56.4% - 68.9%)	63.0% (56.4% - 68.9%)	286 (234,354)	286 (234,354)
Kazakhstan	Upper middle	2 dose threshold	42.6%	62.9% (56.3% - 68.9%)	62.9% (56.4% - 68.9%)	285 (233,352)	285 (233,353)
Kazakhstan	Upper middle	40+ threshold	51.4%	62.4% (55.9% - 68.4%)	62.4% (55.9% - 68.3%)	275 (226,341)	275 (225,341)
Kazakhstan	Upper middle	65+ threshold	52.1%	58.0% (50.9% - 64.7%)	51.4% (43.3% - 60.0%)	222 (184,268)	201 (166,241)
Kazakhstan	Upper middle	Full sharing	53.7%	49.6% (40.5% - 61.1%)	27.0% (18.9% - 43.5%)	179 (150,214)	125 (95,223)
Kenya	Lower middle	Default	3.6%	84.0% (80.2% - 87.3%)	84.0% (80.2% - 87.3%)	113 (90,136)	113 (90,136)
Kenya	Lower middle	2 dose threshold	17.3%	84.0% (80.1% - 87.3%)	84.0% (80.1% - 87.3%)	112 (89,135)	112 (89,135)
Kenya	Lower middle	40+ threshold	37.2%	83.3% (79.4% - 86.6%)	83.3% (79.4% - 86.5%)	108 (86,130)	108 (86,130)
Kenya	Lower middle	65+ threshold	52.1%	78.2% (72.8% - 83.2%)	74.6% (67.5% - 80.6%)	93 (74,111)	91 (73,109)
Kenya	Lower middle	Full sharing	54.5%	74.2% (65.3% - 81.4%)	63.4% (55.4% - 71.4%)	76 (61,91)	64 (51,79)
Kuwait	High	Default	71.8%	62.1% (50.5% - 71.8%)	62.1% (50.5% - 71.8%)	62 (45,83)	62 (45,83)
Kuwait	High	2 dose threshold	76.3%	62.1% (50.5% - 71.8%)	62.1% (50.5% - 71.8%)	62 (45,83)	62 (45,83)
Kuwait	High	40+ threshold	60.1%	63.9% (52.0% - 73.0%)	64.5% (54.4% - 73.6%)	63 (45,84)	63 (45,85)
Kuwait	High	65+ threshold	52.4%	72.9% (66.1% - 77.6%)	64.0% (50.6% - 73.0%)	71 (50,101)	66 (48,89)
Kuwait	High	Full sharing	53.7%	66.7% (56.1% - 74.4%)	52.0% (32.7% - 63.2%)	64 (46,88)	53 (40,73)
Kyrgyzstan	Lower middle	Default	5.0%	97.1% (94.5% - 98.3%)	97.1% (94.5% - 98.3%)	207 (151,335)	207 (151,335)
Kyrgyzstan	Lower middle	2 dose threshold	18.6%	97.1% (94.2% - 98.3%)	97.1% (94.1% - 98.3%)	206 (151,332)	206 (151,332)
Kyrgyzstan	Lower middle	40+ threshold	37.3%	96.8% (92.3% - 98.2%)	96.7% (92.1% - 98.2%)	201 (150,305)	201 (150,305)
Kyrgyzstan	Lower middle	65+ threshold	51.3%	95.2% (86.0% - 97.6%)	91.1% (79.9% - 96.0%)	190 (148,251)	188 (147,236)
Kyrgyzstan	Lower middle	Full sharing	53.9%	94.2% (80.1% - 97.3%)	87.8% (79.9% - 95.7%)	184 (147,234)	177 (145,212)
Latvia	High	Default	49.1%	59.8% (53.3% - 65.9%)	59.8% (53.3% - 65.9%)	219 (175,264)	219 (175,264)
Latvia	High	2 dose threshold	56.6%	59.4% (52.8% - 65.6%)	59.3% (52.8% - 65.5%)	219 (175,264)	219 (175,264)
Latvia	High	40+ threshold	65.9%	56.6% (49.7% - 63.3%)	55.0% (48.6% - 62.0%)	215 (173,259)	215 (173,259)
Latvia	High	65+ threshold	56.6%	61.0% (53.7% - 67.2%)	45.6% (36.5% - 53.2%)	213 (172,253)	201 (163,241)
Latvia	High	Full sharing	53.0%	61.4% (51.2% - 68.9%)	20.4% (14.9% - 27.5%)	215 (175,259)	110 (91,140)
Liberia	Low	Default	3.1%	64.9% (58.1% - 69.9%)	64.9% (58.1% - 69.9%)	77 (55,109)	77 (55,109)
Liberia	Low	2 dose threshold	16.9%	64.8% (58.0% - 69.8%)	64.8% (58.1% - 69.9%)	76 (55,108)	76 (55,108)
Liberia	Low	40+ threshold	36.7%	64.1% (57.4% - 69.2%)	64.1% (57.1% - 69.4%)	69 (50,97)	69 (50,97)
Liberia	Low	65+ threshold	51.9%	54.4% (46.4% - 61.4%)	36.9% (27.7% - 46.4%)	42 (32,56)	36 (28,46)
Liberia	Low	Full sharing	54.6%	38.6% (29.9% - 46.0%)	32.6% (21.2% - 46.6%)	33 (26,42)	31 (24,40)
Libya	Upper middle	Default	9.4%	79.2% (70.6% - 85.5%)	79.2% (70.6% - 85.5%)	244 (180,322)	244 (180,322)
Libya	Upper middle	2 dose threshold	22.3%	79.2% (70.5% - 85.5%)	79.2% (70.5% - 85.5%)	243 (179,320)	243 (179,320)
Libya	Upper middle	40+ threshold	40.9%	78.2% (69.4% - 84.5%)	78.2% (69.3% - 84.5%)	223 (165,292)	223 (166,292)
Libya	Upper middle	65+ threshold	51.2%	70.8% (60.0% - 79.1%)	60.1% (49.1% - 70.6%)	173 (131,222)	164 (126,209)

Country	Income bracket	Strategy	Proportion vaccinated	Proportion infected		Mortalities per 100,000	
				unchanged	adapted	unchanged	adapted
Libya	Upper middle	Full sharing	53.9%	64.5% (49.9% - 76.0%)	46.5% (32.6% - 60.4%)	142 (108,182)	107 (85,137)
Lithuania	High	Default	66.8%	71.3% (63.4% - 77.7%)	71.3% (63.4% - 77.7%)	320 (259,389)	320 (259,389)
Lithuania	High	2 dose threshold	71.7%	71.0% (63.1% - 77.4%)	71.0% (63.2% - 77.4%)	320 (259,389)	320 (259,389)
Lithuania	High	40+ threshold	71.5%	71.0% (63.1% - 77.7%)	69.7% (61.4% - 76.4%)	319 (259,388)	318 (258,387)
Lithuania	High	65+ threshold	58.3%	76.6% (70.7% - 81.6%)	62.1% (54.9% - 69.6%)	326 (261,401)	311 (251,378)
Lithuania	High	Full sharing	53.2%	79.3% (73.4% - 84.7%)	47.5% (34.8% - 58.9%)	352 (281,438)	284 (230,350)
Luxembourg	High	Default	80.8%	38.8% (35.3% - 41.9%)	38.8% (35.3% - 41.9%)	324 (287,368)	324 (287,368)
Luxembourg	High	2 dose threshold	84.8%	38.8% (35.3% - 41.9%)	38.8% (35.3% - 41.9%)	324 (287,368)	324 (287,368)
Luxembourg	High	40+ threshold	66.9%	38.5% (35.0% - 41.6%)	38.4% (35.0% - 41.5%)	323 (286,367)	323 (286,367)
Luxembourg	High	65+ threshold	56.3%	43.9% (39.2% - 47.9%)	38.8% (33.0% - 44.2%)	324 (287,367)	320 (284,364)
Luxembourg	High	Full sharing	53.1%	41.0% (34.6% - 46.4%)	35.5% (32.5% - 39.5%)	332 (294,377)	283 (243,343)
Madagascar	Low	Default	1.4%	75.0% (68.0% - 79.5%)	75.0% (68.0% - 79.5%)	77 (55,105)	77 (55,105)
Madagascar	Low	2 dose threshold	15.4%	75.0% (68.0% - 79.5%)	75.0% (68.0% - 79.5%)	77 (54,105)	77 (54,105)
Madagascar	Low	40+ threshold	35.9%	74.6% (67.8% - 79.3%)	74.6% (67.8% - 79.2%)	74 (53,100)	74 (53,101)
Madagascar	Low	65+ threshold	51.9%	72.0% (64.9% - 77.6%)	71.5% (63.9% - 77.4%)	66 (48,87)	65 (48,87)
Madagascar	Low	Full sharing	54.5%	69.0% (60.9% - 76.1%)	64.4% (57.4% - 69.1%)	54 (40,72)	48 (34,64)
Malawi	Low	Default	4.1%	81.4% (75.6% - 87.7%)	81.4% (75.6% - 87.7%)	50 (41,66)	50 (41,66)
Malawi	Low	2 dose threshold	17.8%	80.5% (75.2% - 85.9%)	80.5% (75.1% - 85.8%)	49 (40,65)	49 (40,65)
Malawi	Low	40+ threshold	37.7%	74.0% (67.1% - 79.4%)	72.2% (64.7% - 78.1%)	44 (36,57)	44 (36,57)
Malawi	Low	65+ threshold	52.2%	59.2% (52.6% - 64.6%)	58.3% (52.1% - 63.7%)	42 (34,54)	42 (34,54)
Malawi	Low	Full sharing	54.8%	58.1% (52.0% - 63.5%)	32.2% (23.6% - 52.2%)	37 (30,49)	18 (15,22)
Malaysia	Upper middle	Default	31.3%	57.1% (49.9% - 64.6%)	57.1% (49.9% - 64.6%)	55 (41,72)	55 (41,72)
Malaysia	Upper middle	2 dose threshold	42.0%	57.1% (49.8% - 64.6%)	57.0% (49.8% - 64.6%)	55 (41,71)	55 (41,71)
Malaysia	Upper middle	40+ threshold	47.6%	56.4% (49.1% - 64.2%)	56.3% (49.0% - 64.1%)	50 (37,65)	50 (37,65)
Malaysia	Upper middle	65+ threshold	52.1%	51.4% (42.3% - 60.5%)	45.3% (32.5% - 55.2%)	27 (21,35)	26 (18,37)
Malaysia	Upper middle	Full sharing	53.6%	46.4% (30.9% - 61.3%)	4.0% (2.7% - 5.9%)	20 (15,25)	4 (3,5)
Maldives	Upper middle	Default	90.5%	45.8% (35.5% - 55.4%)	45.8% (35.5% - 55.4%)	47 (37,60)	47 (37,60)
Maldives	Upper middle	2 dose threshold	92.0%	45.7% (35.5% - 55.3%)	45.7% (35.5% - 55.3%)	47 (37,60)	47 (37,60)
Maldives	Upper middle	40+ threshold	48.7%	82.2% (76.9% - 85.6%)	40.9% (25.9% - 56.5%)	67 (47,96)	43 (35,57)
Maldives	Upper middle	65+ threshold	52.3%	92.7% (89.9% - 94.7%)	50.3% (37.1% - 60.9%)	102 (64,163)	48 (37,62)
Maldives	Upper middle	Full sharing	53.3%	89.6% (87.0% - 92.6%)	20.6% (10.9% - 42.8%)	83 (54,131)	23 (19,35)
Mali	Low	Default	1.5%	56.0% (46.5% - 64.4%)	56.0% (46.5% - 64.4%)	91 (67,126)	91 (67,126)
Mali	Low	2 dose threshold	15.6%	55.6% (46.0% - 64.2%)	55.6% (46.0% - 64.1%)	90 (67,125)	90 (67,125)
Mali	Low	40+ threshold	36.1%	54.2% (44.4% - 63.5%)	54.1% (44.3% - 63.5%)	87 (65,119)	87 (65,119)
Mali	Low	65+ threshold	52.3%	51.5% (41.3% - 61.9%)	51.3% (41.3% - 61.4%)	77 (58,103)	77 (58,103)

Country	Income bracket	Strategy	Proportion vaccinated	Proportion infected		Mortalities per 100,000	
				unchanged	adapted	unchanged	adapted
Mali	Low	Full sharing	55.0%	48.8% (38.6% - 59.0%)	45.3% (32.1% - 52.8%)	64 (49,85)	59 (43,74)
Malta	High	Default	100.0%	19.9% (17.8% - 21.6%)	19.9% (17.8% - 21.6%)	155 (138,175)	155 (138,175)
Malta	High	2 dose threshold	94.5%	19.9% (17.8% - 21.6%)	19.9% (17.8% - 21.6%)	155 (138,175)	155 (138,175)
Malta	High	40+ threshold	70.5%	21.3% (18.2% - 25.5%)	20.1% (17.9% - 21.9%)	155 (138,175)	155 (138,175)
Malta	High	65+ threshold	59.4%	66.2% (56.9% - 71.5%)	20.2% (18.1% - 22.0%)	170 (150,191)	154 (137,175)
Malta	High	Full sharing	52.8%	70.6% (58.2% - 77.0%)	8.6% (7.7% - 9.4%)	213 (183,242)	84 (71,96)
Mauritania	Lower middle	Default	6.6%	57.6% (49.6% - 64.7%)	57.6% (49.6% - 64.7%)	140 (105,185)	140 (105,185)
Mauritania	Lower middle	2 dose threshold	20.0%	57.5% (49.5% - 64.6%)	57.5% (49.5% - 64.6%)	139 (104,184)	139 (104,184)
Mauritania	Lower middle	40+ threshold	37.4%	56.6% (48.5% - 63.9%)	56.6% (48.8% - 63.8%)	126 (96,166)	126 (96,166)
Mauritania	Lower middle	65+ threshold	51.9%	45.7% (36.7% - 54.1%)	41.5% (33.0% - 51.9%)	85 (66,110)	82 (64,107)
Mauritania	Lower middle	Full sharing	54.5%	36.1% (28.8% - 44.0%)	35.4% (24.6% - 47.3%)	71 (55,93)	71 (54,100)
Mauritius	High	Default	61.8%	15.7% (13.8% - 18.0%)	15.7% (13.8% - 18.0%)	24 (21,28)	24 (21,28)
Mauritius	High	2 dose threshold	68.8%	15.6% (13.6% - 17.9%)	15.6% (13.7% - 17.8%)	24 (20,28)	24 (20,28)
Mauritius	High	40+ threshold	58.9%	14.2% (12.0% - 16.2%)	14.1% (11.8% - 16.1%)	22 (19,25)	22 (19,25)
Mauritius	High	65+ threshold	55.0%	13.0% (10.6% - 15.5%)	3.1% (1.9% - 4.8%)	21 (18,25)	10 (9,11)
Mauritius	High	Full sharing	53.2%	7.8% (3.7% - 13.2%)	1.0% (0.6% - 1.7%)	16 (11,21)	5 (4,7)
Mexico	Upper middle	Default	41.2%	72.8% (71.3% - 74.6%)	72.8% (71.3% - 74.6%)	368 (310,433)	368 (310,433)
Mexico	Upper middle	2 dose threshold	50.1%	72.8% (71.3% - 74.6%)	72.8% (71.3% - 74.6%)	368 (310,433)	368 (310,433)
Mexico	Upper middle	40+ threshold	54.7%	72.2% (70.6% - 74.0%)	72.1% (70.5% - 73.8%)	365 (308,430)	365 (308,430)
Mexico	Upper middle	65+ threshold	53.8%	71.2% (69.2% - 72.9%)	65.2% (61.6% - 68.7%)	354 (299,417)	346 (294,406)
Mexico	Upper middle	Full sharing	53.8%	67.6% (62.8% - 71.3%)	57.4% (50.1% - 60.4%)	338 (287,396)	306 (248,364)
Moldova	Lower middle	Default	16.7%	80.6% (69.1% - 90.7%)	80.6% (69.1% - 90.7%)	175 (119,252)	175 (119,252)
Moldova	Lower middle	2 dose threshold	28.3%	80.5% (68.9% - 90.7%)	80.5% (68.8% - 90.7%)	175 (119,251)	175 (119,251)
Moldova	Lower middle	40+ threshold	45.9%	80.0% (68.1% - 90.5%)	79.5% (66.5% - 90.5%)	173 (117,249)	173 (117,249)
Moldova	Lower middle	65+ threshold	51.5%	77.8% (61.6% - 88.8%)	76.4% (60.2% - 87.7%)	163 (111,238)	162 (110,238)
Moldova	Lower middle	Full sharing	53.1%	75.3% (59.2% - 88.3%)	62.1% (42.4% - 74.5%)	149 (109,213)	131 (105,167)
Montenegro	Upper middle	Default	35.9%	93.3% (89.4% - 96.7%)	93.3% (89.4% - 96.7%)	243 (182,339)	243 (182,339)
Montenegro	Upper middle	2 dose threshold	45.0%	93.0% (89.0% - 96.4%)	93.0% (89.0% - 96.3%)	243 (182,339)	243 (182,339)
Montenegro	Upper middle	40+ threshold	58.0%	92.4% (88.8% - 95.3%)	90.2% (86.2% - 94.2%)	242 (182,337)	242 (182,336)
Montenegro	Upper middle	65+ threshold	54.1%	91.9% (87.8% - 95.4%)	87.9% (79.8% - 93.6%)	236 (178,331)	235 (178,328)
Montenegro	Upper middle	Full sharing	53.1%	90.8% (85.5% - 95.3%)	81.7% (70.2% - 91.2%)	219 (172,302)	188 (152,260)
Morocco	Lower middle	Default	44.9%	68.1% (59.8% - 76.0%)	68.1% (59.8% - 76.0%)	199 (160,249)	199 (160,249)
Morocco	Lower middle	2 dose threshold	53.0%	68.0% (59.7% - 76.0%)	68.0% (59.8% - 76.0%)	198 (160,248)	198 (160,248)
Morocco	Lower middle	40+ threshold	58.3%	67.5% (59.0% - 75.2%)	67.5% (58.9% - 75.2%)	197 (159,246)	197 (159,246)
Morocco	Lower middle	65+ threshold	56.5%	68.0% (59.9% - 76.2%)	63.0% (52.6% - 69.6%)	200 (160,250)	195 (153,239)

Country	Income bracket	Strategy	Proportion vaccinated	Proportion infected		Mortalities per 100,000	
				unchanged	adapted	unchanged	adapted
Morocco	Lower middle	Full sharing	53.7%	65.3% (54.8% - 73.5%)	42.4% (40.5% - 45.0%)	206 (163,254)	176 (139,232)
Mozambique	Low	Default	2.3%	76.0% (65.8% - 83.6%)	76.0% (65.8% - 83.6%)	106 (82,132)	106 (82,132)
Mozambique	Low	2 dose threshold	16.3%	76.0% (65.7% - 83.6%)	76.0% (65.7% - 83.6%)	105 (82,131)	105 (82,131)
Mozambique	Low	40+ threshold	36.6%	74.8% (64.5% - 82.6%)	74.8% (64.5% - 82.6%)	92 (72,114)	92 (72,114)
Mozambique	Low	65+ threshold	52.2%	56.3% (44.5% - 67.4%)	39.6% (31.0% - 47.6%)	61 (50,71)	56 (46,66)
Mozambique	Low	Full sharing	54.7%	41.8% (32.4% - 51.8%)	17.7% (10.2% - 43.7%)	51 (42,60)	17 (13,24)
Myanmar	Lower middle	Default	8.3%	55.7% (48.4% - 62.8%)	55.7% (48.4% - 62.8%)	142 (113,172)	142 (113,172)
Myanmar	Lower middle	2 dose threshold	21.4%	55.6% (48.2% - 62.7%)	55.6% (48.3% - 62.7%)	141 (112,171)	141 (112,171)
Myanmar	Lower middle	40+ threshold	39.9%	54.3% (47.2% - 61.4%)	54.3% (47.2% - 61.4%)	129 (103,157)	129 (103,155)
Myanmar	Lower middle	65+ threshold	52.4%	41.5% (34.9% - 49.8%)	15.7% (13.1% - 19.1%)	75 (62,89)	48 (40,57)
Myanmar	Lower middle	Full sharing	53.9%	25.0% (18.9% - 34.5%)	13.9% (9.5% - 33.0%)	54 (43,65)	40 (31,74)
Namibia	Upper middle	Default	9.3%	86.3% (82.4% - 90.0%)	86.3% (82.4% - 90.0%)	107 (80,132)	107 (80,132)
Namibia	Upper middle	2 dose threshold	22.2%	86.3% (82.4% - 90.0%)	86.3% (82.4% - 90.0%)	106 (80,131)	106 (80,130)
Namibia	Upper middle	40+ threshold	39.6%	85.9% (81.9% - 89.8%)	85.9% (81.8% - 89.7%)	98 (75,121)	98 (75,121)
Namibia	Upper middle	65+ threshold	51.6%	80.9% (75.6% - 86.0%)	68.6% (60.8% - 76.1%)	66 (53,79)	60 (48,70)
Namibia	Upper middle	Full sharing	54.3%	70.9% (61.1% - 79.2%)	52.8% (36.5% - 69.9%)	53 (43,61)	41 (33,52)
Nepal	Lower middle	Default	15.1%	80.3% (75.2% - 84.4%)	80.3% (75.2% - 84.4%)	178 (127,236)	178 (127,236)
Nepal	Lower middle	2 dose threshold	27.2%	80.3% (75.2% - 84.4%)	80.3% (75.2% - 84.4%)	178 (127,235)	178 (127,235)
Nepal	Lower middle	40+ threshold	44.4%	80.1% (75.0% - 84.3%)	80.1% (74.9% - 84.3%)	177 (126,234)	177 (126,234)
Nepal	Lower middle	65+ threshold	53.5%	80.0% (74.8% - 84.3%)	78.3% (73.8% - 83.0%)	184 (131,246)	170 (122,221)
Nepal	Lower middle	Full sharing	54.0%	78.2% (73.0% - 83.0%)	62.6% (54.7% - 69.0%)	180 (129,236)	148 (92,195)
Netherlands	High	Default	89.4%	39.1% (33.8% - 44.2%)	39.1% (33.8% - 44.2%)	307 (260,347)	307 (260,347)
Netherlands	High	2 dose threshold	91.2%	39.0% (33.8% - 44.1%)	39.0% (33.8% - 44.1%)	307 (260,347)	307 (260,347)
Netherlands	High	40+ threshold	70.9%	38.9% (33.7% - 44.0%)	38.5% (33.2% - 43.7%)	306 (259,346)	306 (260,346)
Netherlands	High	65+ threshold	58.2%	45.5% (40.7% - 49.1%)	38.0% (32.4% - 47.7%)	311 (261,353)	303 (259,344)
Netherlands	High	Full sharing	52.9%	47.8% (41.9% - 52.9%)	22.6% (17.1% - 36.4%)	326 (275,367)	221 (188,280)
New Zealand	High	Default	23.3%	0.1% (0.1% - 0.1%)	0.1% (0.1% - 0.1%)	1 (1,1)	1 (1,1)
New Zealand	High	2 dose threshold	34.4%	0.1% (0.1% - 0.1%)	0.1% (0.1% - 0.1%)	1 (1,1)	1 (1,1)
New Zealand	High	40+ threshold	51.4%	0.1% (0.1% - 0.1%)	0.1% (0.1% - 0.1%)	1 (1,1)	1 (1,1)
New Zealand	High	65+ threshold	53.1%	0.1% (0.1% - 0.1%)	0.1% (0.1% - 0.1%)	1 (1,1)	1 (1,1)
New Zealand	High	Full sharing	53.2%	0.1% (0.1% - 0.1%)	0.1% (0.1% - 0.1%)	1 (1,1)	1 (1,1)
Nicaragua	Lower middle	Default	6.0%	13.3% (9.5% - 17.2%)	13.3% (9.5% - 17.2%)	12 (10,14)	12 (10,14)
Nicaragua	Lower middle	2 dose threshold	19.3%	13.3% (9.5% - 17.2%)	13.3% (9.5% - 17.2%)	12 (10,14)	12 (10,14)
Nicaragua	Lower middle	40+ threshold	38.5%	12.5% (8.7% - 16.2%)	12.5% (8.6% - 16.1%)	11 (9,12)	11 (9,12)
Nicaragua	Lower middle	65+ threshold	51.3%	9.1% (6.2% - 11.5%)	7.9% (5.6% - 10.1%)	8 (7,9)	8 (7,9)

Country	Income bracket	Strategy	Proportion vaccinated	Proportion infected		Mortalities per 100,000	
				unchanged	adapted	unchanged	adapted
Nicaragua	Lower middle	Full sharing	53.9%	7.5% (5.4% - 9.5%)	5.8% (4.0% - 7.4%)	7 (6,8)	6 (5,7)
Niger	Low	Default	2.4%	47.3% (37.9% - 55.4%)	47.3% (37.9% - 55.4%)	39 (31,52)	39 (31,52)
Niger	Low	2 dose threshold	16.5%	45.9% (36.9% - 53.7%)	45.9% (36.7% - 53.6%)	38 (30,51)	38 (30,51)
Niger	Low	40+ threshold	36.4%	39.1% (31.9% - 46.3%)	38.4% (31.2% - 45.8%)	34 (28,44)	34 (27,44)
Niger	Low	65+ threshold	52.3%	34.7% (26.2% - 42.1%)	34.6% (26.0% - 42.0%)	32 (25,41)	32 (25,41)
Niger	Low	Full sharing	55.1%	34.0% (25.4% - 41.3%)	27.7% (18.1% - 42.8%)	29 (22,36)	23 (19,29)
Nigeria	Lower middle	Default	2.4%	53.4% (46.9% - 59.8%)	53.4% (46.9% - 59.8%)	51 (40,62)	51 (40,62)
Nigeria	Lower middle	2 dose threshold	16.4%	52.4% (45.6% - 58.9%)	52.3% (45.7% - 58.9%)	51 (39,61)	51 (39,61)
Nigeria	Lower middle	40+ threshold	36.4%	47.6% (40.8% - 54.6%)	47.1% (40.3% - 53.8%)	46 (36,57)	46 (36,57)
Nigeria	Lower middle	65+ threshold	52.1%	43.3% (38.0% - 48.9%)	43.1% (38.0% - 48.6%)	44 (33,55)	43 (33,55)
Nigeria	Lower middle	Full sharing	54.7%	42.1% (37.2% - 47.3%)	32.4% (27.3% - 38.6%)	38 (29,48)	29 (23,38)
Norway	High	Default	76.5%	52.7% (37.2% - 65.0%)	52.7% (37.2% - 65.0%)	20 (18,22)	20 (18,22)
Norway	High	2 dose threshold	81.5%	50.7% (35.3% - 63.6%)	50.5% (35.2% - 63.6%)	20 (18,22)	20 (18,22)
Norway	High	40+ threshold	66.1%	54.8% (38.1% - 67.8%)	28.8% (16.1% - 40.8%)	19 (18,21)	19 (17,20)
Norway	High	65+ threshold	57.7%	72.0% (64.2% - 77.8%)	35.1% (21.3% - 47.4%)	22 (19,26)	19 (17,22)
Norway	High	Full sharing	53.1%	76.7% (68.8% - 81.0%)	12.1% (5.9% - 19.6%)	25 (22,29)	12 (11,13)
Oman	High	Default	32.2%	58.6% (52.5% - 64.8%)	58.6% (52.5% - 64.8%)	189 (155,229)	189 (155,229)
Oman	High	2 dose threshold	42.8%	58.5% (52.4% - 64.7%)	58.5% (52.4% - 64.8%)	188 (154,228)	188 (154,228)
Oman	High	40+ threshold	41.3%	57.9% (51.9% - 64.1%)	57.9% (52.0% - 64.3%)	183 (150,221)	183 (150,222)
Oman	High	65+ threshold	51.5%	53.3% (47.2% - 59.6%)	47.8% (40.4% - 55.3%)	159 (131,191)	144 (121,172)
Oman	High	Full sharing	53.5%	42.8% (36.7% - 49.5%)	33.6% (24.8% - 46.9%)	126 (104,150)	104 (84,141)
Pakistan	Lower middle	Default	11.2%	78.1% (75.1% - 80.9%)	78.1% (75.1% - 80.9%)	157 (122,200)	157 (122,200)
Pakistan	Lower middle	2 dose threshold	24.0%	78.0% (75.0% - 80.8%)	78.0% (75.0% - 80.8%)	156 (122,199)	156 (122,199)
Pakistan	Lower middle	40+ threshold	40.5%	77.0% (74.0% - 79.9%)	76.9% (73.8% - 79.7%)	152 (119,192)	152 (119,192)
Pakistan	Lower middle	65+ threshold	51.8%	73.3% (69.4% - 77.0%)	72.3% (67.8% - 76.3%)	133 (105,168)	133 (105,167)
Pakistan	Lower middle	Full sharing	54.3%	70.0% (65.4% - 74.6%)	57.7% (52.2% - 62.1%)	113 (89,141)	98 (78,122)
Palestine	Lower middle	Default	19.5%	71.4% (66.1% - 76.2%)	71.4% (66.1% - 76.2%)	170 (128,215)	170 (128,215)
Palestine	Lower middle	2 dose threshold	31.3%	71.1% (65.9% - 76.1%)	71.1% (65.9% - 76.1%)	169 (127,214)	169 (127,214)
Palestine	Lower middle	40+ threshold	44.5%	69.7% (64.0% - 76.0%)	69.4% (63.6% - 76.0%)	166 (125,208)	166 (125,208)
Palestine	Lower middle	65+ threshold	51.8%	68.1% (61.8% - 75.4%)	67.7% (60.5% - 75.1%)	150 (111,190)	150 (111,189)
Palestine	Lower middle	Full sharing	54.5%	65.9% (59.1% - 73.0%)	48.9% (38.8% - 56.9%)	118 (86,150)	85 (64,107)
Panama	High	Default	100.0%	53.2% (48.5% - 58.3%)	53.2% (48.5% - 58.3%)	186 (157,223)	186 (157,223)
Panama	High	2 dose threshold	96.2%	53.1% (48.5% - 58.3%)	53.1% (48.5% - 58.3%)	186 (157,222)	186 (157,222)
Panama	High	40+ threshold	59.4%	53.1% (48.7% - 57.9%)	53.1% (48.6% - 57.8%)	185 (156,222)	185 (156,222)
Panama	High	65+ threshold	54.9%	53.1% (49.2% - 56.9%)	50.1% (45.5% - 56.0%)	182 (153,219)	180 (151,215)

Country	Income bracket	Strategy	Proportion vaccinated	Proportion infected		Mortalities per 100,000	
				unchanged	adapted	unchanged	adapted
Panama	High	Full sharing	53.6%	50.1% (46.1% - 55.5%)	49.9% (38.6% - 54.0%)	172 (145,205)	171 (146,204)
Paraguay	Upper middle	Default	18.7%	74.2% (69.4% - 77.8%)	74.2% (69.4% - 77.8%)	207 (171,256)	207 (171,256)
Paraguay	Upper middle	2 dose threshold	30.6%	74.1% (69.4% - 77.7%)	74.1% (69.4% - 77.7%)	206 (170,255)	206 (170,255)
Paraguay	Upper middle	40+ threshold	44.4%	73.8% (69.1% - 77.4%)	73.8% (69.0% - 77.4%)	201 (166,249)	201 (166,249)
Paraguay	Upper middle	65+ threshold	51.8%	70.3% (65.2% - 74.2%)	68.5% (62.8% - 73.5%)	159 (131,198)	156 (129,194)
Paraguay	Upper middle	Full sharing	53.8%	62.8% (57.7% - 68.1%)	40.4% (29.9% - 55.5%)	120 (101,146)	76 (62,100)
Peru	Upper middle	Default	23.5%	71.6% (66.3% - 76.5%)	71.6% (66.3% - 76.5%)	667 (534,834)	667 (534,834)
Peru	Upper middle	2 dose threshold	34.7%	71.6% (66.2% - 76.5%)	71.6% (66.2% - 76.5%)	666 (533,833)	666 (533,833)
Peru	Upper middle	40+ threshold	48.1%	71.2% (65.5% - 76.3%)	71.1% (65.3% - 76.3%)	659 (529,822)	659 (528,822)
Peru	Upper middle	65+ threshold	52.7%	69.1% (63.0% - 74.7%)	68.6% (61.9% - 74.3%)	618 (496,772)	616 (495,771)
Peru	Upper middle	Full sharing	53.5%	67.1% (60.4% - 72.9%)	60.5% (55.5% - 64.4%)	560 (457,691)	478 (378,577)
Philippines	Lower middle	Default	13.3%	56.8% (48.4% - 65.3%)	56.8% (48.4% - 65.3%)	125 (95,154)	125 (95,154)
Philippines	Lower middle	2 dose threshold	25.8%	56.7% (48.3% - 65.3%)	56.7% (48.3% - 65.3%)	124 (94,153)	124 (94,153)
Philippines	Lower middle	40+ threshold	42.1%	55.3% (46.8% - 63.9%)	55.0% (46.6% - 63.3%)	115 (88,140)	115 (88,140)
Philippines	Lower middle	65+ threshold	51.8%	47.5% (37.7% - 57.3%)	50.6% (43.1% - 58.8%)	81 (63,95)	82 (63,102)
Philippines	Lower middle	Full sharing	54.0%	39.8% (27.6% - 53.4%)	24.9% (13.4% - 41.5%)	60 (48,70)	43 (31,62)
Poland	High	Default	64.7%	63.1% (54.3% - 72.1%)	63.1% (54.3% - 72.1%)	258 (196,331)	258 (196,331)
Poland	High	2 dose threshold	70.1%	63.0% (54.1% - 72.0%)	63.0% (54.2% - 72.0%)	258 (196,331)	258 (196,331)
Poland	High	40+ threshold	68.1%	62.9% (53.9% - 72.1%)	62.7% (53.8% - 70.7%)	257 (196,331)	257 (196,331)
Poland	High	65+ threshold	57.2%	62.8% (52.8% - 72.0%)	64.2% (54.2% - 71.8%)	256 (195,327)	257 (196,330)
Poland	High	Full sharing	53.0%	60.8% (49.8% - 71.0%)	48.6% (36.9% - 60.7%)	276 (208,359)	230 (181,274)
Portugal	High	Default	77.8%	34.0% (29.9% - 37.9%)	34.0% (29.9% - 37.9%)	327 (284,384)	327 (284,384)
Portugal	High	2 dose threshold	81.9%	34.0% (29.9% - 37.9%)	34.0% (29.9% - 37.9%)	327 (284,384)	327 (284,384)
Portugal	High	40+ threshold	71.7%	34.0% (29.9% - 37.8%)	34.0% (29.9% - 37.8%)	327 (284,384)	327 (284,384)
Portugal	High	65+ threshold	59.0%	34.1% (30.0% - 37.8%)	34.0% (29.9% - 37.8%)	326 (283,383)	326 (282,382)
Portugal	High	Full sharing	53.1%	34.5% (30.5% - 38.3%)	20.1% (14.3% - 38.2%)	346 (300,405)	207 (165,325)
Puerto Rico	High	Default	0.0%	31.9% (28.0% - 36.1%)	31.9% (28.0% - 36.1%)	243 (205,282)	243 (205,282)
Puerto Rico	High	2 dose threshold	13.5%	31.8% (27.9% - 35.9%)	31.8% (27.8% - 35.9%)	242 (204,281)	242 (204,281)
Puerto Rico	High	40+ threshold	33.7%	30.8% (27.0% - 34.8%)	30.7% (26.8% - 34.8%)	224 (188,258)	224 (189,259)
Puerto Rico	High	65+ threshold	50.5%	24.5% (21.0% - 28.3%)	22.8% (19.1% - 26.9%)	169 (144,192)	165 (140,187)
Puerto Rico	High	Full sharing	53.1%	19.8% (16.9% - 23.0%)	13.8% (10.4% - 29.3%)	144 (123,163)	105 (85,169)
Qatar	High	Default	88.5%	66.7% (59.6% - 73.3%)	66.7% (59.6% - 73.3%)	28 (21,35)	28 (21,35)
Qatar	High	2 dose threshold	90.7%	66.7% (59.6% - 73.3%)	66.7% (59.6% - 73.3%)	28 (21,35)	28 (21,35)
Qatar	High	40+ threshold	49.2%	68.5% (62.0% - 74.9%)	68.0% (60.8% - 74.4%)	28 (21,35)	28 (21,35)
Qatar	High	65+ threshold	51.8%	71.4% (64.9% - 77.8%)	70.5% (64.0% - 77.1%)	30 (23,37)	30 (22,37)

Country	Income bracket	Strategy	Proportion vaccinated	Proportion infected		Mortalities per 100,000	
				unchanged	adapted	unchanged	adapted
Qatar	High	Full sharing	53.1%	68.9% (62.0% - 76.2%)	61.1% (57.8% - 64.6%)	28 (21,34)	24 (18,32)
Republic of Macedonia	Upper middle	Default	52.1%	85.7% (80.4% - 90.1%)	85.7% (80.4% - 90.1%)	417 (322,505)	417 (322,505)
Republic of Macedonia	Upper middle	2 dose threshold	59.4%	85.5% (80.3% - 89.9%)	85.5% (80.3% - 89.9%)	416 (321,505)	416 (321,504)
Republic of Macedonia	Upper middle	40+ threshold	64.1%	84.7% (78.8% - 89.4%)	82.4% (77.5% - 86.7%)	413 (319,501)	413 (319,501)
Republic of Macedonia	Upper middle	65+ threshold	51.7%	86.4% (80.6% - 91.0%)	76.0% (68.8% - 83.9%)	395 (298,482)	390 (297,473)
Republic of Macedonia	Upper middle	Full sharing	53.0%	84.5% (75.8% - 91.5%)	66.7% (58.7% - 73.6%)	356 (270,432)	298 (223,367)
Romania	High	Default	35.5%	73.4% (67.0% - 78.5%)	73.4% (67.0% - 78.5%)	313 (252,376)	313 (252,376)
Romania	High	2 dose threshold	44.7%	73.3% (66.9% - 78.4%)	73.3% (66.8% - 78.4%)	313 (252,375)	313 (252,375)
Romania	High	40+ threshold	58.3%	71.3% (64.7% - 76.9%)	70.3% (63.5% - 75.6%)	309 (249,370)	308 (248,369)
Romania	High	65+ threshold	57.4%	69.6% (61.7% - 74.7%)	51.7% (43.7% - 58.8%)	304 (245,366)	290 (235,348)
Romania	High	Full sharing	53.0%	64.4% (52.1% - 72.6%)	43.8% (30.8% - 55.3%)	328 (262,398)	278 (230,340)
Russian Federation	Upper middle	Default	26.2%	77.4% (68.0% - 85.1%)	77.4% (68.0% - 85.1%)	168 (138,198)	168 (138,198)
Russian Federation	Upper middle	2 dose threshold	37.0%	77.4% (67.9% - 85.0%)	77.4% (67.9% - 85.0%)	168 (137,198)	168 (137,198)
Russian Federation	Upper middle	40+ threshold	52.7%	77.2% (67.5% - 85.1%)	76.8% (67.4% - 84.5%)	166 (137,196)	166 (137,196)
Russian Federation	Upper middle	65+ threshold	54.9%	75.8% (64.3% - 84.9%)	72.3% (56.9% - 84.4%)	159 (133,185)	155 (133,175)
Russian Federation	Upper middle	Full sharing	54.1%	77.0% (62.2% - 86.7%)	58.3% (39.7% - 69.9%)	158 (134,182)	144 (122,165)
Rwanda	Low	Default	5.8%	61.1% (50.8% - 69.9%)	61.1% (50.8% - 69.9%)	47 (35,63)	47 (35,63)
Rwanda	Low	2 dose threshold	19.3%	61.0% (50.5% - 69.8%)	61.0% (50.5% - 69.8%)	47 (35,63)	47 (35,63)
Rwanda	Low	40+ threshold	38.6%	59.8% (48.8% - 69.1%)	59.9% (49.1% - 69.2%)	45 (34,60)	45 (34,60)
Rwanda	Low	65+ threshold	52.9%	52.3% (40.8% - 63.1%)	23.8% (17.4% - 30.4%)	35 (27,44)	25 (20,32)
Rwanda	Low	Full sharing	54.5%	33.8% (23.9% - 47.3%)	10.3% (6.8% - 14.0%)	26 (21,32)	10 (8,12)
Sao Tome and Principe	Lower middle	Default	20.1%	69.4% (61.4% - 79.6%)	69.4% (61.4% - 79.6%)	76 (54,102)	76 (54,102)
Sao Tome and Principe	Lower middle	2 dose threshold	32.2%	67.7% (59.6% - 78.7%)	67.6% (59.4% - 78.6%)	76 (54,102)	76 (54,102)
Sao Tome and Principe	Lower middle	40+ threshold	42.9%	59.0% (49.2% - 71.9%)	55.7% (45.1% - 70.2%)	73 (52,96)	72 (51,96)
Sao Tome and Principe	Lower middle	65+ threshold	53.0%	52.0% (42.0% - 63.8%)	51.0% (41.1% - 61.5%)	70 (49,93)	70 (49,93)
Sao Tome and Principe	Lower middle	Full sharing	54.8%	50.7% (40.8% - 61.1%)	33.8% (19.5% - 48.3%)	61 (43,81)	37 (29,58)
Saudi Arabia	High	Default	76.1%	38.4% (31.9% - 44.1%)	38.4% (31.9% - 44.1%)	137 (114,165)	137 (114,165)
Saudi Arabia	High	2 dose threshold	86.4%	38.3% (31.8% - 43.9%)	38.4% (31.9% - 44.0%)	136 (114,164)	136 (114,164)
Saudi Arabia	High	40+ threshold	68.0%	37.9% (31.5% - 43.4%)	37.9% (31.6% - 43.4%)	128 (106,155)	128 (106,155)
Saudi Arabia	High	65+ threshold	51.0%	33.4% (27.6% - 39.0%)	36.6% (30.1% - 43.5%)	101 (84,120)	103 (87,125)
Saudi Arabia	High	Full sharing	53.7%	29.1% (23.7% - 33.9%)	31.9% (20.3% - 43.0%)	86 (72,104)	93 (70,130)
Senegal	Lower middle	Default	6.6%	75.3% (68.1% - 80.9%)	75.3% (68.1% - 80.9%)	84 (65,105)	84 (65,105)
Senegal	Lower middle	2 dose threshold	20.0%	75.2% (68.0% - 80.9%)	75.2% (67.9% - 80.9%)	84 (65,105)	84 (65,105)
Senegal	Lower middle	40+ threshold	39.3%	73.4% (65.9% - 79.1%)	73.3% (65.8% - 79.1%)	81 (63,100)	81 (63,100)
Senegal	Lower middle	65+ threshold	52.8%	59.7% (51.8% - 67.6%)	51.1% (44.1% - 57.3%)	73 (56,90)	71 (55,88)

Country	Income bracket	Strategy	Proportion vaccinated	Proportion infected		Mortalities per 100,000	
				unchanged	adapted	unchanged	adapted
Senegal	Lower middle	Full sharing	54.7%	52.2% (44.1% - 60.5%)	35.6% (24.0% - 51.3%)	61 (48,75)	43 (33,57)
Serbia	Upper middle	Default	44.8%	72.9% (64.6% - 79.9%)	72.9% (64.6% - 79.9%)	175 (141,219)	175 (141,219)
Serbia	Upper middle	2 dose threshold	52.8%	72.8% (64.4% - 79.8%)	72.8% (64.4% - 79.8%)	175 (141,219)	175 (141,219)
Serbia	Upper middle	40+ threshold	64.0%	71.1% (62.2% - 78.5%)	70.4% (61.6% - 77.9%)	174 (140,217)	173 (140,217)
Serbia	Upper middle	65+ threshold	57.8%	74.5% (66.8% - 80.9%)	55.8% (45.3% - 65.0%)	177 (142,222)	166 (133,213)
Serbia	Upper middle	Full sharing	53.0%	72.7% (62.3% - 80.6%)	43.8% (31.0% - 55.8%)	205 (163,262)	163 (138,202)
Sierra Leone	Low	Default	2.4%	40.7% (34.4% - 45.7%)	40.7% (34.4% - 45.7%)	58 (49,70)	58 (49,70)
Sierra Leone	Low	2 dose threshold	16.4%	40.6% (34.3% - 45.7%)	40.6% (34.3% - 45.7%)	58 (48,69)	58 (48,69)
Sierra Leone	Low	40+ threshold	36.6%	39.5% (33.4% - 44.4%)	39.5% (33.2% - 44.6%)	53 (44,63)	53 (44,63)
Sierra Leone	Low	65+ threshold	52.1%	31.1% (25.6% - 35.6%)	28.5% (23.0% - 33.7%)	41 (35,49)	41 (34,48)
Sierra Leone	Low	Full sharing	54.6%	27.3% (22.3% - 31.2%)	22.3% (16.7% - 31.8%)	36 (30,43)	30 (25,36)
Singapore	High	Default	83.8%	14.7% (12.0% - 17.6%)	14.7% (12.0% - 17.6%)	2 (2,2)	2 (2,2)
Singapore	High	2 dose threshold	87.5%	14.6% (12.0% - 17.3%)	14.6% (12.0% - 17.4%)	2 (2,2)	2 (2,2)
Singapore	High	40+ threshold	67.9%	14.4% (10.6% - 18.6%)	5.3% (3.5% - 8.6%)	2 (2,2)	1 (1,2)
Singapore	High	65+ threshold	54.6%	78.9% (71.3% - 84.3%)	4.2% (2.7% - 6.0%)	6 (5,9)	1 (1,1)
Singapore	High	Full sharing	53.0%	67.6% (40.7% - 80.6%)	2.9% (1.7% - 4.3%)	5 (3,8)	1 (1,1)
Slovakia	High	Default	54.6%	52.9% (47.0% - 59.3%)	52.9% (47.0% - 59.3%)	112 (95,135)	112 (95,135)
Slovakia	High	2 dose threshold	61.4%	51.7% (45.8% - 58.3%)	51.7% (45.8% - 58.2%)	112 (95,135)	112 (95,135)
Slovakia	High	40+ threshold	66.2%	49.0% (43.1% - 55.0%)	43.9% (39.1% - 49.1%)	112 (95,134)	111 (94,134)
Slovakia	High	65+ threshold	56.5%	58.9% (54.4% - 64.9%)	43.2% (38.1% - 49.2%)	113 (95,136)	111 (94,133)
Slovakia	High	Full sharing	53.0%	52.5% (45.5% - 60.2%)	25.7% (17.9% - 35.1%)	125 (105,150)	70 (62,83)
Slovenia	High	Default	58.9%	64.9% (54.5% - 73.9%)	64.9% (54.5% - 73.9%)	337 (287,401)	337 (287,401)
Slovenia	High	2 dose threshold	64.9%	64.5% (53.9% - 73.6%)	64.4% (53.8% - 73.5%)	337 (287,401)	337 (287,401)
Slovenia	High	40+ threshold	69.1%	62.8% (51.3% - 72.7%)	60.8% (49.0% - 70.6%)	335 (286,398)	334 (284,396)
Slovenia	High	65+ threshold	58.1%	68.8% (59.7% - 76.9%)	51.2% (40.8% - 61.7%)	341 (289,407)	326 (277,387)
Slovenia	High	Full sharing	53.5%	66.6% (55.0% - 76.4%)	38.6% (24.1% - 56.6%)	355 (299,425)	278 (234,346)
South Africa	Upper middle	Default	16.4%	84.2% (75.7% - 95.7%)	84.2% (75.7% - 95.7%)	165 (130,243)	165 (130,243)
South Africa	Upper middle	2 dose threshold	28.6%	83.9% (75.7% - 94.9%)	83.9% (75.7% - 94.9%)	165 (130,242)	165 (130,242)
South Africa	Upper middle	40+ threshold	43.9%	82.7% (74.7% - 94.3%)	81.9% (73.4% - 94.3%)	163 (129,231)	163 (129,232)
South Africa	Upper middle	65+ threshold	52.2%	79.5% (72.5% - 92.7%)	74.8% (60.5% - 81.2%)	156 (128,198)	153 (127,181)
South Africa	Upper middle	Full sharing	54.0%	76.7% (64.5% - 90.1%)	73.6% (54.6% - 80.5%)	153 (127,188)	151 (127,178)
South Korea	High	Default	41.9%	8.0% (6.8% - 9.6%)	8.0% (6.8% - 9.6%)	12 (11,13)	12 (11,13)
South Korea	High	2 dose threshold	50.2%	7.4% (6.1% - 9.0%)	7.4% (6.1% - 9.0%)	12 (11,13)	12 (11,13)
South Korea	High	40+ threshold	64.0%	5.5% (4.2% - 7.0%)	5.0% (3.5% - 6.5%)	11 (10,12)	11 (10,12)
South Korea	High	65+ threshold	52.4%	3.7% (2.7% - 4.8%)	5.5% (2.5% - 10.1%)	7 (7,8)	8 (6,12)

Country	Income bracket	Strategy	Proportion vaccinated	Proportion infected		Mortalities per 100,000	
				unchanged	adapted	unchanged	adapted
South Korea	High	Full sharing	52.9%	2.2% (1.7% - 3.0%)	1.0% (0.7% - 1.2%)	6 (5,6)	3 (3,3)
South Sudan	Low	Default	0.8%	54.7% (41.0% - 65.6%)	54.7% (41.0% - 65.6%)	54 (41,70)	54 (41,70)
South Sudan	Low	2 dose threshold	15.0%	54.7% (41.0% - 65.6%)	54.7% (41.0% - 65.6%)	54 (41,70)	54 (41,70)
South Sudan	Low	40+ threshold	35.6%	54.7% (41.0% - 65.6%)	54.7% (41.0% - 65.6%)	54 (40,69)	54 (40,69)
South Sudan	Low	65+ threshold	52.0%	54.7% (41.0% - 65.5%)	54.6% (40.9% - 65.5%)	53 (39,68)	53 (39,68)
South Sudan	Low	Full sharing	54.6%	54.1% (40.6% - 64.9%)	35.8% (22.3% - 50.9%)	46 (34,59)	28 (22,36)
Spain	High	Default	79.7%	45.3% (40.1% - 50.4%)	45.3% (40.1% - 50.4%)	435 (398,476)	435 (398,476)
Spain	High	2 dose threshold	83.4%	45.3% (40.1% - 50.4%)	45.3% (40.1% - 50.3%)	435 (397,476)	435 (397,476)
Spain	High	40+ threshold	71.0%	44.9% (39.8% - 49.6%)	44.1% (38.4% - 48.8%)	433 (395,474)	433 (395,474)
Spain	High	65+ threshold	58.0%	51.5% (47.2% - 55.8%)	36.9% (33.0% - 40.6%)	443 (405,488)	421 (383,462)
Spain	High	Full sharing	52.8%	49.9% (41.6% - 55.5%)	22.8% (19.9% - 26.3%)	496 (453,547)	297 (263,339)
Sri Lanka	Lower middle	Default	21.4%	32.0% (29.3% - 34.4%)	32.0% (29.3% - 34.4%)	12 (8,17)	12 (8,17)
Sri Lanka	Lower middle	2 dose threshold	32.9%	31.9% (29.2% - 34.1%)	31.9% (29.2% - 34.1%)	12 (8,17)	12 (8,17)
Sri Lanka	Lower middle	40+ threshold	48.1%	30.4% (28.1% - 32.3%)	30.1% (28.1% - 31.9%)	11 (8,16)	11 (8,15)
Sri Lanka	Lower middle	65+ threshold	54.3%	22.0% (18.9% - 24.9%)	28.3% (14.5% - 39.4%)	7 (5,9)	8 (5,12)
Sri Lanka	Lower middle	Full sharing	53.5%	17.1% (12.8% - 21.6%)	3.3% (2.0% - 5.8%)	6 (5,8)	2 (1,2)
Sudan	Low	Default	2.5%	49.2% (40.0% - 59.3%)	49.2% (40.0% - 59.3%)	83 (67,104)	83 (67,104)
Sudan	Low	2 dose threshold	16.4%	49.2% (40.0% - 59.2%)	49.2% (40.0% - 59.3%)	83 (66,104)	83 (66,104)
Sudan	Low	40+ threshold	36.8%	48.8% (39.5% - 58.8%)	48.8% (39.4% - 58.9%)	80 (64,100)	80 (65,100)
Sudan	Low	65+ threshold	51.9%	45.1% (36.2% - 54.6%)	45.2% (35.7% - 58.7%)	66 (51,84)	66 (52,85)
Sudan	Low	Full sharing	54.5%	40.0% (32.7% - 47.0%)	38.2% (28.5% - 42.4%)	56 (45,71)	54 (42,69)
Suriname	Upper middle	Default	45.8%	54.4% (46.3% - 60.9%)	54.4% (46.3% - 60.9%)	162 (129,206)	162 (129,206)
Suriname	Upper middle	2 dose threshold	54.2%	54.1% (46.1% - 60.5%)	54.1% (46.0% - 60.4%)	161 (129,205)	161 (129,205)
Suriname	Upper middle	40+ threshold	53.4%	53.5% (45.4% - 60.2%)	52.3% (44.6% - 58.3%)	157 (125,199)	157 (126,199)
Suriname	Upper middle	65+ threshold	53.2%	54.4% (43.6% - 64.0%)	46.1% (38.3% - 51.6%)	132 (107,165)	130 (103,164)
Suriname	Upper middle	Full sharing	53.8%	66.2% (52.0% - 75.6%)	17.8% (11.2% - 32.3%)	115 (96,135)	55 (42,75)
Sweden	High	Default	76.5%	34.3% (29.6% - 39.4%)	34.3% (29.6% - 39.4%)	249 (213,288)	249 (213,288)
Sweden	High	2 dose threshold	80.8%	34.3% (29.6% - 39.4%)	34.3% (29.6% - 39.4%)	249 (213,288)	249 (213,288)
Sweden	High	40+ threshold	70.1%	33.9% (29.3% - 39.0%)	33.9% (29.3% - 39.0%)	248 (212,287)	248 (212,287)
Sweden	High	65+ threshold	59.0%	35.6% (31.2% - 40.5%)	35.0% (31.0% - 40.3%)	249 (213,289)	247 (211,288)
Sweden	High	Full sharing	53.1%	34.3% (29.3% - 39.2%)	21.6% (16.6% - 38.4%)	275 (233,321)	193 (164,257)
Switzerland	High	Default	73.2%	32.2% (28.8% - 36.0%)	32.2% (28.8% - 36.0%)	246 (216,277)	246 (216,277)
Switzerland	High	2 dose threshold	77.3%	31.9% (28.4% - 35.6%)	31.9% (28.4% - 35.6%)	246 (216,277)	246 (216,277)
Switzerland	High	40+ threshold	68.8%	32.5% (29.1% - 36.4%)	30.4% (26.4% - 34.3%)	245 (215,276)	245 (215,275)
Switzerland	High	65+ threshold	57.8%	42.9% (36.8% - 49.5%)	28.6% (24.4% - 34.0%)	250 (220,281)	242 (213,270)

Country	Income bracket	Strategy	Proportion vaccinated	Proportion infected		Mortalities per 100,000	
				unchanged	adapted	unchanged	adapted
Switzerland	High	Full sharing	52.8%	42.1% (32.6% - 49.6%)	19.0% (16.3% - 23.7%)	277 (243,307)	197 (164,229)
Syria	Low	Default	1.1%	23.5% (19.4% - 28.2%)	23.5% (19.4% - 28.2%)	113 (94,139)	113 (94,139)
Syria	Low	2 dose threshold	15.0%	23.3% (19.2% - 28.0%)	23.3% (19.2% - 28.0%)	111 (91,136)	111 (91,136)
Syria	Low	40+ threshold	35.6%	21.3% (17.2% - 25.9%)	20.9% (16.7% - 25.5%)	87 (73,103)	86 (72,102)
Syria	Low	65+ threshold	51.4%	14.6% (11.6% - 18.4%)	17.6% (12.9% - 24.3%)	59 (51,70)	62 (52,73)
Syria	Low	Full sharing	54.0%	12.3% (9.7% - 15.4%)	9.3% (5.5% - 19.9%)	48 (41,56)	34 (26,55)
Tajikistan	Low	Default	5.7%	81.8% (79.3% - 83.5%)	81.8% (79.3% - 83.5%)	20 (11,32)	20 (11,32)
Tajikistan	Low	2 dose threshold	19.3%	81.8% (79.2% - 83.4%)	81.8% (79.2% - 83.4%)	20 (10,31)	20 (10,31)
Tajikistan	Low	40+ threshold	37.2%	81.4% (78.9% - 83.1%)	81.4% (78.7% - 83.1%)	19 (10,30)	19 (10,30)
Tajikistan	Low	65+ threshold	51.5%	77.8% (74.2% - 80.9%)	76.4% (72.4% - 80.5%)	14 (9,23)	14 (9,23)
Tajikistan	Low	Full sharing	54.2%	72.4% (65.7% - 78.3%)	50.9% (33.6% - 61.9%)	11 (7,16)	7 (5,10)
Thailand	Upper middle	Default	16.1%	29.0% (25.6% - 33.5%)	29.0% (25.6% - 33.5%)	25 (18,34)	25 (18,34)
Thailand	Upper middle	2 dose threshold	28.2%	28.9% (25.4% - 33.3%)	28.9% (25.5% - 33.3%)	25 (18,33)	25 (18,33)
Thailand	Upper middle	40+ threshold	46.9%	27.6% (24.0% - 32.0%)	27.3% (23.8% - 31.7%)	22 (16,29)	22 (16,29)
Thailand	Upper middle	65+ threshold	51.3%	19.0% (15.3% - 23.0%)	15.1% (9.7% - 22.5%)	9 (7,12)	8 (5,10)
Thailand	Upper middle	Full sharing	53.2%	8.9% (5.6% - 12.3%)	0.4% (0.3% - 0.7%)	4 (3,6)	0 (0,1)
Togo	Low	Default	7.0%	48.9% (40.8% - 55.3%)	48.9% (40.8% - 55.3%)	68 (56,84)	68 (56,84)
Togo	Low	2 dose threshold	20.4%	48.8% (40.7% - 55.2%)	48.8% (40.7% - 55.2%)	68 (55,84)	68 (55,84)
Togo	Low	40+ threshold	39.8%	47.0% (38.8% - 53.2%)	47.0% (38.9% - 53.5%)	63 (52,77)	63 (52,77)
Togo	Low	65+ threshold	52.0%	34.5% (28.4% - 41.0%)	27.8% (22.8% - 31.8%)	49 (42,60)	47 (39,57)
Togo	Low	Full sharing	54.6%	27.2% (21.8% - 31.8%)	14.3% (9.6% - 29.5%)	40 (34,49)	21 (17,30)
Trinidad and Tobago	High	Default	23.7%	57.1% (45.3% - 66.5%)	57.1% (45.3% - 66.5%)	169 (138,214)	169 (138,214)
Trinidad and Tobago	High	2 dose threshold	34.8%	56.3% (44.6% - 66.1%)	56.2% (44.6% - 65.9%)	166 (137,212)	166 (137,212)
Trinidad and Tobago	High	40+ threshold	50.6%	50.7% (38.9% - 60.3%)	47.7% (36.5% - 57.3%)	152 (125,190)	150 (125,190)
Trinidad and Tobago	High	65+ threshold	51.3%	42.1% (28.7% - 55.4%)	28.4% (21.8% - 33.9%)	114 (95,141)	105 (86,135)
Trinidad and Tobago	High	Full sharing	53.4%	36.9% (19.5% - 56.6%)	8.7% (4.5% - 15.3%)	79 (67,95)	27 (19,43)
Tunisia	Lower middle	Default	18.4%	79.1% (72.7% - 83.4%)	79.1% (72.7% - 83.4%)	253 (196,340)	253 (196,340)
Tunisia	Lower middle	2 dose threshold	30.3%	79.1% (72.7% - 83.4%)	79.1% (72.7% - 83.4%)	252 (195,338)	252 (195,339)
Tunisia	Lower middle	40+ threshold	46.6%	78.7% (72.4% - 83.1%)	78.7% (72.5% - 83.2%)	245 (191,329)	245 (191,329)
Tunisia	Lower middle	65+ threshold	51.7%	74.7% (67.5% - 79.9%)	73.0% (64.2% - 78.7%)	200 (160,263)	197 (158,259)
Tunisia	Lower middle	Full sharing	53.4%	68.0% (56.7% - 76.5%)	50.7% (35.3% - 64.7%)	166 (134,215)	130 (107,159)
Turkey	Upper middle	Default	67.6%	60.0% (53.0% - 66.4%)	60.0% (53.0% - 66.4%)	89 (64,127)	89 (64,127)
Turkey	Upper middle	2 dose threshold	73.6%	59.9% (52.9% - 66.4%)	59.9% (52.9% - 66.4%)	89 (64,127)	89 (64,127)
Turkey	Upper middle	40+ threshold	56.5%	59.5% (52.3% - 66.2%)	59.1% (51.7% - 66.2%)	88 (64,126)	88 (64,126)
Turkey	Upper middle	65+ threshold	55.0%	60.8% (54.3% - 66.9%)	60.1% (48.6% - 71.3%)	92 (67,130)	92 (66,126)

Country	Income bracket	Strategy	Proportion vaccinated	Proportion infected		Mortalities per 100,000	
				unchanged	adapted	unchanged	adapted
Turkey	Upper middle	Full sharing	53.5%	58.7% (50.0% - 66.4%)	46.1% (34.1% - 51.0%)	100 (74,134)	83 (59,115)
Uganda	Low	Default	3.3%	70.5% (62.2% - 78.6%)	70.5% (62.2% - 78.6%)	72 (56,87)	72 (56,87)
Uganda	Low	2 dose threshold	17.3%	70.4% (62.1% - 78.6%)	70.4% (62.1% - 78.6%)	71 (56,86)	71 (56,86)
Uganda	Low	40+ threshold	37.4%	69.8% (61.4% - 78.2%)	69.8% (61.6% - 78.2%)	67 (53,81)	67 (53,81)
Uganda	Low	65+ threshold	52.3%	61.7% (52.5% - 71.6%)	33.5% (23.8% - 47.6%)	39 (32,48)	26 (22,30)
Uganda	Low	Full sharing	55.1%	41.1% (32.3% - 51.9%)	25.0% (16.4% - 46.9%)	25 (20,30)	19 (14,29)
Ukraine	Lower middle	Default	6.8%	84.5% (75.9% - 91.3%)	84.5% (75.9% - 91.3%)	174 (118,241)	174 (118,241)
Ukraine	Lower middle	2 dose threshold	19.6%	84.3% (75.5% - 91.2%)	84.2% (75.4% - 91.1%)	173 (118,238)	173 (117,238)
Ukraine	Lower middle	40+ threshold	39.2%	83.7% (74.2% - 91.0%)	82.0% (71.4% - 89.7%)	163 (114,222)	162 (114,221)
Ukraine	Lower middle	65+ threshold	51.4%	79.7% (68.2% - 88.1%)	66.1% (55.6% - 75.9%)	150 (109,199)	146 (108,194)
Ukraine	Lower middle	Full sharing	53.1%	76.5% (63.1% - 85.4%)	55.3% (35.8% - 64.9%)	134 (99,176)	115 (85,142)
United Arab Emirates	High	Default	100.0%	24.1% (20.5% - 27.9%)	24.1% (20.5% - 27.9%)	52 (42,62)	52 (42,62)
United Arab Emirates	High	2 dose threshold	94.5%	24.6% (20.8% - 28.9%)	24.6% (20.8% - 28.5%)	52 (42,62)	52 (42,63)
United Arab Emirates	High	40+ threshold	48.1%	73.4% (72.2% - 75.1%)	41.2% (33.2% - 48.1%)	129 (94,176)	74 (56,100)
United Arab Emirates	High	65+ threshold	53.5%	71.5% (70.0% - 73.2%)	60.5% (47.7% - 67.1%)	151 (108,209)	125 (91,182)
United Arab Emirates	High	Full sharing	52.9%	64.5% (58.1% - 68.6%)	36.6% (29.7% - 48.9%)	184 (133,253)	85 (67,120)
United Kingdom	High	Default	98.2%	25.0% (22.8% - 27.7%)	25.0% (22.8% - 27.7%)	202 (176,232)	202 (176,232)
United Kingdom	High	2 dose threshold	92.8%	25.5% (22.9% - 29.9%)	24.3% (22.7% - 25.8%)	202 (176,232)	202 (176,232)
United Kingdom	High	40+ threshold	70.1%	78.4% (69.9% - 82.8%)	24.3% (22.7% - 25.7%)	219 (201,242)	202 (176,232)
United Kingdom	High	65+ threshold	59.3%	70.8% (68.2% - 72.6%)	27.7% (24.5% - 32.1%)	278 (245,323)	213 (187,248)
United Kingdom	High	Full sharing	52.9%	76.8% (74.3% - 79.8%)	19.0% (17.0% - 23.6%)	426 (374,504)	223 (192,258)
United States of America	High	Default	84.5%	49.5% (47.3% - 52.0%)	49.5% (47.3% - 52.0%)	192 (178,203)	192 (178,203)
United States of America	High	2 dose threshold	87.1%	48.9% (46.8% - 51.3%)	48.8% (46.7% - 51.2%)	192 (178,203)	192 (178,203)
United States of America	High	40+ threshold	68.4%	73.0% (70.4% - 75.4%)	34.4% (33.5% - 35.3%)	225 (208,240)	184 (171,196)
United States of America	High	65+ threshold	58.1%	81.5% (80.7% - 82.5%)	37.2% (34.9% - 40.8%)	245 (226,263)	188 (173,200)
United States of America	High	Full sharing	55.4%	82.5% (81.7% - 83.5%)	24.7% (21.6% - 37.1%)	270 (250,289)	162 (144,212)
Uruguay	High	Default	100.0%	43.8% (40.5% - 47.7%)	43.8% (40.5% - 47.7%)	87 (71,104)	87 (71,104)
Uruguay	High	2 dose threshold	91.3%	43.8% (40.4% - 47.6%)	43.8% (40.4% - 47.6%)	87 (71,104)	87 (71,104)
Uruguay	High	40+ threshold	66.0%	43.6% (40.2% - 47.4%)	43.6% (40.2% - 47.4%)	86 (71,103)	86 (71,103)
Uruguay	High	65+ threshold	57.2%	44.6% (41.5% - 47.9%)	45.9% (40.5% - 49.3%)	81 (67,98)	82 (66,100)
Uruguay	High	Full sharing	53.3%	41.0% (37.9% - 45.5%)	16.1% (9.8% - 21.4%)	71 (58,84)	22 (15,27)
Uzbekistan	Lower middle	Default	13.5%	62.2% (52.6% - 67.7%)	62.2% (52.6% - 67.7%)	198 (156,271)	198 (156,271)
Uzbekistan	Lower middle	2 dose threshold	26.1%	62.1% (52.6% - 67.6%)	62.1% (52.6% - 67.6%)	197 (155,269)	197 (155,270)
Uzbekistan	Lower middle	40+ threshold	42.1%	61.5% (51.8% - 67.1%)	61.4% (51.8% - 67.1%)	188 (149,255)	188 (148,256)
Uzbekistan	Lower middle	65+ threshold	51.3%	56.8% (46.5% - 63.8%)	56.4% (42.4% - 64.3%)	146 (117,195)	144 (115,185)

Country	Income bracket	Strategy	Proportion vaccinated	Proportion infected		Mortalities per 100,000	
				unchanged	adapted	unchanged	adapted
Uzbekistan	Lower middle	Full sharing	53.8%	49.5% (37.0% - 60.5%)	42.3% (33.3% - 51.2%)	121 (97,159)	118 (84,154)
Venezuela	Upper middle	Default	11.8%	26.0% (18.6% - 32.1%)	26.0% (18.6% - 32.1%)	25 (20,30)	25 (20,30)
Venezuela	Upper middle	2 dose threshold	24.7%	25.9% (18.6% - 32.1%)	26.0% (18.6% - 32.1%)	24 (20,30)	25 (20,30)
Venezuela	Upper middle	40+ threshold	42.6%	25.5% (18.4% - 31.7%)	25.5% (18.3% - 31.7%)	23 (19,28)	23 (19,29)
Venezuela	Upper middle	65+ threshold	51.7%	21.6% (15.0% - 27.5%)	26.3% (16.7% - 35.9%)	17 (14,20)	18 (15,23)
Venezuela	Upper middle	Full sharing	53.8%	16.6% (11.3% - 21.7%)	10.1% (6.3% - 22.1%)	13 (11,15)	8 (7,12)
Vietnam	Lower middle	Default	5.9%	24.8% (20.2% - 30.5%)	24.8% (20.2% - 30.5%)	52 (45,61)	52 (45,61)
Vietnam	Lower middle	2 dose threshold	19.3%	24.0% (19.6% - 29.6%)	24.0% (19.5% - 29.5%)	51 (44,59)	51 (44,59)
Vietnam	Lower middle	40+ threshold	39.3%	20.8% (16.6% - 25.8%)	19.7% (15.9% - 24.5%)	39 (33,45)	39 (34,45)
Vietnam	Lower middle	65+ threshold	51.1%	10.9% (8.0% - 15.0%)	11.7% (3.9% - 20.3%)	11 (9,12)	10 (5,14)
Vietnam	Lower middle	Full sharing	53.6%	3.0% (1.5% - 5.1%)	0.1% (0.1% - 0.1%)	2 (2,3)	0 (0,0)
Yemen	Low	Default	1.8%	56.2% (43.3% - 66.9%)	56.2% (43.3% - 66.9%)	106 (78,137)	106 (78,137)
Yemen	Low	2 dose threshold	15.8%	55.4% (42.7% - 66.0%)	55.3% (42.7% - 66.0%)	103 (75,134)	103 (75,134)
Yemen	Low	40+ threshold	36.3%	50.3% (38.6% - 59.9%)	49.6% (38.1% - 58.8%)	89 (67,112)	89 (67,111)
Yemen	Low	65+ threshold	51.9%	43.9% (32.2% - 53.3%)	43.6% (31.9% - 53.1%)	77 (60,96)	77 (60,95)
Yemen	Low	Full sharing	54.5%	41.4% (29.9% - 50.5%)	29.4% (20.1% - 37.5%)	59 (45,73)	44 (32,62)
Zimbabwe	Lower middle	Default	10.7%	77.4% (68.5% - 84.4%)	77.4% (68.5% - 84.4%)	77 (59,100)	77 (59,100)
Zimbabwe	Lower middle	2 dose threshold	23.7%	77.3% (68.4% - 84.3%)	77.3% (68.4% - 84.3%)	77 (58,100)	77 (58,100)
Zimbabwe	Lower middle	40+ threshold	41.8%	76.0% (66.6% - 83.4%)	76.0% (66.9% - 83.6%)	73 (55,93)	73 (55,93)
Zimbabwe	Lower middle	65+ threshold	52.4%	63.2% (51.2% - 73.2%)	43.5% (36.0% - 50.3%)	60 (47,76)	55 (43,71)
Zimbabwe	Lower middle	Full sharing	54.8%	47.7% (38.4% - 56.0%)	34.5% (21.1% - 52.1%)	51 (39,64)	35 (28,44)

Region	Strategy	Percentage reduced infection		Percentage reduced mortality	
		unchanged behaviour	adapted behaviour	unchanged behaviour	adapted behaviour
World	2 dose threshold	0.2 (0.2 – 0.3)	0.3 (0.3 – 0.4)	0.3 (0.2 – 0.3)	0.3 (0.3 – 0.3)
	40+ threshold	-1.8 (-2.1 – -1.5)	3.4 (3.0 – 3.7)	1.1 (0.9 – 1.4)	2.7 (2.5 – 2.9)
	65+ threshold	1.9 (1.2 – 2.8)	13.5 (12.3 – 15.5)	8.6 (8.1 – 9.4)	13.0 (12.3 – 13.8)
	Full sharing	8.1 (5.9 – 11.4)	40.2 (36.9 – 42.0)	13.3 (12.0 – 14.5)	37.3 (33.1 – 39.0)
Low income	2 dose threshold	0.4 (0.3 – 0.6)	0.4 (0.3 – 0.6)	1.0 (0.9 – 1.2)	1.0 (0.9 – 1.1)
	40+ threshold	3.2 (2.6 – 3.9)	3.5 (2.9 – 4.4)	9.0 (8.3 – 9.8)	9.0 (8.2 – 9.9)
	65+ threshold	15.1 (13.6 – 17.6)	24.5 (22.4 – 26.5)	27.3 (25.5 – 29.0)	30.5 (28.5 – 33.0)
	Full sharing	25.9 (23.9 – 28.1)	41.5 (34.5 – 46.4)	39.0 (37.3 – 40.8)	50.9 (46.7 – 54.2)
Lower middle income	2 dose threshold	0.2 (0.2 – 0.3)	0.3 (0.2 – 0.3)	0.4 (0.4 – 0.4)	0.4 (0.3 – 0.4)
	40+ threshold	1.7 (1.5 – 1.9)	2.0 (1.7 – 2.2)	2.7 (2.5 – 2.9)	2.7 (2.5 – 3.0)
	65+ threshold	6.9 (6.4 – 7.8)	9.1 (8.2 – 11.3)	13.6 (13.0 – 14.2)	14.5 (13.9 – 15.3)
	Full sharing	12.6 (11.0 – 14.5)	39.5 (35.4 – 42.3)	24.7 (24.2 – 25.6)	46.1 (40.8 – 48.8)
Higher middle income	2 dose threshold	0.1 (0.1 – 0.2)	0.1 (0.0 – 0.2)	0.2 (0.2 – 0.2)	0.2 (0.2 – 0.2)
	40+ threshold	1.0 (0.8 – 1.4)	1.2 (0.9 – 1.8)	2.4 (2.1 – 2.7)	2.4 (2.1 – 2.8)
	65+ threshold	6.4 (4.8 – 8.3)	17.9 (15.9 – 20.9)	11.7 (10.7 – 13.4)	15.0 (13.6 – 17.1)
	Full sharing	15.0 (9.5 – 21.6)	39.9 (34.8 – 44.5)	18.7 (16.5 – 20.9)	33.3 (27.8 – 36.9)
High income	2 dose threshold	0.3 (-0.0 – 0.6)	1.1 (0.8 – 1.3)	0.0 (-0.0 – 0.1)	0.1 (0.1 – 0.1)
	40+ threshold	-28.2 (-31.3 – -26.5)	13.9 (12.6 – 15.4)	-5.2 (-5.7 – -4.4)	1.9 (1.5 – 2.2)
	65+ threshold	-42.4 (-46.9 – -40.0)	12.3 (8.8 – 16.1)	-9.1 (-10.1 – -8.3)	3.5 (2.7 – 4.2)
	Full sharing	-42.7 (-45.6 – -38.5)	43.9 (32.5 – 48.3)	-22.3 (-23.7 – -20.6)	23.0 (13.7 – 26.6)

Table S4: Estimates for reductions in infection and mortality levels taken at the end of 2021 for central vaccine sharing strategies relative to the current scenario. Bracketed values represent a 95% prediction interval.

2 Lower level adapted behaviour

In tables S5 and S6 we present data for alternative simulations for scenarios with a lower level of adapted behaviour. As in the central adapted behaviour scenarios, we implement behavioural response by increasing/decreasing the control parameter for each country that is sharing vaccines, dependent on whether the number of active infections is increasing/decreasing (subject to a five day time lag to reflect delays in detection and reaction). However, in these alternative scenarios the control parameter is increased at 50% of the rate of the central scenarios.

Region	Strategy	Proportion vaccinated (2 doses)	Proportion infected		Mortalities per 100,000	
			unchanged behaviour	lower level adapted behaviour	unchanged behaviour	lower level adapted behaviour
World	Default	44.4%	48.4% (47.4% - 49.6%)	48.4% (47.4% - 49.6%)	133.1 (128.2 - 138.3)	133.1 (128.2 - 138.3)
	2 dose threshold	49.6%	48.3% (47.3% - 49.6%)	48.3% (47.3% - 49.5%)	132.7 (127.8 - 137.9)	132.8 (127.9 - 137.9)
	40+ threshold	49.6%	49.3% (48.2% - 50.6%)	46.9% (46.0% - 48.0%)	131.6 (126.8 - 136.5)	129.7 (125.0 - 134.8)
	65+ threshold	49.4%	47.5% (46.1% - 49.1%)	43.3% (41.9% - 44.7%)	121.6 (117.7 - 126.5)	117.3 (113.6 - 122.0)
	Full sharing	49.4%	44.4% (42.0% - 46.7%)	32.6% (31.4% - 34.6%)	115.4 (112.5 - 119.3)	93.9 (88.2 - 99.4)
Low income	Default	1.7%	64.6% (61.6% - 66.9%)	64.6% (61.6% - 66.9%)	64.0 (59.4 - 70.1)	64.0 (59.4 - 70.1)
	2 dose threshold	11.7%	64.3% (61.3% - 66.6%)	64.3% (61.4% - 66.6%)	63.4 (58.8 - 69.3)	63.4 (58.8 - 69.5)
	40+ threshold	26.9%	62.5% (59.4% - 64.8%)	62.4% (59.4% - 64.9%)	58.3 (53.9 - 63.9)	58.4 (54.2 - 63.9)
	65+ threshold	38.7%	54.7% (51.5% - 57.2%)	50.2% (47.6% - 52.9%)	46.5 (43.4 - 49.8)	44.8 (42.1 - 47.9)
	Full sharing	40.7%	47.8% (45.2% - 50.7%)	43.5% (38.7% - 47.8%)	39.1 (36.5 - 41.5)	34.8 (31.5 - 37.9)
Lower middle income	Default	18.4%	65.4% (63.2% - 68.1%)	65.4% (63.2% - 68.1%)	155.8 (144.2 - 169.7)	155.8 (144.2 - 169.7)
	2 dose threshold	28.5%	65.2% (63.1% - 67.9%)	65.2% (63.1% - 67.9%)	155.2 (143.6 - 169.1)	155.2 (143.6 - 169.2)
	40+ threshold	39.4%	64.2% (61.9% - 66.9%)	64.2% (62.1% - 66.7%)	151.5 (139.9 - 165.5)	151.7 (140.1 - 165.8)
	65+ threshold	46.2%	60.8% (58.2% - 63.6%)	60.2% (57.6% - 63.2%)	134.6 (124.1 - 148.0)	134.1 (123.8 - 146.9)
	Full sharing	47.4%	57.1% (54.0% - 60.4%)	43.8% (41.8% - 46.4%)	117.1 (107.9 - 128.9)	91.9 (81.7 - 103.2)
Higher middle income	Default	66.0%	33.8% (32.5% - 34.7%)	33.8% (32.5% - 34.7%)	105.9 (100.3 - 113.0)	105.9 (100.3 - 113.0)
	2 dose threshold	66.1%	33.8% (32.5% - 34.7%)	33.8% (32.5% - 34.7%)	105.7 (100.1 - 112.8)	105.7 (100.2 - 112.8)
	40+ threshold	56.6%	33.5% (32.1% - 34.5%)	33.5% (32.2% - 34.4%)	103.4 (98.1 - 110.0)	103.5 (98.3 - 110.5)
	65+ threshold	51.6%	31.7% (29.9% - 32.9%)	29.1% (27.9% - 30.2%)	93.4 (89.0 - 98.5)	91.0 (87.0 - 95.4)
	Full sharing	51.4%	28.6% (25.6% - 31.0%)	22.8% (21.3% - 24.7%)	86.0 (81.8 - 90.0)	78.4 (74.3 - 83.4)
High income	Default	75.3%	35.7% (34.5% - 36.7%)	35.7% (34.5% - 36.7%)	162.9 (155.7 - 168.9)	162.9 (155.7 - 168.9)
	2 dose threshold	78.8%	35.6% (34.4% - 36.6%)	35.3% (34.2% - 36.4%)	162.9 (155.7 - 168.9)	162.8 (155.6 - 168.8)
	40+ threshold	68.5%	45.7% (44.5% - 47.0%)	31.0% (30.3% - 31.6%)	171.3 (164.1 - 177.6)	160.1 (152.8 - 165.8)
	65+ threshold	57.3%	50.9% (49.8% - 52.0%)	34.2% (32.0% - 37.2%)	177.8 (170.2 - 184.8)	160.3 (153.5 - 166.9)
	Full sharing	53.6%	50.8% (48.5% - 52.4%)	24.7% (21.8% - 30.4%)	199.1 (190.4 - 208.0)	149.5 (134.6 - 169.4)

Table S5: Estimates for vaccination coverage, proportion infected and mortality rates (per 100,000) for each income group for strategies with a lower level of behaviour adaptation than in the central scenarios. All values are taken at the start of 2022. Bracketed values represent a 95% prediction interval, proportion vaccinated is deterministic and so an interval is not provided.

Region	Strategy	Percentage reduced infection		Percentage reduced mortality	
		unchanged behaviour	lower level adapted behaviour	unchanged behaviour	lower level adapted behaviour
World	2 dose threshold	0.2 (0.2 – 0.3)	0.3 (0.2 – 0.4)	0.3 (0.2 – 0.3)	0.3 (0.2 – 0.3)
	40+ threshold	-1.8 (-2.1 – -1.5)	3.1 (2.7 – 3.5)	1.1 (0.9 – 1.4)	2.5 (2.4 – 2.8)
	65+ threshold	1.9 (1.2 – 2.8)	10.5 (9.2 – 12.3)	8.6 (8.1 – 9.4)	11.9 (11.0 – 13.0)
	Full sharing	8.1 (5.9 – 11.4)	32.9 (29.5 – 35.4)	13.3 (12.0 – 14.5)	29.9 (25.0 – 32.1)
Low income	2 dose threshold	0.4 (0.3 – 0.6)	0.4 (0.2 – 0.5)	1.0 (0.9 – 1.2)	1.0 (0.8 – 1.1)
	40+ threshold	3.2 (2.6 – 3.9)	3.4 (2.5 – 4.2)	9.0 (8.3 – 9.8)	8.8 (7.7 – 9.6)
	65+ threshold	15.1 (13.6 – 17.6)	22.2 (20.1 – 24.7)	27.3 (25.5 – 29.0)	30.1 (28.0 – 32.4)
	Full sharing	25.9 (23.9 – 28.1)	32.5 (27.3 – 38.9)	39.0 (37.3 – 40.8)	45.6 (41.6 – 49.7)
Lower middle income	2 dose threshold	0.2 (0.2 – 0.3)	0.2 (0.2 – 0.3)	0.4 (0.4 – 0.4)	0.4 (0.3 – 0.4)
	40+ threshold	1.7 (1.5 – 1.9)	1.9 (1.5 – 2.1)	2.7 (2.5 – 2.9)	2.6 (2.4 – 2.9)
	65+ threshold	6.9 (6.4 – 7.8)	7.7 (7.0 – 9.5)	13.6 (13.0 – 14.2)	13.9 (13.2 – 14.6)
	Full sharing	12.6 (11.0 – 14.5)	33.1 (29.5 – 36.8)	24.7 (24.2 – 25.6)	41.5 (36.4 – 44.6)
Higher middle income	2 dose threshold	0.1 (0.1 – 0.2)	0.1 (-0.0 – 0.2)	0.2 (0.2 – 0.2)	0.2 (0.1 – 0.2)
	40+ threshold	1.0 (0.8 – 1.4)	1.0 (0.7 – 1.6)	2.4 (2.1 – 2.7)	2.2 (2.0 – 2.6)
	65+ threshold	6.4 (4.8 – 8.3)	13.8 (11.8 – 16.1)	11.7 (10.7 – 13.4)	13.9 (12.5 – 15.8)
	Full sharing	15.0 (9.5 – 21.6)	33.2 (27.4 – 36.2)	18.7 (16.5 – 20.9)	25.9 (22.8 – 29.3)
High income	2 dose threshold	0.3 (-0.0 – 0.6)	1.0 (0.6 – 1.2)	0.0 (-0.0 – 0.1)	0.1 (-0.0 – 0.1)
	40+ threshold	-28.2 (-31.3 – -26.5)	13.1 (11.4 – 14.7)	-5.2 (-5.7 – -4.4)	1.7 (1.4 – 2.0)
	65+ threshold	-42.4 (-46.9 – -40.0)	4.9 (-6.2 – 10.6)	-9.1 (-10.1 – -8.3)	1.6 (-0.5 – 3.0)
	Full sharing	-42.7 (-45.6 – -38.5)	32.1 (15.4 – 39.3)	-22.3 (-23.7 – -20.6)	9.0 (-4.7 – 17.1)

Table S6: Estimates for reductions in infection and mortality levels taken at the end of 2021 relative to the current scenario, with alternative lower level adapted behaviour. Bracketed values represent a 95% prediction interval.

3 Age biased sharing strategies

In tables S7 and S8 we present data for alternative strategies where vaccine redistribution is age biased, dependent on the number of unvaccinated individuals in each 5 year age bracket weighted by vulnerability (in the central scenarios vaccine redistribution is unweighted, proportional to the size of the eligible adult population of each country that is as yet unvaccinated).

Region	Strategy	Proportion vaccinated (2 doses)	Proportion infected unchanged behaviour (age biased)	Proportion infected adapted behaviour (age biased)	Mortalities per 100,000 unchanged behaviour (age biased)	Mortalities per 100,000 adapted behaviour (age biased)
World	Default	44.4%	48.4% (47.4% - 49.6%)	48.4% (47.4% - 49.6%)	133.1 (128.2 - 138.3)	133.1 (128.2 - 138.3)
	2 dose threshold	49.4%	48.4% (47.3% - 49.6%)	48.3% (47.3% - 49.5%)	132.8 (127.8 - 137.9)	132.8 (127.8 - 137.9)
	40+ threshold	49.2%	49.5% (48.4% - 50.6%)	46.8% (45.8% - 48.0%)	131.7 (127.0 - 136.7)	129.5 (124.6 - 134.4)
	65+ threshold	49.0%	48.0% (46.6% - 49.4%)	42.2% (40.8% - 43.7%)	123.0 (119.1 - 127.8)	117.1 (113.1 - 122.1)
	Full sharing	48.9%	46.8% (45.3% - 48.5%)	29.6% (28.2% - 31.6%)	119.8 (116.2 - 124.5)	85.5 (81.1 - 90.5)
Low income	Default	1.7%	64.6% (61.6% - 66.9%)	64.6% (61.6% - 66.9%)	64.0 (59.4 - 70.1)	64.0 (59.4 - 70.1)
	2 dose threshold	12.0%	64.3% (61.3% - 66.6%)	64.3% (61.3% - 66.6%)	63.5 (58.9 - 69.6)	63.5 (58.9 - 69.6)
	40+ threshold	25.8%	62.8% (59.8% - 65.3%)	62.5% (59.5% - 64.9%)	59.2 (54.9 - 65.1)	59.2 (54.9 - 65.2)
	65+ threshold	29.7%	57.9% (55.0% - 60.5%)	52.5% (49.8% - 54.9%)	49.7 (46.4 - 53.6)	47.6 (44.3 - 50.8)
	Full sharing	29.8%	55.1% (52.1% - 57.7%)	38.6% (34.0% - 43.4%)	44.9 (42.1 - 48.0)	33.5 (30.0 - 36.9)
Lower middle income	Default	18.4%	65.4% (63.2% - 68.1%)	65.4% (63.2% - 68.1%)	155.8 (144.2 - 169.7)	155.8 (144.2 - 169.7)
	2 dose threshold	27.8%	65.2% (63.1% - 67.9%)	65.2% (63.1% - 67.9%)	155.2 (143.7 - 169.2)	155.2 (143.7 - 169.2)
	40+ threshold	39.6%	64.2% (61.9% - 66.9%)	64.1% (61.8% - 66.7%)	151.8 (140.3 - 165.8)	151.8 (140.0 - 165.9)
	65+ threshold	41.4%	61.7% (59.2% - 64.5%)	59.8% (57.1% - 62.5%)	137.8 (127.0 - 151.3)	135.9 (125.5 - 148.9)
	Full sharing	41.4%	60.4% (57.9% - 63.2%)	41.0% (38.9% - 43.7%)	128.9 (118.6 - 141.3)	88.8 (79.1 - 98.9)
Higher middle income	Default	66.0%	33.8% (32.5% - 34.7%)	33.8% (32.5% - 34.7%)	105.9 (100.3 - 113.0)	105.9 (100.3 - 113.0)
	2 dose threshold	66.1%	33.8% (32.5% - 34.7%)	33.8% (32.5% - 34.7%)	105.7 (100.1 - 112.8)	105.7 (100.2 - 112.8)
	40+ threshold	56.1%	33.5% (32.2% - 34.4%)	33.4% (32.1% - 34.4%)	103.3 (98.0 - 110.0)	103.3 (98.0 - 109.8)
	65+ threshold	54.2%	32.0% (30.5% - 33.2%)	27.7% (26.4% - 28.8%)	94.4 (89.8 - 99.9)	90.2 (85.9 - 94.9)
	Full sharing	54.1%	31.1% (29.3% - 32.6%)	20.5% (18.4% - 23.0%)	89.8 (85.7 - 95.1)	71.0 (65.9 - 76.7)
High income	Default	75.3%	35.7% (34.5% - 36.7%)	35.7% (34.5% - 36.7%)	162.9 (155.7 - 168.9)	162.9 (155.7 - 168.9)
	2 dose threshold	78.8%	35.7% (34.5% - 36.7%)	35.4% (34.2% - 36.4%)	162.8 (155.6 - 168.7)	162.6 (155.5 - 168.6)
	40+ threshold	67.2%	46.5% (45.1% - 47.7%)	30.6% (29.9% - 31.3%)	171.3 (164.2 - 177.4)	158.7 (151.4 - 164.4)
	65+ threshold	64.1%	48.9% (47.8% - 49.9%)	30.8% (29.6% - 31.9%)	175.4 (167.7 - 182.2)	156.1 (149.2 - 161.7)
	Full sharing	63.9%	48.3% (47.3% - 49.2%)	20.6% (18.7% - 23.8%)	188.1 (179.6 - 196.2)	125.4 (118.0 - 137.9)

Table S7: Estimates for vaccination coverage, proportion infected and mortality rates (per 100,000) for each income group for age biased sharing strategies. All values are taken at the start of 2022. Bracketed values represent a 95% prediction interval, proportion vaccinated is deterministic and so an interval is not provided.

Region	Strategy	Percentage reduced infection		Percentage reduced mortality	
	(age biased)	unchanged behaviour (age biased)	adapted behaviour (age biased)	unchanged behaviour (age biased)	adapted behaviour (age biased)
World	2 dose threshold	0.2 (0.1 – 0.3)	0.3 (0.2 – 0.4)	0.3 (0.2 – 0.3)	0.3 (0.2 – 0.3)
	40+ threshold	-2.1 (-2.4 – -1.8)	3.3 (3.0 – 3.7)	1.0 (0.8 – 1.3)	2.8 (2.5 – 3.0)
	65+ threshold	1.0 (0.5 – 1.8)	12.7 (11.6 – 14.4)	7.6 (7.1 – 8.3)	12.0 (11.3 – 12.8)
	Full sharing	3.4 (2.5 – 4.5)	39.0 (36.1 – 41.2)	10.0 (9.2 – 10.8)	36.1 (32.5 – 38.2)
Low income	2 dose threshold	0.4 (0.2 – 0.5)	0.4 (0.3 – 0.5)	0.8 (0.6 – 0.9)	0.8 (0.7 – 0.9)
	40+ threshold	2.7 (2.2 – 3.3)	3.2 (2.5 – 3.9)	7.5 (6.9 – 8.5)	7.6 (6.7 – 8.5)
	65+ threshold	10.1 (9.0 – 11.7)	18.6 (16.8 – 21.3)	22.4 (20.6 – 24.0)	25.6 (23.8 – 27.6)
	Full sharing	14.3 (12.8 – 17.3)	40.5 (33.8 – 46.2)	29.9 (28.5 – 31.5)	47.7 (41.4 – 52.3)
Lower middle income	2 dose threshold	0.3 (0.2 – 0.3)	0.3 (0.2 – 0.3)	0.3 (0.3 – 0.4)	0.3 (0.3 – 0.4)
	40+ threshold	1.8 (1.5 – 2.0)	2.0 (1.7 – 2.2)	2.6 (2.4 – 2.8)	2.6 (2.3 – 2.9)
	65+ threshold	5.6 (5.2 – 6.4)	8.3 (7.7 – 10.5)	11.5 (11.0 – 12.2)	12.7 (12.1 – 13.5)
	Full sharing	7.5 (6.9 – 8.6)	37.6 (34.2 – 40.6)	17.2 (16.6 – 18.0)	43.6 (37.8 – 46.5)
Higher middle income	2 dose threshold	0.1 (0.0 – 0.2)	0.1 (0.0 – 0.2)	0.2 (0.2 – 0.2)	0.2 (0.2 – 0.2)
	40+ threshold	1.0 (0.8 – 1.3)	1.2 (0.9 – 1.8)	2.4 (2.2 – 2.8)	2.5 (2.2 – 2.9)
	65+ threshold	5.2 (4.1 – 6.9)	18.2 (16.0 – 20.1)	10.8 (9.8 – 12.2)	14.6 (13.2 – 16.7)
	Full sharing	8.0 (5.9 – 10.4)	40.0 (32.5 – 43.7)	15.1 (13.8 – 16.7)	33.0 (27.6 – 37.1)
High income	2 dose threshold	-0.0 (-0.4 – 0.1)	0.7 (0.5 – 1.0)	0.1 (0.0 – 0.1)	0.2 (0.1 – 0.2)
	40+ threshold	-30.3 (-33.7 – -28.0)	14.2 (12.9 – 15.8)	-5.2 (-5.8 – -4.3)	2.6 (2.3 – 2.8)
	65+ threshold	-36.9 (-40.3 – -35.1)	13.3 (10.2 – 17.3)	-7.6 (-8.5 – -6.8)	4.1 (3.5 – 4.9)
	Full sharing	-35.1 (-39.2 – -33.3)	43.1 (32.6 – 47.1)	-15.5 (-16.8 – -14.1)	23.4 (16.6 – 27.4)

Table S8: Estimates for reductions in infection and mortality levels taken at the end of 2021 for age biased vaccine sharing strategies relative to the current scenario. Bracketed values represent a 95% prediction interval.
